# Modeling islet enhancers using deep learning identifies candidate causal variants at loci associated with T2D and glycemic traits

**DOI:** 10.1073/pnas.2206612120

**Published:** 2023-08-21

**Authors:** Sanjarbek Hudaiberdiev, D. Leland Taylor, Wei Song, Narisu Narisu, Redwan M. Bhuiyan, Henry J. Taylor, Xuming Tang, Tingfen Yan, Amy J. Swift, Lori L. Bonnycastle, DIAMANTE Consortium, Shuibing Chen, Michael L. Stitzel, Michael R. Erdos, Ivan Ovcharenko, Francis S. Collins

**Affiliations:** ^a^Computational Biology Branch, National Center for Biotechnology Information, National Library of Medicine, NIH, Bethesda, MD 20892; ^b^Center for Precision Health Research, National Human Genome Research Institute, NIH, Bethesda, MD 20892; ^c^The Jackson Laboratory for Genomic Medicine, Farmington, CT 06032; ^d^Department of Genetics and Genome Sciences, University of Connecticut, Farmington, CT 06032; ^e^British Heart Foundation Cardiovascular Epidemiology Unit, Department of Public Health and Primary Care, University of Cambridge, Cambridge CB1 8RN, UK; ^f^Department of Surgery, Weill Cornell Medicine, New York, NY 10065; ^g^Center for Genomic Health, Weill Cornell Medicine, New York, NY 10065; ^h^Institute of Systems Genomics, University of Connecticut, Farmington, CT 06032

**Keywords:** deep learning, enhancer, pancreatic islets, type 2 diabetes, epigenomics

## Abstract

Genetic association studies have identified hundreds of independent signals associated with type 2 diabetes (T2D) and related traits. Despite these successes, the identification of specific causal variants underlying a genetic association signal remains challenging. In this study, we describe a deep learning (DL) method to analyze the impact of sequence variants on enhancers. Focusing on pancreatic islets, a T2D relevant tissue, we show that our model learns islet-specific transcription factor (TF) regulatory patterns and can be used to prioritize candidate causal variants. At 101 genetic signals associated with T2D and related glycemic traits where multiple variants occur in linkage disequilibrium, our method nominates a single causal variant for each association signal, including three variants previously shown to alter reporter activity in islet-relevant cell types. For another signal associated with blood glucose levels, we biochemically test all candidate causal variants from statistical fine-mapping using a pancreatic islet beta cell line and show biochemical evidence of allelic effects on TF binding for the model-prioritized variant. To aid in future research, we publicly distribute our model and islet enhancer perturbation scores across ~67 million genetic variants. We anticipate that DL methods like the one presented in this study will enhance the prioritization of candidate causal variants for functional studies.

## Significance

Identifying the genomic and molecular effects of disease-associated genetic variants is a central challenge in translating signals from genetic association studies to insights into the causes of disease. Such effects can be defined by targeted functional studies, but these studies are difficult to scale across the thousands of candidate causal variants routinely identified by genetic association studies. To help solve this problem, we developed a method to predict the effects of genetic variation on enhancers. We apply this method to model pancreatic islet enhancers, demonstrate that the model is accurate, and show that the predicted effects of genetic variants on enhancers can help identify candidate causal variants for targeted functional studies.

Over the past two decades, immense progress has been made toward unraveling the genetic basis of diseases and traits, with tens of thousands of genetic associations identified to date (1). These associations could guide advances toward effective treatment and prevention of disease by shedding light on the underlying disease etiology and pinpointing specific genes, cell types, and molecular pathways that contribute to a disease. However, despite a few notable examples (2, 3), only modest progress has been made in translating genetic discoveries about common diseases into therapies.

This challenge of translation is driven in part by the difficulties in i) identifying which variants influence disease, since most disease-associated genetic signals are composed of many candidate causal single nucleotide polymorphisms (SNPs) due to linkage disequilibrium (LD), and ii) establishing how these variants mechanistically function. To date, a variety of approaches (reviewed in ref. 4) have been developed to prioritize candidate causal SNPs by weighting SNPs according to their statistical evidence of association (statistical fine-mapping) and by the functional/epigenomic signals overlapping a SNP (functional fine-mapping). However, such methods often fail to nominate a feasible number of SNP candidates to test in the laboratory due to i) limited statistical power to disentangle the effects of correlated SNPs even at large sample sizes and ii) no clear metric to weight epigenomic overlaps in the case of functional fine-mapping.

Type 2 diabetes (T2D) is an exemplary case of the challenges of identifying causal variants and effector genes. T2D is a disease characterized by pancreatic islet beta cell dysfunction and insulin resistance in peripheral tissues (5). In a recent fine-mapping analysis of 898,130 European-descent participants, 243 loci were associated with T2D. These loci contained 403 distinct association signals (multiple, independent signals per locus), of which 18 signals could be narrowed down to one SNP based on statistical fine-mapping (6). Given the strong enrichment of T2D genetic signals in regulatory regions (e.g., enhancers) active in islets (reviewed in ref. 7), the authors performed functional fine-mapping of these 403 signals using islet epigenomic information and refined this list to 23 signals with one SNP, leaving much room for improvement.

**Fig. 1.**
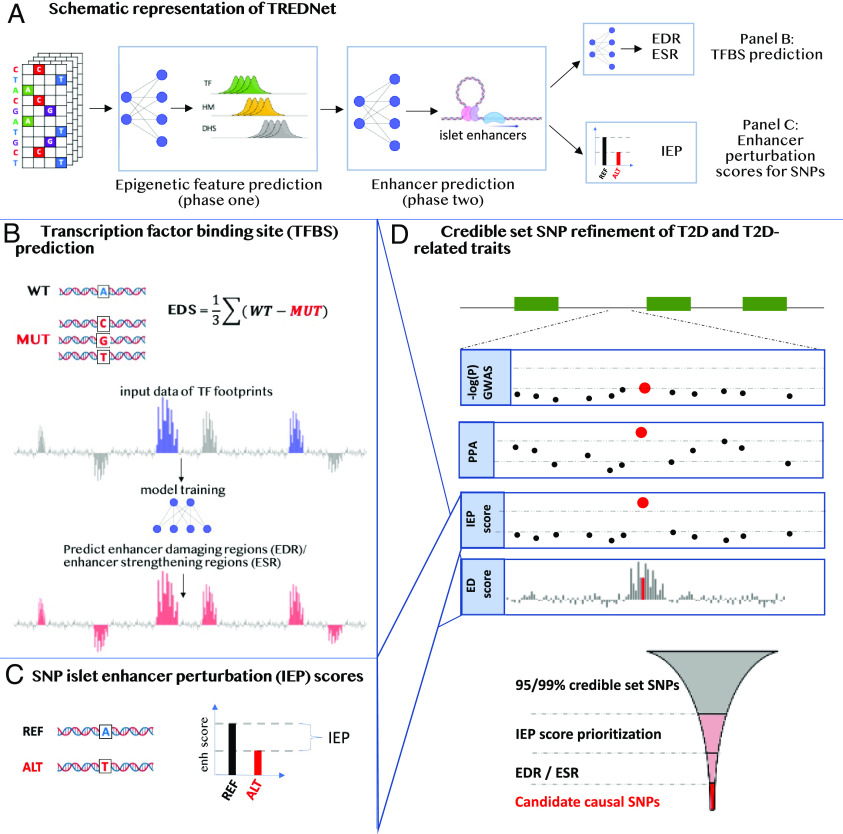
Graphical overview of this study. (*A*) Overview of TREDNet. TREDNet consists of two convolutional neural networks (CNNs; mesh of gray lines and blue circles). The first CNN is trained on genomic regions in one-hot encoded representation to predict peaks of epigenomic features, including TFs, histone modifications (HMs), and DNase I hypersensitivity sites (DHSs). The second CNN is trained on the output from the first CNN to predict enhancer regions. Enhancer graphic created with BioRender.com. (*B*) Saturated mutagenesis analysis using TREDNet produces enhancer damage (ED) scores, which are used to predict TF-binding sites (TFBSs), corresponding to peaks (enhancer damaging regions; EDRs) and dips (enhancer strengthening regions; ESRs) in ED scores. Bars depict ED scores of each genomic position (*x* axis). Blue bars show positions corresponding to known TFBSs. Red bars show TFBSs predicted by a CNN (mesh of gray lines and blue circles) using ED scores. (*C*) Allelic differences in TREDNet enhancer probability scores are used to calculate islet enhancer perturbation (IEP) scores for each SNP. (*D*) Schematic locus zoom example at a genetic signal where a candidate causal SNP is identified. Green boxes depict gene coding regions along the genome (*x* axis). Subsequent facets show different signals for each SNP (points): the −log_10_(*P*) of the genetic association, the posterior probability of association (PPA) from statistical fine-mapping, IEP scores, and ED scores. Funnel schematic describes the framework used to identify candidate causal SNPs. SNPs from 95/99% credible sets are prioritized using IEP scores. Subsequently, SNPs are prioritized by EDR/ESR overlap.

In this study (overview in Fig. 1), we report a deep learning (DL) method that models both shared and tissue-specific genomic and epigenomic signals to predict enhancers. Using this method, we analyze the impact of mutational profiles on pancreatic islet enhancers within the context of their local, surrounding DNA sequence. We show that our model learns islet-specific transcription factor (TF) regulatory patterns and can be used to i) predict TF-binding sites and ii) refine fine-mapping results by prioritizing candidate causal SNPs. By applying our model to prioritize SNPs from statistical fine-mapping results for T2D and related traits, we nominate a single candidate SNP that likely affects pancreatic islet enhancers at 101 signals containing more than one 95/99% credible set SNP from statistical fine-mapping. For three signals, previous studies validate our SNP predictions by showing these SNPs induce allelic activity in reporter assays in islet-relevant cell types. For another signal associated with blood glucose levels (near *PSMA1*), we biochemically demonstrate using a pancreatic islet beta cell line that the SNP prioritized by our model shows the greatest allelic effects on i) TF binding and ii) the regulation of luciferase reporter expression among all SNPs in the credible set. We believe models like the one presented in this study will aid in refining candidate causal SNPs from fine-mapping for further functional studies across a wide variety of diseases/traits.

## Results

### TREDNet: A DL Model for Enhancer Prediction.

To predict enhancers based on DNA sequence, we developed TREDNet, a two-phase DL framework consisting of two consecutive convolutional neural networks (CNNs): the first to predict epigenomic signals across the genome and the second to predict enhancers, the primary aim of TREDNet (*Materials and Methods* and Fig. 1*A*).

For the phase one model, we trained a CNN that uses tiled DNA sequences of 2,000 base pairs (bp) to predict DNase I hypersensitive sites (DHSs), histone modifications (HMs), and TF-binding sites (TFBSs) across 127 human cell types and tissues (biospecimens) from the ENCODE (8) and NIH Roadmap (9) studies (1,924 features in total). We excluded signals on chromosomes 8 and 9 from training and used them to test the model’s accuracy. We found that the phase one TREDNet model was highly accurate, achieving an average area under the receiver operating characteristic (auROC) of 0.93, 0.88, and 0.96 for DHSs, HMs, and TFBSs, respectively (*SI Appendix*, Fig. S1). We compared TREDNet’s phase one model to other methods that predict epigenomic signals from DNA sequences—ExPecto (10), DeepSEA (11), and Basset (12)—and found TREDNet performed similarly to these previous models (*SI Appendix*, Fig. S1).

**Fig. 2.**
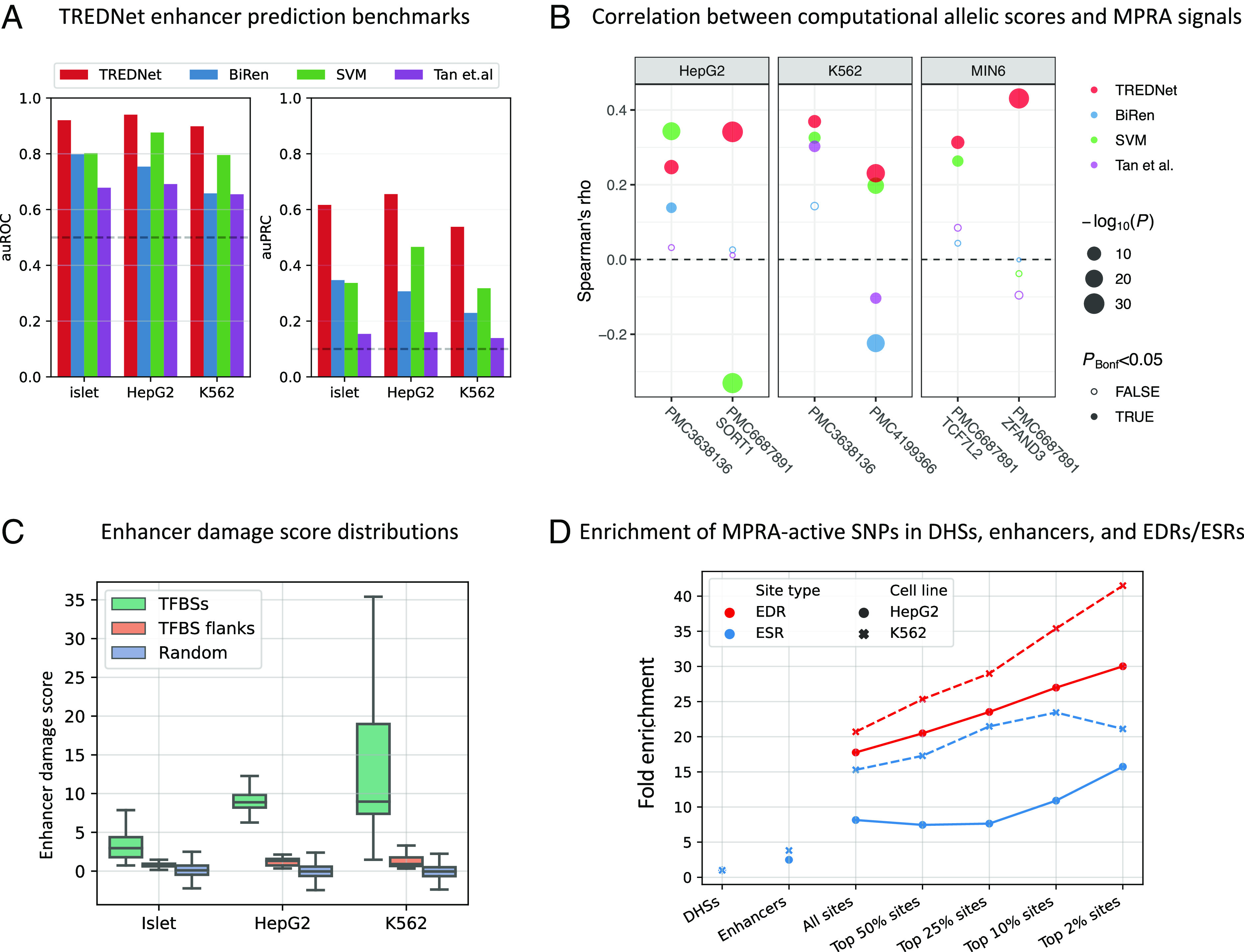
Characterization of TREDNet, TREDNet ED scores, and EDRs/ESRs. (*A*) Phase two TREDNet enhancer prediction accuracy across biospecimens (*x* axis) compared to other models (colors) using auROC (*Left*) and area under the precision recall curve (auPRC; *Right*) metrics (*y* axis). Dashed horizontal lines show the performance of a random classifier: auROC = 0.5 and auPRC = 0.09. (*B*) Correlation (Spearman’s rho; *y* axis) between predictions of computational methods (colors) and MPRA signals from different experiments (*x* axis; coded using PubMed Central identifiers) across biospecimens (facets). (*C*) Distribution of TREDNet ED scores (*y* axis) in TFBSs, TFBS flanking regions, and random genomic regions outside of TFBSs (colors) across biospecimens (*x* axis). (*D*) Enrichment (*y* axis) of active SNPs from HepG2 and K562 MPRA experiments (point shape and linetype) in EDRs/ESRs (colors), enhancers, and DHSs (*x* axis). EDRs/ESRs are binned into five groups by their average ED scores.

Using the vectors of 1,924 epigenomic predictions generated by the phase one model for each 2,000 bp DNA sequence, we trained a second (phase two) CNN to predict pancreatic islet enhancers—defined using chromatin accessibility profiles (assay for transposase-accessible chromatin with sequencing [ATAC-seq] peaks) and H3K27ac histone marks (*Materials and Methods*). We trained two similar models to predict HepG2 and K562 enhancers (one for each cell line) to validate our approach using datasets available for these two cell lines only. The result of this two-phase learning framework is the enhancer probability of a 2,000 bp DNA sequence. We used enhancer coordinates from chromosomes 8 and 9 withheld from training for validation and found that the phase two TREDNet model achieved an auROC of 0.92, 0.89, and 0.85 for islets, HepG2, and K562, respectively (Fig. 2*A*). As a benchmark, we compared TREDNet to other models that predict enhancers: BiRen (13), Tan et al. (14), and SVM (15). We found that TREDNet outperformed the other models consistently (Fig. 2*A*).

To further validate TREDNet’s enhancer probability scores, we used massively parallel reporter assay (MPRA) experiments in HepG2, K562, and MIN6, a mouse beta cell line (16–18). For each experimentally tested sequence, we compared the in vitro gene expression MPRA results to the enhancer probability predictions generated by TREDNet and the other enhancer prediction methods (*Materials and Methods*). We observed a strong, positive correlation between TREDNet’s predictions and the measured MPRA effects (minimum rho = 0.23, *P* < 1.77 × 10^−8^ across all datasets, Spearman’s rank-order correlation; Fig. 2*B*). Across these datasets, TREDNet either outperformed or exhibited near equal performance to other methods as quantified by Spearman’s rank-order correlation (Fig. 2*B*) and root mean squared error (*SI Appendix*, Fig. S4). We note that in the case of BiRen and Tan et al., we were not able to refit the model using the enhancer definitions from TREDNet and used the pretrained model distributed by the authors (*Materials and Methods*). For tissue/cell types poorly represented in the training data, like islet beta cells, the architecture may perform better if retrained.

### In Silico Saturated Mutagenesis of Enhancers Reveals TF Regulatory Patterns.

In order to probe the regulatory structure of enhancers, we performed an in silico saturated mutagenesis experiment across the DNA sequences of all islet, HepG2, and K562 enhancers, predicting the effects of nucleotide mutations on the overall enhancer probability of the surrounding 2,000 bp region (*Materials and Methods*). For every DNA sequence position within an enhancer, we calculated TREDNet enhancer damage (ED) scores, defined as the average difference in the enhancer probability of the reference nucleotide (GRCh37) and the enhancer probabilities of all other possible nucleotides. A positive ED score indicates a negative change in enhancer probability (enhancer damaging), while a negative ED score indicates a positive change in enhancer probability (enhancer strengthening).

Next, we asked if ED scores mark TFBSs, as has been suggested by previous studies that used similar metrics (reviewed in ref. 19). Using TFBSs from HepG2 ChIP-seq experiments (8), K562 ChIP-seq experiments (8), and islet ATAC-seq footprints (20) (since islets do not have as comprehensive of TF ChIP-seq profiles like HepG2 and K562), we compared the absolute value of ED scores within TFBSs to 20 bp regions immediately flanking each TFBS as well as randomly sampled enhancer regions (*Materials and Methods*). We found that the absolute value of ED scores of regions within TFBSs were much greater than flanking regions (average sevenfold increase; *P* < 1 × 10^−100^, Wilcoxon rank sum test) or randomly sampled regions (average 35-fold increase; *P* < 1 × 10^−100^, Wilcoxon rank sum test; Fig. 2*C*)—confirming that elevated ED scores differentiate TFBSs.

To explore if the TFBSs identified by ED scores are relevant to a tissue/cell type, we focused on islets and ranked each TF footprint by the ratio of the average ED score within the TF footprint motif to the flanking region (*SI Appendix*, Table S1). The top five TFs were all known to play an important regulatory role in islets: TCF7L2 (21), the FOX family of TFs (22, 23), the C/EBP family of TFs (24), the HNF family of TFs (25), and DBP (26). Moreover, across all islet TFBSs, we found that the ED scores were strongly correlated with the per-nucleotide information content of each TFBS motif (average Spearman’s rho=0.45)—much more than the evolutionary sequence conservation (average Spearman’s rho = 0.20; *P* = 1.8 × 10^−14^, Wilcoxon rank sum test; *SI Appendix*, Fig. S5; *Materials and Methods*). These trends held true for both HepG2 and K562 (*SI Appendix*, Fig. S5*B*). Combined, these results strongly suggest that TFBSs with the largest ED scores demarcate TFBSs important to a cell type and thereby allow one to predict regions within enhancers with the greatest effect when altered in the relevant cell type.

Given the observed link between ED scores and TFBSs, we designed another DL model to predict TFBSs directly from ED score profiles (*Materials and Methods*). For each biospecimen, we trained two separate models to predict TFBSs from either ChIP-seq experiments (HepG2 and K562) or ATAC-seq footprints (islets) as short stretches (≥3 bp; average length 13.7 bp) of either enhancer damaging regions (EDRs) or enhancer strengthening regions (ESRs; *SI Appendix*, Fig. S6). The resulting models were highly accurate at predicting TFBSs on chromosomes 8 and 9 (excluded from training), achieving an average auROC of 0.92 for EDRs and 0.84 for ESRs (*SI Appendix*, Fig. S7). We compared the ED score TFBS prediction model to a model that predicts TFBSs based on DNA sequence alone (*Materials and Methods*) and found that the ED score models were more accurate (*P* < 0.05, Wilcoxon rank sum test; *SI Appendix*, Fig. S8). To further validate the EDR/ESR predictions, we calculated the enrichment of SNPs shown to have regulatory activity in HepG2 and K562 MPRA experiments (27) across DHSs, enhancer regions, and EDRs/ESRs defined in the relevant cell line (*Materials and Methods*). We observed a striking increase in the enrichment of regulatory SNPs overlapping EDRs or ESRs, especially those in the top 2% of EDRs/ESRs ranked by their average ED scores (Fig. 2*D*), suggesting that EDRs and ESRs capture meaningful regulatory information.

In total, we identified 420,689 EDRs and 290,532 ESRs across islets, HepG2, and K562 (*SI Appendix*, Fig. S9), with 37% and 23% of EDR and ESR regions located within enhancers active in all three cell lines residing at the same enhancer position, reflecting a partial similarity in the TF regulatory landscape of these cell lines. The identified EDRs were on average 16.5 bp long with an average ED score of 4. The ESRs were 12.4 bp long with an average delta of −2.7. Focusing on islets, within the 9,918 islet enhancers, we identified 74,073 EDRs with an average length of 19.5 bp (average delta of 2.8) and 67,142 ESRs with an average length of 12.2 bp (average delta of −2.3), which we used to aid in the interpretation of candidate causal SNPs of signals associated with T2D and glycemic traits.

### Prediction of the Impact of SNPs on Islet Enhancers.

Having determined that TREDNet captures meaningful regulatory patterns across several biospecimens, we applied TREDNet to predict the effect of 67,226,155 SNPs from the genome aggregation database (28) on islet enhancers by calculating an islet enhancer perturbation score (IEP score; *Materials and Methods*) to guide the identification of candidate causal SNPs at T2D-associated genetic signals. This score weights each SNP based on the probability that the surrounding genomic region is an islet enhancer and the predicted effect of each allele on the islet enhancer probability.

**Fig. 3.**
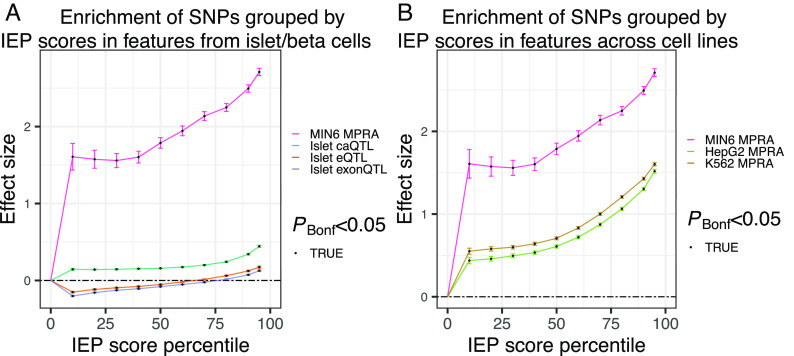
Validation of IEP SNP scores. (*A*) Enrichment and SE (*y* axis) of SNPs grouped by IEP percentile (*x* axis) in islet/beta cell validation data (color). (*B*) Enrichment and SE (*y* axis) of SNPs grouped by IEP percentile (*x* axis) in MPRA signals from MIN6 beta cells, K562, and HepG2 (color).

To validate the IEP scores, we collected SNPs known to affect various islet features including gene expression (expression quantitative trait loci; eQTLs), exon expression (exonQTLs), chromatin accessibility (caQTLs), and MPRA signals from MIN6 beta cells (*Materials and Methods*). We note that of these validation data, all of the MPRA signals are at SNP level resolution while for the other datasets from population genetic studies, the truly causal SNP(s) is not known in many cases due to LD. Next, using progressively strict IEP score percentile cutoffs to select groups of SNPs, we calculated the enrichment of SNPs among the islet features, controlling for the distance of a SNP to the nearest gene and the number of SNPs in LD (*Materials and Methods*). We found a strong and progressive enrichment in SNPs shown to activate transcription in MIN6 MPRA experiments and islet chromatin accessibility (caQTLs; Fig. 3*A*), but not in eQTL or exonQTL signals. These results suggest the IEP score captures meaningful biological effects of SNPs on enhancers and are consistent with studies that report gene expression genetic associations are more strongly enriched in promoter regions than in distal enhancer regions (20, 29).

We sought to evaluate the islet specificity of IEP scores and compared the enrichment of SNPs in islet MPRA data to K562 and HepG2 MPRA data (*Materials and Methods* and Fig. 3*B*). We observed a substantial and progressive enrichment of SNPs in all MPRA data and a stronger enrichment in islet MPRA signals than in K562 or HepG2 (*P* < 0.05, z-test). Together, these results suggest that IEP scores capture a broad range of effects, from islet-specific regulatory programs to regulatory programs common across these biospecimens.

### Candidate Causal SNPs at Genetic Signals Associated with T2D and Glycemic Traits.

As an application of TREDNet, we used TREDNet IEP scores to refine credible sets SNPs for 1,243 non-coding genetic signals associated with T2D (99% credible set; (30)), blood glucose levels (95% credible set; (31)), blood glucose levels after fasting (99% credible set; (32)), and glycated hemoglobin (HbA1c, 95% credible set; (31)). First, we verified that TREDNet IEP scores could distinguish credible set SNPs from SNPs in LD (*Materials and Methods*). We compared IEP scores of credible set SNPs to SNPs in LD with credible set SNPs and found credible set SNPs tended to have larger IEP scores (*SI Appendix*, Fig. S10*A*). Next, we applied two methods to calculate the enrichment of credible set SNPs and LD SNPs i) at increasing IEP score thresholds and ii) using IEP score ranks (i.e., avoiding thresholds and considering all credible set SNPs and LD SNPs at once; *Materials and Methods*). Both techniques showed that larger IEP scores were enriched for credible set SNPs, with no enrichment for SNPs in LD with credible set SNPs (*SI Appendix*, Fig. S10 *B* and *C*). Combined, these results suggest that IEP scores can be used to prioritize candidate causal SNPs for T2D and related glycemic traits.

Having verified that larger TREDNet IEP scores are enriched for credible sets SNPs for T2D and related glycemic traits, we focused on identifying credible set signals for functional studies where a single candidate causal SNP stood out with an IEP score much larger than all other SNPs in the credible set (i.e., pinpointing association signals that showed evidence of a single candidate causal SNP). To identify such signals, we used T2D credible sets from two studies to empirically derive a IEP score cutoff to prioritize candidate causal SNPs: a European ancestry study (6) and a transancestry study (30). We calculated the ratio of the highest IEP score and the second highest IEP score (IEP ratio_1:2_) across all SNPs in the 99% credible set. Because transancestry credible sets have greater power to identify candidate causal SNPs due to different LD patterns, we selected association signals with only one SNP in the credible set from the transancestry analysis. We calculated how many times the IEP ratio_1:2_ correctly nominated the transancestry candidate causal SNP among the multiple SNPs in the credible set identified using the less powered European ancestry study at increasingly stringent IEP ratio_1:2_ thresholds (*Materials and Methods*). At an IEP ratio_1:2_ of >24, we found statistical enrichment (*P* < 0.05, hypergeometric test) of IEP ratio_1:2_ refined SNPs from European T2D signals in candidate causal SNPs from the transancestry fine-mapping analysis (*SI Appendix*, Fig. S11).

**Fig. 4.**
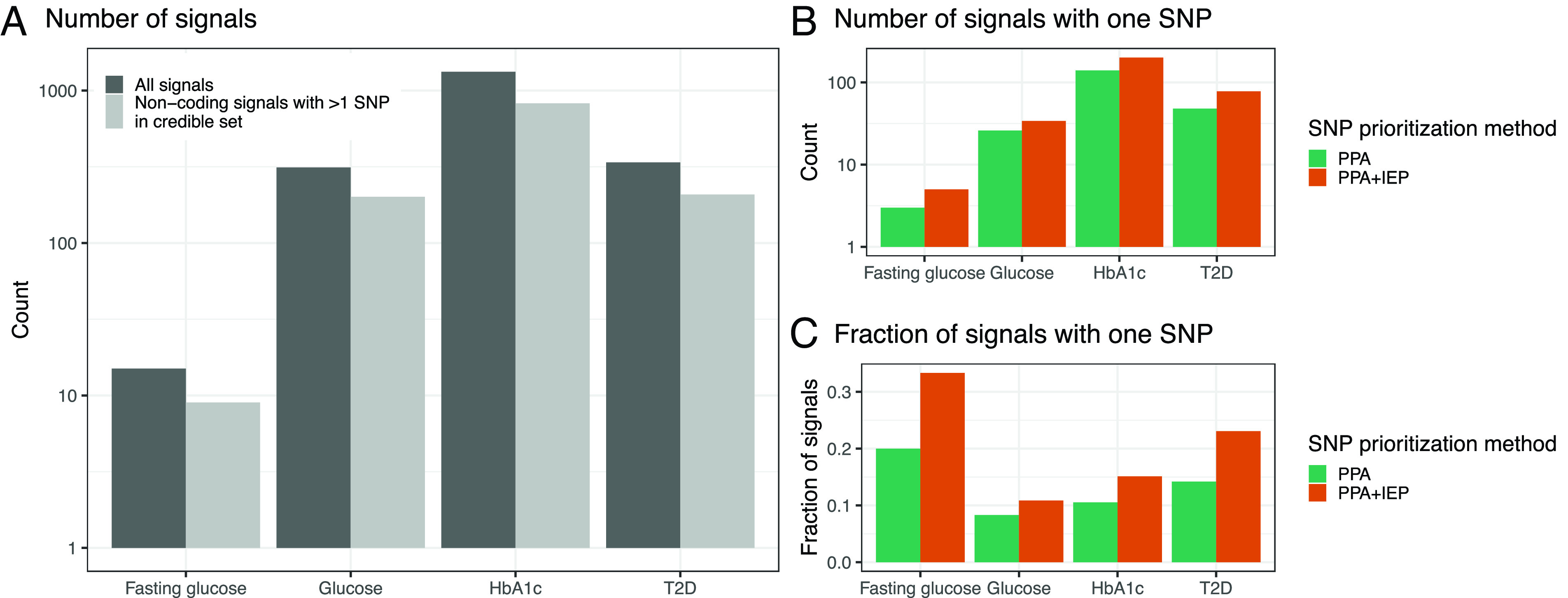
Results of IEP ratio_1:2_ prioritization of credible set SNPs. (*A*) Total number of independent signals (*y* axis) for each disease/trait considered (*x* axis). (*B*) Number of signals with one SNP (*y* axis) in the 95/99% credible set before (green) and after applying the IEP ratio_1:2_ SNP prioritization method (orange) for each disease/trait considered (*x* axis). PPA stands for posterior probability of association. (*C*) Fraction of signals with one SNP (*y* axis) in the 95/99% credible set before (green) and after applying the IEP ratio_1:2_ SNP prioritization method (orange) for each disease/trait considered (*x* axis).

We applied the IEP ratio_1:2_ threshold of >24 to the credible sets for 1,243 noncoding signals associated with T2D (transancestry) and glycemic traits with >1 SNP in the credible set (Fig. 4*A*). For 101 disease/trait signals spanning 94 total SNPs (i.e., some SNPs were associated with more than one phenotype), the IEP ratio_1:2_ was >24, indicating that only one SNP in the credible set had a large IEP score (Fig. 4 *B* and *C* and *SI Appendix*, Table S2). To further validate these predictions, we compared the allelic imbalance of the 94 SNPs prioritized by our method to all other SNPs in the 95/99% credible set using islet chromatin accessibility data (*Materials and Methods*). We found the candidate causal SNPs exhibited greater allele-specific accessibility (*P* = 0.0011, Wilcoxon rank sum test; *SI Appendix*, Fig. S12), as would be expected if these SNPs perturbed the binding of islet regulatory factors. Although no individual SNP exhibited allelic imbalance after multiple hypothesis correction (likely due to the small number of ATAC-seq samples), several of these SNPs have been shown to exhibit allelic activity in reporter assays conducted in MIN6 beta cells: rs7732130 at the 5:76435004 (*ZBED3/PDE8B*; GRCh37 coordinates) T2D signal (33), rs7933438 at the 11:128040810 (*ETS1*) T2D signal (34), and rs4237150 at the 9:4290085 (*GLIS3*) T2D signal (35). We calculated the overlap of the 94 candidate causal SNPs with predicted TFBSs (based on motifs; *SI Appendix*, Table S3 and *Materials and Methods*) and found these SNPs were enriched (false discovery rate [FDR] < 5%) in RFX family-binding sites, consistent with previous studies that report RFX6 as an important islet TF for T2D genetic risk (20, 36). In addition, 73 of the 94 candidate SNPs (79%) overlapped an EDR/ESR, a 16.8-fold enrichment compared to random SNPs (*P* = 1.6 × 10^−22^, binomial test), providing an additional level of evidence at these signals.

To demonstrate the strength of our approach, we i) highlight three of these signals with extensive evidence from previous studies supporting the predicted candidate causal SNP and ii) describe the results of experimental assays that we performed for all 95% credible set SNPs at one glucose-associated genetic signal near *PSMA1*.

The 9:4290085 locus near *GLIS3* is associated with T2D, HbA1c, and glucose. Within the 99% T2D credible set, there are two SNPs: rs4237150 and rs1574285 (*SI Appendix*, Fig. S13). Our model identifies the rs4237150 SNP, with the largest T2D posterior probability of association (PPA = 0.96), as being the likely causal SNP, where the C allele increases the enhancer probability of the region. rs4237150 overlaps an islet ESR, disrupts NR3C1 and ZNF528 binding motifs (*SI Appendix*, Table S3), and lies in an islet stretch/super enhancer (35). The T2D risk allele (C) exhibits increased allele-specific imbalance in islet ChIP-seq and ATAC-seq data (35). Consistent with the TREDNet predictions, the C allele of rs4237150 exhibits increased luciferase reporter activity in MIN6 beta cells (35).

Near *DLK1*, there are genetic associations with T2D, HbA1c, and glucose. Within the HbA1c 95% credible set at 14:91785258, there are two SNPs, rs73347525 and rs8004581 (*SI Appendix*, Fig. S14). Our model predicts rs73347525 to be the candidate causal SNP for the HbA1c signal, with the A allele increasing the enhancer probability of the region. rs73347525 is the only SNP in the T2D 99% credible set at this signal, while for glucose it is one of 32 SNPs, among which our model could not make a confident prediction (there are several SNPs in the region with high IEP scores). The T2D risk allele (A) is associated with increased glucose and HbA1c levels, overlaps a beta cell–specific ATAC-seq peak (37), and is associated with increased expression of *DLK1* in islets (29). *DLK1* expression patterns are highly specific for islets (29), particularly beta cells (38, 39), and *DLK1* exhibits increased expression in T2D beta cells compared to non-T2D (38). We analyzed the EDR/ESR predictions and found rs73347525 overlaps an islet-specific EDR region and most strongly perturbs a motif for ZNF415 (*SI Appendix*, Table S3), where the risk allele (A) results in decreased binding—suggesting that rs73347525 may perturb ZNF415 binding, resulting in increased *DLK1* expression and increased T2D risk.

In addition, near *ZBED3/PDE8B*, there is a strong association with T2D, HbA1c, and glucose. For all three associations, there are three, identical candidate causal SNPs in the 95/99% credible set (*SI Appendix*, Fig. S15). Our model predicts rs7732130 to be the causal SNP, where the G allele is predicted to increase the probability that the region is an enhancer compared to the alternative allele (A). The G allele is associated with increased T2D risk, increased HbA1c, and increased glucose levels. This SNP intersects an islet EDR and the T2D risk allele (G) is predicted to increase ZNF143 and RFX7 binding affinity (*SI Appendix*, Table S3). Consistent with these findings, the T2D risk allele (G) has been shown to increase both in vivo chromatin accessibility in human islets and luciferase reporter activity in MIN6 beta cells (33). Moreover, in human islets, the risk allele (G) is strongly associated with both increased expression of *PDE8B*—a gene with islet-specific expression patterns (20)—and *ZBED3* (29). CRISPR activation and inhibition experiments of the rs7732130 enhancer in human EndoC-βH3 cells, a human pancreatic beta cell line, also show effects of this enhancer region on *PDE8B, ZBED3,* and other transcripts within the region (40). Thus, while the effector gene(s) at this locus is unclear, these data cumulatively support rs7732130 as the most likely causal SNP at this locus.

**Fig. 5.**
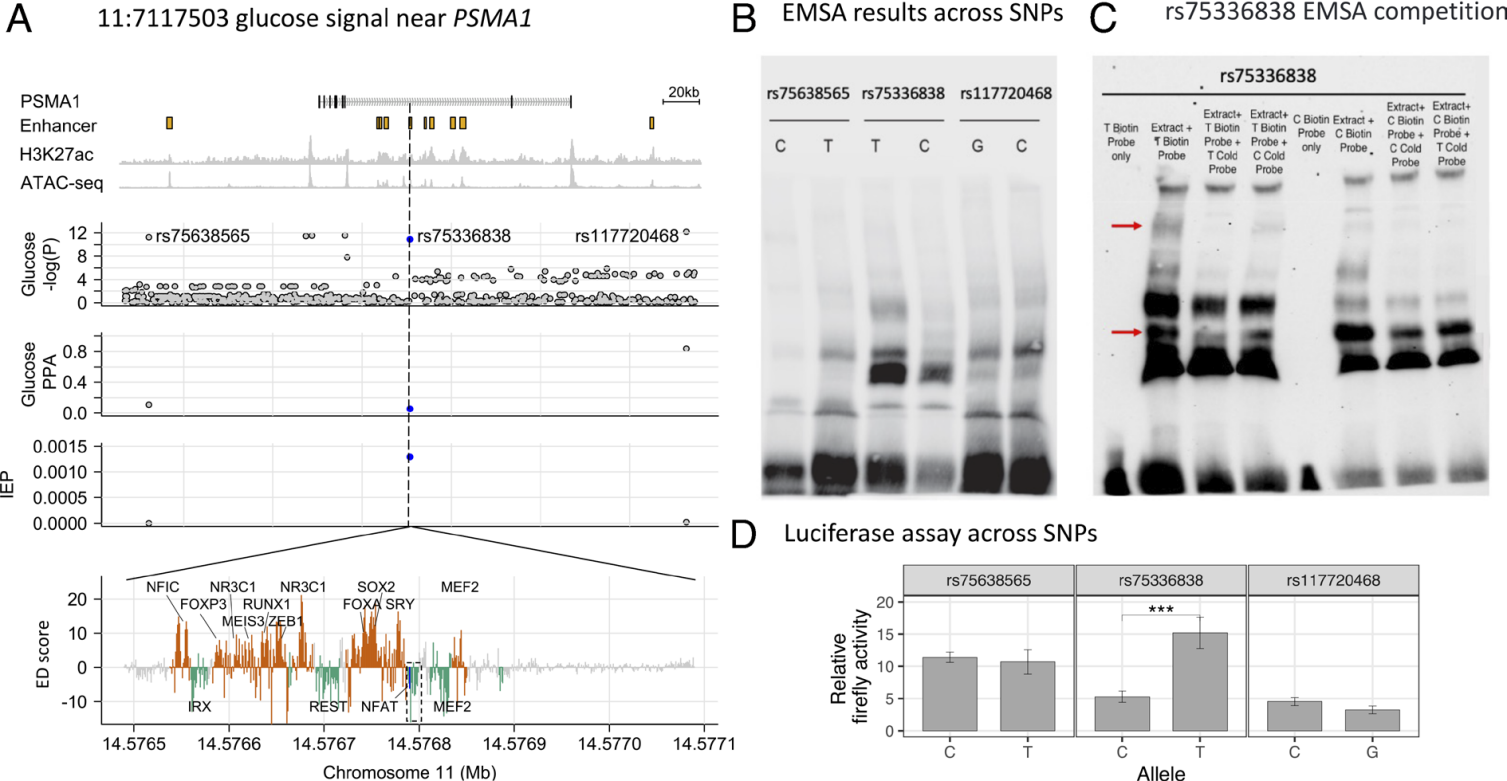
*PSMA1* locus. (*A*) Locus zoom around the 11:7117503 glucose association [Glucose −log_10_(*P*) facet] near *PSMA1*. Top facet shows islet enhancers, called from islet H3K27ac ChIP-seq and ATAC-seq data. rs75336838 (blue) is one of three SNPs in the 95% glucose credible set (PPA facet), has a large IEP score (IEP facet), and occurs in an ESR region (green; EDR regions shown in orange), defined by ED scores from in silico saturated mutagenesis (ED score facet). Dashed box indicates the ESR containing the candidate SNP (blue line). (*B*) Electrophoretic mobility shift assay (EMSA) for all SNPs in the 95% credible set. (*C*) Competition EMSA for rs75336838. Red arrows indicate bands of interest. (*D*) Average luciferase activity across replicates for both alleles of candidate SNPs. Error bars correspond to SE. *** indicates Wilcoxon rank sum test *P* < 0.001.

The 11:7117503 locus near *PSMA1* is associated with HbA1c and glucose. For both traits, among all of the 95% credible sets SNPs, our method prioritized rs75336838 as the likely functional variant (Fig. 5*A*), where the T allele increases the enhancer probability of the region and overlaps an islet ESR region matching several TF-binding motifs (*SI Appendix*, Table S3). To test this prediction biochemically, we assessed the effects of the three SNPs in the glucose 95% credible set on human beta cell nuclear/TF binding using an EMSA (Electrophoretic mobility shift assay) with EndoC-βH3 nuclear extracts. Among the SNPs tested, the candidate causal SNP prioritized by our model, rs75336838, showed the most striking allelic differences in the binding of TFs/complexes contained in human EndoC-βH3 nuclear extracts, where the T allele (associated with increased glucose levels) exhibited a different pattern of binding than the C allele (Fig. 5*B*). To confirm that this variant affected the transcriptional apparatus, we performed an EMSA competition assay focusing on rs75336838. With the T allele probe labeled, we noted two bands showed specific competition by the cold T allele probe (Fig. 5*C*, red arrows). Finally, we tested all three glucose credible set SNPs for luciferase activity in the EndoC-βH1 human pancreatic beta cell line. Only the candidate causal SNP, rs75336838, showed allele-specific activity, with the T allele associated with threefold increased expression, consistent with the EMSA experiments and TREDNet prediction (Fig. 5*D*). While additional work is needed to complete our molecular understanding at this signal, the nomination of a biochemically validated likely causal variant should aid future studies in dissecting the mechanism of action.

## Discussion

DL methods have been applied to the problems of genomics extensively. One of the first DL genomics studies implemented a framework, DeepSEA (11), to learn multiple tissue-specific epigenomic signals (e.g., HMs, TFBSs, DHSs) using CNNs in a multitask learning fashion. The authors later extended the DeepSEA model through the ExPecto (10) model, which doubled the number of epigenomic features and increased the depth and breadth of the CNN. Another method, Basset (12), was trained in similar fashion but on DHS regions only. All of these models used an additional layer to predict molecular effects of mutations in DNA regions; however, these effects were calculated across all learned epigenomic features simultaneously and not tailored to detect altered tissue-specific enhancer activity.

Indeed, to date, few studies have applied DL methods to predict enhancers and prioritize candidate causal variants. For instance, Tan et al. trained an ensemble of recurrent neural networks to predict enhancers using features from physicochemical properties of dinucleotides in enhancer regions (14). Yan et al. developed another method, BiRen, that predicts epigenomic signals from DNA sequences using DeepSEA, and combines these predictions with conservation scores of the input sequence to predict enhancers (13). However, neither of these studies performed extensive post hoc analyses such as in silico mutation analysis or candidate causal variant prioritization. To date, such post hoc analyses have been performed primarily using methods that predict epigenomic features directly (10–12, 41) or non-DL enhancer prediction methods, like SVM (15, 42).

In this study, we developed TREDNet, a model that utilizes epigenomic signal predictions from DNA sequence to model enhancers. Compared to other enhancer prediction methods (13–15), TREDNet consistently improves enhancer detection (Fig. 2*A*). We found that TREDNet’s enhanced enhancer modeling translates directly to more accurate modeling of signals from in vitro MPRA experiments of enhancer regions, compared to previous methods (Fig. 2*B* and *SI Appendix*, Fig. S4). We applied TREDNet to i) better understand the overall epigenomic regulatory structure of islet enhancers and ii) refine credible sets from genetic association studies.

By computing the effects of all possible mutations in islet enhancers, we found that we could accurately predict TFBSs from ChIP-seq experiments and TF footprints derived from ATAC-seq data (*SI Appendix*, Fig. S7) as short genomic regions (average length 13.7 bp) that greatly alter the overall enhancer probability of a 2 kb genomic region, termed EDRs and ESRs. Previous DL methods for TFBS prediction, such as BPNet (43), have used saliency maps to detect TF motifs [e.g., DeepLIFT (44), TF-MoDISco (45)] and applied the detected motifs to predict TF binding. However, to predict binding sites of a specific TF, such methods require experimental data of the specific TF in question to train the model. By contrast, TREDNet’s peak/dip detection module enables the detection of putative TFBSs of arbitrary TFs throughout the genome that may impact the enhancer function of the surrounding genomic regions. For example, of the 141,215 EDRs/ESRs detected in islets, 28,081 (20%) do not overlap a known TFBS. We hypothesize that these sites correspond to unmeasured TFBSs or to TFBSs that become bound and active in response to specific stimuli (e.g., glucose stimulation, stressors like inflammation) and thus are not detected in studies that identify TF binding under baseline conditions. Future studies will be required to test this hypothesis.

Importantly, we used TREDNet predictions to nominate candidate causal mutations at several signals associated with T2D and glycemic traits. By design our IEP ratio_1:2_ technique implicitly prioritizes signals where a single candidate causal variant stands out from among all other variants. To facilitate other prioritization schemes, we distribute precomputed enhancer probability and IEP scores for 67,226,155 SNPs along with the underlying TREDNet models (*SI Appendix*, *Supplementary Materials and Methods*). By applying the IEP ratio_1:2_ technique, we were able to prioritize a single candidate causal variant in the 95/99% credible set from statistical fine-mapping at 101 signals (94 unique SNPs; *SI Appendix*, Table S2). These predictions include multiple SNPs for which functional allelic effects have been detected previously in vitro in islet beta cells (33–35) and one for which we provide biochemical validation. We anticipate that further validation experiments of candidate causal variants nominated in this study will lead to additional insights into the molecular genetic basis of T2D and T2D-related traits. In addition, extending the computational techniques presented in this study to inform fine-mapping of genetic associations for islet-relevant diseases or traits by using IEP scores as priors represents a promising area of further research with the potential to further refine candidate causal variants. Finally, we note that, in addition to common variants, using TREDNet and similar techniques to model and prioritize rare variants in the context of T2D and other diseases will be an important avenue for future studies.

## Materials and Methods

A detailed description of computational and experimental analyses is provided in the *SI Appendix*, *Supplementary Materials and Methods*. Briefly, we developed a model, TREDNet, to predict enhancers in a two-phase process, implemented in keras v2.1.2. We trained phase one to predict DHSs, TF ChIP-seq peaks, and histone mark ChIP-seq peaks using data from the ENCODE (8) and NIH Roadmap (9) studies. We trained phase two to predict pancreatic islet, HepG2, and K562 enhancers, defined as 2 kb regions centered on overlaps between H3K27ac ChIP-seq peaks and chromatin accessibility peaks in each tissue/cell line. We applied TREDNet to i) perform in silico saturated mutagenesis experiments, ii) predict TFBSs from saturated mutagenesis profiles, and iii) predict the effects of SNPs on islet enhancers. Using these data, we prioritized candidate causal SNPs in statistical fine-mapping results for T2D (30), blood glucose levels (31), blood glucose levels after fasting (32), and glycated hemoglobin (31). At one signal associated with blood glucose levels (near *PSMA1*), we performed EMSAs and luciferase assays in pancreatic beta cell lines to confirm the predicted allelic effects among all SNPs in the 95% credible set.

## Supplementary Material

Appendix 01 (PDF)

Dataset S01 (DOCX)

## Data Availability

Genomic data have been deposited in zenodo (https://doi.org/10.5281/zenodo.8161621) (46).
