# Supplementary material for: Modeling islet enhancers using deep learning identifies candidate causal variants at loci associated with T2D and glycemic traits
Source: Proc Natl Acad Sci U S A. 2023 Aug 21;120(35):e2206612120. doi: 10.1073/pnas.2206612120 (PMC10469333; doi:10.1073/pnas.2206612120)
Supplement: Supplementary file 1 — Appendix 01 (PDF) [file pnas.2206612120.sapp.pdf]

PNAS

Supplementary Information for

Modeling islet enhancers using deep learning identifies candidate causal variants at loci associated with T2D and glycemic traits.

Sanjarbek Hudaiberdiev[1,A], D. Leland Taylor[2,A], Wei Song[1,A], Narisu Narisu[2,A], Redwan M. Bhuiyan[3,4], Henry J. Taylor[2,5], Xuming Tang[6,7], Tingfen Yan[2], Amy J. Swift[2], Lori L. Bonnycastle[2], DIAMANTE Consortium, Shuibing Chen[6,7], Michael L. Stitzel[3,4,8], Michael R. Erdos[2,B], Ivan Ovcharenko[1,B], Francis S. Collins[2,B]

1. Computational Biology Branch, National Center for Biotechnology Information, National Library of Medicine, National Institutes of Health, Bethesda, MD 20892, USA
2. Center for Precision Health Research, National Human Genome Research Institute, National Institutes of Health, Bethesda, MD 20892, USA
3. The Jackson Laboratory for Genomic Medicine, Farmington, CT 06032, USA
4. Department of Genetics and Genome Sciences, University of Connecticut, Farmington, CT 06032, USA
5. British Heart Foundation Cardiovascular Epidemiology Unit, Department of Public Health and Primary Care, University of Cambridge, Cambridge CB1 8RN, UK
6. Department of Surgery, Weill Cornell Medicine, New York, NY 10065, USA
7. Center for Genomic Health, Weill Cornell Medicine, New York, NY 10065, USA
8. Institute of Systems Genomics, University of Connecticut, Farmington, CT 06032, USA

A. These authors contributed equally to this work.
B. To whom correspondence may be addressed. Email: mikee@mail.nih.gov, ovcharei@ncbi.nlm.nih.gov, and francis.collins@nih.gov.

This PDF file includes:

Supplementary text
Supplementary references
Figures S1 to S15
Tables S1 to S8

Supplementary Materials and Methods

Genome annotations

ENCODE and NIH Roadmap genomic and epigenomic profiles

We used previously published DNase I hypersensitive sites (DHSs), ChIP-seq peaks of histone marks (HM), and ChIP-seq peaks of transcription factor (TF) binding from the ENCODE (1) and NIH Roadmap (2) studies (1,924 features in total).

Islet genomic and epigenomic profiles

We used previously published ATAC-seq data from 33 islets, consisting of 64,129 peaks (3). As described in Viñuela et al. (3), since these islets originate from multiple studies, reads were downsampled to the minimum read depth across samples (27,994,993 reads) and merged across studies. Peaks were called across all samples (via the merged bed file) as well as within samples. The final 64,129 ATAC-seq peak calls were those peaks from the merged bed file that occurred in >17 of the individual samples ATAC-seq peak calls.

We reprocessed previously published H3K27ac ChIP-seq data from two islets (4). Briefly, in order to avoid bias from one specific sample, we downsampled the number of reads from each sample to the minimum read depth across samples (26,369,910 reads) and merged reads from both samples. We performed a similar process for the input controls for each sample, downsampling to 23,702,108 reads per sample before merging reads across samples. Next, we aligned reads and identified peaks using the ENCODE ChIP-seq processing pipeline (https://github.com/ENCODE-DCC/chip-seq-pipeline2, v1.2.0) with default parameters. We excluded the peaks overlapping Duke blacklisted regions (UCSC browser tables wgEncodeDacMapabilityConsensusExcludable and wgEncodeDacMapabilityConsensusExcludable), resulting in 87,007 H3K27ac peaks.

Enhancer definitions

We identified 9,918 islet enhancers by selecting ATAC-seq peaks that overlapped an islet H3K27ac ChIP-seq peak (≥1bp) and expanding 1kb up and down the genome from the middle position of the ATAC-seq peak. We considered the entire 2kb region, centered on the ATAC-seq peak, as an enhancer. We removed promoter regions, defined as 1.5kb upstream and 0.5kb downstream (2kb in total) of the transcription start site of known genes from the UCSC genome browser (ftp://hgdownload.soe.ucsc.edu/goldenPath/hg19/database/knownGene.txt.gz).

To define enhancers in HepG2 and K562, we used the ENCODE data and performed the same procedure, using ATAC-seq peaks for HepG2 (n=279,739; ENCSR042AWH) and K562 (n=269,800; ENCSR868FGK) from ENCODE. We identified 21,162 HepG2 and 25,357 K562 enhancers in total.

Two phase deep learning model to predict enhancers

We developed a two phase deep learning (DL) classifier, TREDNet, based on convolutional neural networks (CNNs; implemented in keras v2.1.2 and tensorflow-GPU v1.4.1) to predict

enhancers from DNA sequence. In the first phase, we used a model with six convolutional layers (~143 million trainable parameters; Table S4) to predict 1,924 different genomic and epigenomic features simultaneously for a 2kb genomic region. These features included DHSs, TF ChIP-seq peaks, and histone mark ChIP-seq peaks from the ENCODE (1) and NIH Roadmap (2) studies. We tiled the entire human genome using a sliding window of length 2kb and the step length of 200bp. We selected those segments that overlapped at least one of the 1,924 epigenomic features and trained the DL model using these segments. For training, we fit the model on all autosomes, except for chromosomes 8 and 9. During training, we used signals on chromosome 7 as a validation set. We used signals on chromosomes 8 and 9 for testing the final model, evaluating both the area under the receiver operating characteristic curve (auROC) and area under the precision recall curve (auPRC; Fig. S1). Due to the cell/tissue type-specific nature of the features, the class distribution (positive/negative) in the training set was imbalanced across the features, with only 2% of the dataset being positive cases on average. To evaluate the optimal sequence window size, we randomly selected 10% of the training data and re-fit the model using different window sizes ranging from 800bp-4kb and the same training/testing strategy (i.e., chromosome 7 as a validation set during training and chromosomes 8 and 9 for testing the final model). We evaluated model performance using auROC and auPRC as metrics, testing for improved model performance (defined as P-value<0.05) across progressively larger window sizes using the Wilcoxon rank sum test (Fig. S2). We found that the models improved at each larger window size up to 2kb, after which there was no statistical improvement. Using the 2kb model, we compared the TREDNet phase one model to previously published, similar epigenomic feature prediction models—ExPecto (5), DeepSEA (6), and Basset (7)—on the data used for TREDNet with the same testing strategy (i.e., testing performance on chromosomes 8 and 9; Fig. S1).

For the second phase (enhancer prediction), we fit three smaller models to predict pancreatic islet, HepG2, and K562 enhancers (one for each cell/tissue type) from the output of the first model (a vector of the 1,924 epigenomic predictions for 2kb sequence segments). The second model consisted of two convolutional layers resulting in ~12 million trainable parameters (Table S5). For each biospecimen, we treated the enhancer regions as the positive set (encoded as 1); for the negative set (encoded as 0), we randomly sampled 10x of the number of enhancers from accessible chromatin regions (i.e., DHSs) across all biospecimens in NIH Roadmap, excluding the enhancer regions of the target biospecimen. For training, we adopted a similar strategy as phase one, using chromosome 7 to validate during training and withholding chromosomes 8 and 9 to evaluate the final model. We tested the model's performance (auROC and auPRC) using enhancers from chromosomes 8 and 9 withheld from training (Fig. 2A). To benchmark the TREDNet phase two model, we tested previously described enhancer prediction models—BiRen (8), Tan et al. (9), and SVM (10)—on the data used for TREDNet with the same testing strategy (i.e., testing performance on chromosomes 8 and 9). Since SVM did not distribute pre-trained models, we trained SVM using the same data (i.e., enhancer definitions) and training strategy as for TREDNet. For BiRen and Tan et al., we used the pre-trained models which were not cell type specific. For Tan et al., the method produces a binary output for each input region based on the scores generated by five different models. To generate auROC and auPRC values, we used the scores generated by each of the five Tan et al. models and picked the best performing one.

Finally, to evaluate the utility of the two phase TREDNet model structure as opposed to a single phase model, we (i) combined the phase two enhancer region training data with the phase one training data and (ii) fit a single model to predict both types of signals using the phase one model architecture (Fig. S3; training and testing strategy same as used previously with chromosomes 7, 8, and 9). We found that for epigenomic signals (i.e., transcription factors, histone modifications, DNase I hypersensitivity sites), the two phase TREDNet model performed better than the single phase model using auROC and auPRC as metrics (*P*-value<0.05, Wilcoxon rank sum test). We hypothesize that this difference may be due to the increased complexity of the single phase model that must predict both epigenomic signals and enhancer regions. For the task of enhancer prediction, the TREDNet model also outperformed the single phase model, although not at a statistically significant level (*P*-value>0.05, Wilcoxon rank sum test)—possibly due to the small sample size (n=3; i.e., enhancers from islets, HepG2, and K562). As a last comparison, we considered training speed and found that for the end goal of predicting enhancers, the two phase TREDNet model has an advantage over a single phase model in that it can be trained to predict enhancers much faster than the single phase model.

Analysis of in vitro enhancer mutagenesis experiments

We validated TREDNet enhancer probability scores using data from three massively parallel reporter assay (MPRA) studies (11–13), described below. For all datasets, we calculated computational scores based on the DNA sequence tested in the MPRA using TREDNet and the other enhancer modeling methods considered in this study: BiRen, Tan et al., and SVM (trained using the same enhancer dataset as used for TREDNet as described in “Two phase deep learning model to predict enhancers”). For TREDNet, SVM, and BiRen, we expanded each input genomic DNA segment to 2kb; for Tan et al., we extended the input DNA segments to 200bp, as required by the method. We compared each of the computational scores for each study to the MPRA signal by calculating Spearman's rank-order correlation and root mean squared error (RMSE; Fig. 2B,S4). To make RMSE comparable across models and datasets, we standardized all values prior to calculating RMSE. Since the Tan et al. method uses five models with varying architectures, we selected the best model for each dataset and comparison metric (i.e., largest value for Spearman's rank-order correlation and smallest value for RMSE.

Kheradpour et al. (11) performed MPRA experiments in HepG2 and K562 that quantified the capacity of DNA segments of predicted TF binding sites (TFBSs) in enhancers to induce gene expression. Using the normalized expression scores distributed by the authors, we filtered for sequences with a detectable impact on gene expression compared to null, scrambled sequences (FDR<5%, Benjamini-Hochberg procedure (14)). We applied the biologically relevant TREDNet model (i.e., the HepG2 model for HepG2, the K562 model for K562) to predict the enhancer probabilities of each DNA segment and compared these predictions to the MPRA signals. We repeated this process for BiRen, Tan et al., and SVM.

Kwasnieski et al. (13) conducted MPRA experiments in K562 that measured the capacity of DNA sequences in K562 enhancer regions (“enhancer” and “weak enhancer” chromatin states) to induce gene expression. We used the K562 TREDNet model to predict the enhancer probabilities of

each DNA segment and compared these predictions to the MPRA signals, using the normalized expression scores provided by the authors. We repeated this process for BiRen, Tan et al., and SVM.

Finally, Kircher et al. (12) performed saturated mutagenesis experiments targeting several regulatory elements associated with disease. We used data from this study from enhancer regions that tested for effects of mutations in regions near *ZFAND3* and *TCF7L2* in MIN6 and *SORT1* in HepG2. Using the $\log_2$ allelic MPRA expression effect estimates distributed by the authors, we filtered for alleles with >=10 unique barcode tags (as done by the authors) and for alleles that showed a statistical difference in the MPRA expression read out (FDR<5%, Benjamini-Hochberg procedure (14)). We applied the biologically relevant TREDNet model (i.e., the islet model for MIN6, the HepG2 model for HepG2) calculated the $\log_2$ fold change of enhancer probability scores for the alternate allele compared to the reference allele, comparing these predictions to the MPRA signals. We repeated this process for Tan et al. and SVM. For BiRen, because the method takes as input coordinates of genomic regions, not DNA sequences, we used the enhancer probabilities of the overall regions.

Calculation of enhancer damage scores from in silico saturated mutagenesis

For each biospecimen, we performed in silico saturated mutagenesis of enhancer regions to evaluate the effects of mutations on the overall enhancer probability score. For each 2kb enhancer region (see “Enhancer definitions”), we calculated an enhancer damage (ED) score iteratively for each nucleotide position against the GRCh37 reference sequence (i.e., we mutate each nucleotide to all possible mutations but keep the remaining 1,999 nucleotide sequence the same as the reference):

$$\sum(e_{reference} - e_{alternate})/3$$

where the *e* term represents the probability that the 2kb sequence is an enhancer, *reference* indicates the GRCh37 reference nucleotide, and *alternate* indicates a non-reference nucleotide. This ED score, generated for each bp in the 2kb enhancer region, predicts the effects of mutations at a specific base on the overall enhancer probability of the region, such that a positive score indicates a negative change in the enhancer probability (enhancer damaging) and a negative score indicates a positive change in the enhancer probability (enhancer strengthening).

Identification and analysis of transcription factor binding sites (TFBSs)

For HepG2 and K562, we used TF ChIP-seq data spanning 77 TFs in HepG2 and 150 TFs in K562 (1). For each TF, in order to extract the TFBS position, we used HOMER v4.11 (15) to scan motifs found in the database packaged with HOMER (http://homer.ucsd.edu/homer/motif/motifDatabase.html), producing a list of predicted TFBSs for each motif in the database. We ranked the motifs in decreasing order of their enrichment *P*-value and kept the TF of the most enriched motif.

Because islets do not have as comprehensive TF ChIP-seq profiles as HepG2 and K562, we used predicted TFBSs derived from ATAC-seq footprints described previously (16). Briefly, for two islet ATAC-seq samples, Varshney et al. scanned for potential transcription factor binding sites

(TFBSs) in a haplotype-aware manner using the "find individual motif occurrences" (FIMO) tool (17) with position weight matrices (PWMs) from a previously described database (18) consisting of PWMs from ENCODE (19), JASPAR (20), and Jolma et al. (21). Next, Varshney et al. used CENTIPEDE (22) to call footprints in the islets ATAC-seq data, considering a given motif occurrence bound if both the CENTIPEDE posterior probability was ≥0.99 and the motif's coordinates were fully contained within an ATAC-seq peak.

For islets, because many of the predicted TFs share similar motif patterns and result in overlapping predicted TFBSs, we reduced redundancy by aggregating islet TF footprints with similar binding motifs. For each TF, we calculated the average ED score across all nucleotides within the predicted binding sites. For the TFs with a positive average ED score across all predicted TFBSs (690 TFs), we selected 156 TFs where the average lower boundary of 95% confidence interval (CI) the binding region was greater than the upper boundary of the CI of the flanking region (defined as 10bp on each side of the TFBS). For the TFs with a negative average ED score across all predicted TFBSs (49 TFs), we selected 10 TFs where the ED scores within the CI were all negative. Using these 166 TFs, we iteratively merged TFs if >40% of their predicted binding sites overlapped and the overlapping regions were greater than half of either binding site. In total, this procedure resulted in 100 non-redundant TFs groups for islets, which we used to analyze ED scores in islet enhancers.

We used TFBSs in islets, K562, and HepG2 to evaluate if ED scores mark TFBSs by comparing the absolute value of ED scores within TFBSs to 20bp regions immediately flanking each TFBS as well as randomly sampled enhancer regions. To generate random ED scores, we shuffled the TFBSs within enhancer regions for each biospecimen 10 times and recorded the absolute value of ED scores within these regions.

In addition, to compare ED scores to evolutionary conservation profiles at TFBSs, we calculated the information content (IC) at each position of PWMs for each TF. For evolutionary conservation profiles, we used phyloP scores generated from 46 vertebrate species (23, 24). Across all TF PWMs for each biospecimen, we calculated the correlation (Spearman's rho) between the IC of each PWM position and delta/phyloP scores.

Detection of enhancer damaging regions and enhancer strengthening regions

For each biospecimen, we trained two DL models, one to predict enhancer damaging regions (EDRs) and one to predict enhancer strengthening regions (ESRs) within enhancers from ED score enhancer profiles (six models total across all three cell lines).

To train the EDR/ESR classifiers for each biospecimen, we annotated nucleotides within enhancers with 1 (positive set) if the nucleotide overlapped a TFBS and otherwise 0 (control set). We subsequently excluded from the control set (i.e., enhancer nucleotides annotated as 0) genomic regions (i) between any two TFBSs in an enhancer (even if these TFBSs are on the opposite ends of the enhancer), (ii) within 10bp of a TFBS, (iii) within 20bp of an enhancer boundary, and (iv) in an enhancer of less than 50bp. For HepG2 and K562, we used all TFBSs, described in "Identification and analysis of transcription factor binding sites". For islets, we used the TFBSs from the 166 TFBSs derived from ATAC-seq footprints, described in "Identification and analysis of transcription factor binding sites".

For each enhancer nucleotide, we derived a series of features from ED score predictions across various window sizes and used these features to predict the location of TFBSs within the enhancer sequence (i.e., the positive or control status of each nucleotide within the enhancer). For each nucleotide, we scanned a series of windows from 10bp in length to 1bp in length. For each window length >7bp, we defined a core region as the 6bp in the center of the window and calculated the following metrics: (i) the average ED score of nucleotides within the window, (ii) the maximum ED score of nucleotides within the window, (iii) the fraction of nucleotides within the window with a positive ED score, and (iv) the fraction of nucleotides within the core region with a positive ED score. For windows of length <6bp, we repeated the same procedure, but using a core region equal to the window size. For each window length, we iteratively scanned around the target nucleotide such that the target nucleotide occupied every position within the window (i.e., we incremented the window position by one base pair for each iteration resulting in 10 sliding windows for a window size of 10bp). For a single nucleotide, the result of this procedure was a series of four metrics defined across 55 windows (of size 10bp to 1bp). We concatenated these metrics together across all windows to generate a vector of 220 values for each nucleotide, representing a comprehensive description of a local mutational impact of each nucleotide on an enhancer. After iteratively performing this procedure across all nucleotides within enhancers, we then fit a two layer CNN (implemented in keras v2.0.8; architecture described in Table S6) to predict the TFBS annotation status (0 or 1) from the 220 values calculated for each nucleotide. During training, we randomly sampled 20% of the input data as a validation set and excluded chromosomes 8 and 9 entirely. We used signals on chromosomes 8 and 9 for testing the final model, evaluating both the auROC and auPRC (Fig. S7).

To train the EDR classifier, we selected for TFBSs where the average ED score was positive. To train the ESR classifier, we performed the same procedure, except we selected for TFBSs where the average ED score was negative.

Both the EDR and ESR models were highly accurate (Fig. S7) and resulted in a score for each nucleotide within an enhancer representing the likelihood that the nucleotide overlaps a TFBS. We used these scores to label regions as EDRs and ESRs. For the EDR models, we used the peak model for each biospecimen and labeled any span of nucleotides (one after the other) of length >=3 with a likelihood score >=0.312 as an EDR. We repeated the same procedure with the ESR models, calling regions with >=3 consecutive predicted negative EDR nucleotides and a likelihood score >=0.152 as ESRs.

We compared the TREDNet TFBS prediction model from enhancer damage scores to a model that predicted TFBSs directly from DNA sequence. For the DNA sequence TFBS prediction, we fit a single multi-task model to predict the TFBS annotation status (0 or 1) used in the EDR/ESR models from one-hot encoded DNA sequence of the 2kb region centered on the TFBSs (model architecture the same as TREDNet phase one). We fit a separate model for each biospecimen (i.e., islet, HepG2, K562), randomly sampling 20% of the TFBSs, and compared the resulting model to the TREDNet TFBS prediction model using the auROC and auPRC as metrics with the same training validation and test scheme as used in the ED score model (Fig. S8).

To validate the EDR and ESR predictions as regulatory sites within enhancer sequences, we compared the density of SNPs reported to have an allelic effect on transcription in K562 and HepG2

MPRA experiments (25) in DHSs, enhancer regions, and EDR/ESR regions ranked by their average ED score. For comparisons, we calculated the number of MPRA validated SNPs in each genomic region divided by the total length of the region.

Calculation and validation of islet enhancer perturbation scores

We generated a catalog of predicted effects of 67,226,155 SNPs on islet enhancers using the genome Aggregation Database (gnomAD) v3.0 (26), including SNPs with a minor allele frequency (MAF) >=0.0001. Since gnomAD v3.0 uses GRCh38 coordinates, we lifted these coordinates over to GRCh37 to match the data used to train the model. Across all SNPs, we calculated islet enhancer perturbation (IEP) scores using a two phase procedure. First for each SNP, we calculated the probability, *e*, of either allele falling in an enhancer, given 2kb of the flanking reference sequence (GRCh37) surrounding the SNP. Next, we generated IEP scores:

$$max(e_{reference}, e_{alternate}) \; * \; abs(e_{reference} - e_{alternate})$$

where the *e* term represents the probability of an allele residing in an islet enhancer. For enrichment calculations and subsequent binning, we calculated the percentile rank of IEP scores.

To validate IEP scores at the genome-wide level, we used previously published islet genetic studies spanning gene/exon expression (3), chromatin accessibility (27), and MPRA data generated from the MIN6 mouse pancreatic islet beta cell line (28). From these data, we generated genome annotations that were subsequently used for enrichment calculations. For the genetic association data (eQTLs, exonQTLs, and caQTLs), we selected all QTLs and marked the genomic location of all SNPs in LD (r^2>0.8) with the lead SNP (minimum *P*-value at the locus). For the MIN6 MPRA data, we marked the genomic location of SNPs reported to induce activity in either the unstimulated (baseline) or stimulated (endoplasmic reticulum stress) conditions. We did not LD expand the MPRA SNPs because the MPRA data has SNP resolution since the alleles of single SNP were tested in a reporter construct, thereby breaking the LD structure that exists naturally in the human genome. Using these annotations, we calculated the enrichment of SNPs across progressive IEP percentile cutoffs using GARFIELD v2.0 (29), a logistic regression method that controls for the distance of each SNP to the nearest gene and the number of SNPs in LD. For these enrichments, we used the ~24 million SNPs from the UK10K project and their LD estimates included in the GARFIELD package. Rather than using binarized *P*-value thresholds from a genetic association study to define the dependent variable for the logistic regression, we used IEP percentile cutoffs. Across all enrichment tests, we used the Bonferroni procedure to control for the number of tests performed.

For islet specificity comparisons, we calculated the enrichment of previously published MPRA data from the K562 and HepG2 cell lines (25). We compared the enrichment coefficients of the MIN6 MPRA data to K562 and HepG2 using a z-test as described in Paternoster et al. (30).

Finally, in addition to the genome-wide enrichments, we also compared the enrichment of credible set SNPs to SNPs in LD across IEP scores. We used credible set SNPs for T2D, fasting blood glucose levels, blood glucose levels, and glycated hemoglobin (HbA1c) levels as described in

the "Refinement of credible sets for T2D and glycemic traits" section. For each credible set SNP, we selected the SNPs in LD (r^2>0.8) based on the 1000 genomes phase 3 (v5) European reference panel (31). We calculated the enrichment of both credible set SNPs and LD SNPs across increasing IEP percentile cutoffs by performing a hypergeometric test (phyper function in R), controlling for the number of tests using the Bonferroni procedure. We also calculated the enrichment of credible set SNPs and LD SNPs across SNPs ranked by IEP scores using fGSEA v1.25.1 (32).

Refinement of credible sets for T2D and glycemic traits

To empirically define a threshold for prioritizing candidate causal SNPs using IEP scores, we used 99% credible sets from two T2D fine-mapping genetic studies (uniform priors): a European ancestry study (33) and a trans-ancestry study (34). To focus on likely distal regulatory signals, we excluded signals where >=1 SNP fell in a coding region from both datasets. For each T2D-associated signal in each study, we calculated the ratio of the IEP score of the largest IEP score to the second largest IEP score (IEP $ratio_{1:2}$). Next, we treated the trans-ancestry study as a "truth set", since trans-ancestry studies have greater power to fine-map due to different LD patterns across ancestries, and asked how often we could nominate the trans-ancestry candidate causal SNP using progressive IEP $ratio_{1:2}$ cutoffs in the European ancestry study. To make the trans-ancestry "truth set", we selected the 11 signals with one SNP in the 99% credible set that were not fine-mapped to a single candidate causal SNP in the European ancestry study. We selected the 179 from the European ancestry study with >1 SNP in the 99% credible set and performed a hypergeometric test (phyper function in R) across progressive IEP $ratio_{1:2}$ thresholds. We selected the minimum IEP $ratio_{1:2}$ cutoff with P<0.05, corresponding to an IEP $ratio_{1:2}$ of 24, and applied the cutoff to the trans-ancestry credible sets to identify additional signals with a signal candidate causal SNP.

We applied the IEP $ratio_{1:2}$ of 24, to 99% credible sets for T2D (34), 99% credible sets for blood glucose levels after fasting (35), 95% credible sets for blood glucose levels (36), and 95% credible sets for glycated hemoglobin (HbA1c) levels (36). We note that the credible sets for blood glucose levels after fasting were obtained from the Open Targets genetics platform (https://genetics.opentargets.org).

Allelic imbalance analysis

For allelic imbalance analysis, we collected 24 islet ATAC-seq samples from non-diabetic donors for which SNP genotypes were also available: 1 sample from Varshney et al. (16), 10 samples from Rai et al. (37), and 13 samples from Khetan et al. (27). We note that in instances where the same sample was sequenced more than once across studies, we chose one sample randomly. We tested for allelic imbalance at all SNPs in the 95/99% credible sets for T2D and glycemic traits where our model predicts a single candidate causal SNP that previously had >1 candidate causal SNP at each association signal (Table S2). We followed the computational procedure outlined in Greenwald et al. (38). Briefly, we mapped reads using bwa v0.7.17-r1194-dirty (39) and filtered reads with low mapping quality ("-q 30 -M"). We then used WASP v0.3.4 (40) to remove duplicate reads and correct for reference mapping bias. Using a binomial test to assess imbalance on a per sample basis, we

tested for allelic imbalance at SNPs that had at least two heterozygotes with two or more reads covering each allele. Finally, we calculated z-scores and used Stouffer's method (41) to calculate a combined z-score and *P*-value across samples, weighting the z-scores by the sequencing depth of each sample (42). We controlled for the number of tests using the Benjamini-Hochberg procedure (14).

TFBSs enrichment of candidate causal SNPs

To calculate the enrichment of the candidate causal SNPs in TFBSs, we calculated the fraction of SNPs that overlap TFBSs predicted from ATAC-seq footprints (see "Identification and analysis of transcription factor binding sites") using both the candidate causal set and a 10-fold control set, derived from islet DHS regions. We computed the fold-enrichment, calculated *P*-values using Fisher's exact test, and controlled for the number of tests using the Benjamini-Hochberg procedure (14).

Calculation of the effects of candidate causal SNPs on TFBS motifs

To calculate the allelic effects of candidate causal SNPs on binding motifs, we used TFBS motifs from ENCODE (19), JASPAR (20), and HOMER (http://homer.ucsd.edu/homer/motif/motifDatabase.html). We ran FIMO (packaged with MEME v4.9.0) with a *P*-value threshold of 0.01, keeping motifs that had hits for the sequences of both alleles. The remaining motifs were sorted in the decreasing order of the value $|\log(P\text{-value}_{ref}) - \log(P\text{-value}_{alt}|)$.

Electrophoresis mobility shift assay (EMSA) experiments

For EMSA experiments, we designed 21bp biotin end-labeled complementary oligonucleotides with each SNP allele tested centered at the 11th position of the oligo (Integrated DNA Technologies; Table S7). Each forward and reverse oligo for the biotinylated probes were biotinylated at their 5' ends. We annealed complementary oligos to create double-stranded probes for each tested sequence. Using the NE-PER Extraction Kit (Thermo Scientific), we prepared nuclear extract from human EndoC-βH3 cells in the non-proliferating, excised state (43) and completed EMSAs using the LightShift Chemiluminescent EMSA kit (Thermo Scientific) according to the manufacturer's instructions. Each binding reaction contained 1X binding buffer, 1 μg poly dI-dC, 4 μg EndoC-βH3 nuclear extract, and 200 fmol of biotinylated double-stranded probe. For competition reactions, 20- or 40-fold excess of unlabeled probe for each allele was added to the reaction mix and pre-incubated at 25°C for 15 minutes prior to addition of the labeled probe. We incubated reactions at 25°C for 25 minutes, after which we resolved DNA-protein complexes on a 6% DNA retardation gel (Invitrogen) and detected them by chemiluminescence after transfer and UV crosslinking to a nitrocellulose membrane.

Luciferase experiments

We synthesized 701bp DNA fragments around the identified SNPs (GenScript Biotech Corporation) and cloned the fragments into a pGL3-Promoter vector with XhoI and BglII restriction

sites. We co-transfected the reporter construct and a pRL-TK Renilla luciferase reporter (Promega) into EndoC-bH1 cells using lipofectamine 2000 (Thermo Fisher). 48 hours after transfection, we lysed cells and loaded the cell lysates into a 96-well plate to detect luciferase activity using a Synergy H1 Microplate reader. We calculated relative luciferase activity, normalizing firefly luciferase activity to that of Renilla luciferase. We performed 12 replicates for each DNA fragment and compared the relative luciferase activity between each allele using the Wilcoxon rank sum test.

Data availability

The models from this study (architecture and weights), enhancer locations, EDR/ESR locations, and IEP scores for gnomAD SNPs are available through zenodo (https://zenodo.org/record/8161621). The models are also available at kipoi (http://kipoi.org/models/TREDNet).

References

1. ENCODE Project Consortium, An integrated encyclopedia of DNA elements in the human genome. *Nature* **489**, 57–74 (2012).
2. Roadmap Epigenomics Consortium, *et al.*, Integrative analysis of 111 reference human epigenomes. *Nature* **518**, 317–330 (2015).
3. A. Viñuela, *et al.*, Genetic variant effects on gene expression in human pancreatic islets and their implications for T2D. *Nat. Commun.* **11**, 4912 (2020).
4. S. C. J. Parker, *et al.*, Chromatin stretch enhancer states drive cell-specific gene regulation and harbor human disease risk variants. *Proc Natl Acad Sci USA* **110**, 17921–17926 (2013).
5. J. Zhou, *et al.*, Deep learning sequence-based ab initio prediction of variant effects on expression and disease risk. *Nat. Genet.* **50**, 1171–1179 (2018).
6. J. Zhou, O. G. Troyanskaya, Predicting effects of noncoding variants with deep learning-based sequence model. *Nat. Methods* **12**, 931–934 (2015).
7. D. R. Kelley, J. Snoek, J. L. Rinn, Basset: learning the regulatory code of the accessible genome with deep convolutional neural networks. *Genome Res.* **26**, 990–999 (2016).
8. B. Yang, *et al.*, BiRen: predicting enhancers with a deep-learning-based model using the DNA sequence alone. *Bioinformatics* **33**, 1930–1936 (2017).
9. K. K. Tan, N. Q. K. Le, H.-Y. Yeh, M. C. H. Chua, Ensemble of deep recurrent neural networks for identifying enhancers via dinucleotide physicochemical properties. *Cells* **8** (2019).
10. D. Lee, LS-GKM: a new gkm-SVM for large-scale datasets. *Bioinformatics* **32**, 2196–2198 (2016).
11. P. Kheradpour, *et al.*, Systematic dissection of regulatory motifs in 2000 predicted human enhancers using a massively parallel reporter assay. *Genome Res.* **23**, 800–811 (2013).
12. M. Kircher, *et al.*, Saturation mutagenesis of twenty disease-associated regulatory elements at single base-pair resolution. *Nat. Commun.* **10**, 3583 (2019).
13. J. C. Kwasnieski, C. Fiore, H. G. Chaudhari, B. A. Cohen, High-throughput functional testing of ENCODE segmentation predictions. *Genome Res.* **24**, 1595–1602 (2014).
14. Y. Benjamini, Y. Hochberg, Controlling the false discovery rate: A practical and powerful approach to multiple testing. *Journal of the Royal Statistical Society. Series B (Methodological)* **57**, 289–300 (1995).
15. S. Heinz, *et al.*, Simple combinations of lineage-determining transcription factors prime cis-regulatory elements required for macrophage and B cell identities. *Mol. Cell* **38**, 576–589 (2010).
16. A. Varshney, *et al.*, Genetic regulatory signatures underlying islet gene expression and type 2 diabetes. *Proc Natl Acad Sci USA* **114**, 2301–2306 (2017).
17. C. E. Grant, T. L. Bailey, W. S. Noble, FIMO: scanning for occurrences of a given motif.

Bioinformatics **27**, 1017–1018 (2011).

18. L. J. Scott, *et al.*, The genetic regulatory signature of type 2 diabetes in human skeletal muscle. *Nat. Commun.* **7**, 11764 (2016).
19. P. Kheradpour, M. Kellis, Systematic discovery and characterization of regulatory motifs in ENCODE TF binding experiments. *Nucleic Acids Res.* **42**, 2976–2987 (2014).
20. A. Mathelier, *et al.*, JASPAR 2016: a major expansion and update of the open-access database of transcription factor binding profiles. *Nucleic Acids Res.* **44**, D110-5 (2016).
21. A. Jolma, *et al.*, DNA-binding specificities of human transcription factors. *Cell* **152**, 327–339 (2013).
22. R. Pique-Regi, *et al.*, Accurate inference of transcription factor binding from DNA sequence and chromatin accessibility data. *Genome Res.* **21**, 447–455 (2011).
23. G. M. Cooper, *et al.*, Distribution and intensity of constraint in mammalian genomic sequence. *Genome Res.* **15**, 901–913 (2005).
24. A. Siepel, K. S. Pollard, D. Haussler, "New Methods for Detecting Lineage-Specific Selection" in *Research in Computational Molecular Biology*, Lecture notes in computer science., A. Apostolico, C. Guerra, S. Istrail, P. A. Pevzner, M. Waterman, Eds. (Springer Berlin Heidelberg, 2006), pp. 190–205.
25. J. van Arensbergen, *et al.*, High-throughput identification of human SNPs affecting regulatory element activity. *Nat. Genet.* **51**, 1160–1169 (2019).
26. K. J. Karczewski, *et al.*, The mutational constraint spectrum quantified from variation in 141,456 humans. *Nature* **581**, 434–443 (2020).
27. S. Khetan, *et al.*, Type 2 Diabetes-Associated Genetic Variants Regulate Chromatin Accessibility in Human Islets. *Diabetes* **67**, 2466–2477 (2018).
28. S. Khetan, *et al.*, Functional characterization of T2D-associated SNP effects on baseline and ER stress-responsive β cell transcriptional activation. *Nat. Commun.* **12**, 5242 (2021).
29. V. Iotchkova, *et al.*, GARFIELD classifies disease-relevant genomic features through integration of functional annotations with association signals. *Nat. Genet.* **51**, 343–353 (2019).
30. R. Paternoster, R. Brame, P. Mazerolle, A. Piquero, Using the correct statistical test for the equality of regression coefficients. *Criminology.* **36**, 859–866 (1998).
31. 1000 Genomes Project Consortium, *et al.*, A global reference for human genetic variation. *Nature* **526**, 68–74 (2015).
32. G. Korotkevich, *et al.*, Fast gene set enrichment analysis. *BioRxiv* (2016) https:/doi.org/10.1101/060012.
33. A. Mahajan, *et al.*, Fine-mapping type 2 diabetes loci to single-variant resolution using high-density imputation and islet-specific epigenome maps. *Nat. Genet.* **50**, 1505–1513 (2018).
34. A. Mahajan, *et al.*, Multi-ancestry genetic study of type 2 diabetes highlights the power of

diverse populations for discovery and translation. *Nat. Genet.* **54**, 560–572 (2022).

35. J. Dupuis, *et al.*, New genetic loci implicated in fasting glucose homeostasis and their impact on type 2 diabetes risk. *Nat. Genet.* **42**, 105–116 (2010).

36. N. Sinnott-Armstrong, *et al.*, Genetics of 35 blood and urine biomarkers in the UK Biobank. *Nat. Genet.* **53**, 185–194 (2021).

37. V. Rai, *et al.*, Single-cell ATAC-Seq in human pancreatic islets and deep learning upscaling of rare cells reveals cell-specific type 2 diabetes regulatory signatures. *Mol. Metab.* **32**, 109–121 (2020).

38. W. W. Greenwald, *et al.*, Pancreatic islet chromatin accessibility and conformation reveals distal enhancer networks of type 2 diabetes risk. *Nat. Commun.* **10**, 2078 (2019).

39. H. Li, R. Durbin, Fast and accurate long-read alignment with Burrows-Wheeler transform. *Bioinformatics* **26**, 589–595 (2010).

40. B. van de Geijn, G. McVicker, Y. Gilad, J. K. Pritchard, WASP: allele-specific software for robust molecular quantitative trait locus discovery. *Nat. Methods* **12**, 1061–1063 (2015).

41. S. Samuel A., S. Edward A., D. Leland C., S. Shirley A., W. Jr. Robin M., *The American Soldier: Adjustment During Army Life* (Princeton University Press, 1949).

42. T. Lipták, On the combination of independent tests. *Magyar Tud Akad Mat Kutato Int Kozl* **3**, 171–197 (1958).

43. M. Benazra, *et al.*, A human beta cell line with drug inducible excision of immortalizing transgenes. *Mol. Metab.* **4**, 916–925 (2015).

PNAS

DIAMANTE Consortium Authors

The following authors were part of the Diabetes Meta-Analysis of Trans-Ethnic association studies (DIAMANTE) Consortium:

Anubha Mahajan[1,2,278], Cassandra N. Spracklen[3,4], Weihua Zhang[5,6], Maggie C. Y. Ng[7,8,9], Lauren E Petty[7], Hidetoshi Kitajima[2,10,11,12], Grace Z. Yu[1,2], Sina Rüeger[13], Leo Speidel[14,15], Young Jin Kim[16], Momoko Horikoshi[17], Josep M. Mercader[18,19,20], Daniel Taliun[21], Sanghoon Moon[16], Soo-Heon Kwak[22], Neil R. Robertson[1,2], Nigel W. Rayner[1,2,23,24], Marie Loh[5,25,26], Bong-Jo Kim[16], Joshua Chiou[27,279], Irene Miguel-Escalada[28,29], Pietro della Briotta Parolo[13], Kuang Lin[30], Fiona Bragg[30,31], Michael H. Preuss[32], Fumihiko Takeuchi[33], Jana Nano[34], Xiuqing Guo[35], Amel Lamri[36,37], Masahiro Nakatochi[38], Robert A. Scott[39], Jung-Jin Lee[40], Alicia Huerta-Chagoya[41,280], Mariaelisa Graff[42], Jin-Fang Chai[43], Esteban J Parra[44], Jie Yao[35], Lawrence F. Bielak[45], Yasuharu Tabara[46], Yang Hai[35], Valgerdur Steinthorsdottir[47], James P. Cook[48], Mart Kals[49], Niels Grarup[50], Ellen M. Schmidt[21], Ian Pan[51], Tamar Sofer[52,53,54], Matthias Wuttke[55], Chloe Sarnowski[56,281], Christian Gieger[57,58,59], Darryl Nousome[60], Stella Trompet[61,62], Jirong Long[63], Meng Sun[2], Lin Tong[64], Wei-Min Chen[65], Meraj Ahmad[66], Raymond Noordam[62], Victor J. Y. Lim[43], Claudia H. T. Tam[67,68], Yoonjung Yoonie Joo[69,70,282], Chien-Hsiun Chen[71], Laura M. Raffield[3], Cécile Lecoeur[72,73], Bram Peter Prins[23], Aude Nicolas[74], Lisa R. Yanek[75], Guanjie Chen[76], Richard A. Jensen[77], Salman Tajuddin[78], Edmond K. Kabagambe[63,283], Ping An[79], Anny H. Xiang[80], Hyeok Sun Choi[81], Brian E. Cade[20,53], Jingyi Tan[35], Jack Flanagan[17,48], Fernando Abaitua[2,284], Linda S. Adair[82], Adebowale Adeyemo[76], Carlos A. Aguilar-Salinas[83], Masato Akiyama[84,85], Sonia S. Anand[36,37,86], Alain Bertoni[87], Zheng Bian[88], Jette Bork-Jensen[50], Ivan Brandslund[89,90], Jennifer A. Brody[77], Chad M. Brummett[91], Thomas A. Buchanan[92], Mickaël Canouil[72,73], Juliana C. N. Chan[67,68,93,94], Li-Ching Chang[71], Miao-Li Chee[95], Ji Chen[96,285], Shyh-Huei Chen[97], Yuan-Tsong Chen[71], Zhengming Chen[30,31], Lee-Ming Chuang[98,99], Mary Cushman[100], Swapan K. Das[101], H. Janaka de Silva[102], George Dedoussis[103], Latchezar Dimitrov[8], Ayo P. Doumatey[76], Shufa Du[82,104], Qing Duan[3], Kai-Uwe Eckardt[105,106], Leslie S. Emery[107], Daniel S. Evans[108], Michele K. Evans[78], Krista Fischer[49], James S. Floyd[77], Ian Ford[109], Myriam Fornage[110], Oscar H. Franco[34], Timothy M. Frayling[111], Barry I. Freedman[112], Christian Fuchsberger[21,113], Pauline Genter[114], Hertzel C. Gerstein[36,37,86], Vilmantas Giedraitis[115], Clicerio González-Villalpando[116], Maria Elena González-Villalpando[116], Mark O. Goodarzi[117], Penny Gordon-Larsen[82,104], David Gorkin[118], Myron Gross[119], Yu Guo[88], Sophie Hackinger[23], Sohee Han[16], Andrew T. Hattersley[120], Christian Herder[57,121,122], Annie-Green Howard[104,123], Willa Hsueh[124], Mengna Huang[51,125], Wei Huang[126], Yi-Jen Hung[127,128], Mi Yeong Hwang[16], Chii-Min Hwu[129,130], Sahoko Ichihara[131], Mohammad Arfan Ikram[34], Martin Ingelsson[115], Md Tariqul Islam[132], Masato Isono[33], Hye-Mi Jang[16], Farzana Jasmine[64], Guozhi Jiang[67,68], Jost B. Jonas[133], Marit E. Jørgensen[134,135], Torben Jørgensen[136,137,138], Yoichiro Kamatani[84,139], Fouad R. Kandeel[140], Anuradhani Kasturiratne[141], Tomohiro Katsuya[142,143], Varinderpal Kaur[19], Takahisa Kawaguchi[46], Jacob M. Keaton[8,63,286], Abel N. Kho[144,145], Chiea-Chuen Khor[146], Muhammad G. Kibriya[64], Duk-Hwan Kim[147], Katsuhiko Kohara[148,287], Jennifer Kriebel[57,58,59], Florian Kronenberg[149], Johanna Kuusisto[150], Kristi Läll[49,151], Leslie A. Lange[152], Myung-Shik Lee[153,154], Nanette R. Lee[155], Aaron Leong[19,156,157], Liming Li[158], Yun Li[3], Ruifang Li-Gao[159], Symen Ligthart[34], Cecilia M. Lindgren[2,160,161], Allan Linneberg[136,162], Ching-Ti Liu[56], Jianjun Liu[146,163], Adam E. Locke[164,165,288], Tin Louie[107], Jian'an Luan[39], Andrea O. Luk[67,68], Xi Luo[166], Jun Lv[158], Valeriya Lyssenko[167,168], Vasiliki Mamakou[169], K. Radha Mani[66,277], Thomas Meitinger[170,171,172], Andres Metspalu[49], Andrew D. Morris[173], Girish N. Nadkarni[32,174,175], Jerry L. Nadler[176], Michael A. Nalls[74,177,178], Uma Nayak[65], Suraj S. Nongmaithem[66], Ioanna Ntalla[179], Yukinori Okada[180,181,182], Lorena Orozco[183], Sanjay R. Patel[184], Mark A. Pereira[185], Annette Peters[57,58,172], Fraser J. Pirie[186], Bianca Porneala[157], Gauri Prasad[187,188], Sebastian Preissl[118], Laura J. Rasmussen-Torvik[189], Alexander P. Reiner[190], Michael Roden[57,121,122], Rebecca Rohde[42], Kathryn Roll[35], Charumathi Sabanayagam[95,191,192], Maike Sander[193,194,195], Kevin Sandow[35], Naveed Sattar[196], Sebastian Schönherr[149], Claudia Schurmann[32,174,197], Mohammad Shahriar[64,289], Jinxiu Shi[126], Dong Mun Shin[16], Daniel Shriner[76], Jennifer A. Smith[45,198], Wing Yee So[67,93], Alena Stančáková[150], Adrienne M. Stilp[107], Konstantin Strauch[199,200,201], Ken Suzuki[17,84,180,202], Atsushi Takahashi[84,203], Kent D. Taylor[35], Barbara Thorand[57,58], Gudmar Thorleifsson[47], Unnur Thorsteinsdottir[47,204], Brian Tomlinson[67,205], Jason M. Torres[2,290], Fuu-Jen Tsai[206], Jaakko Tuomilehto[207,208,209,210], Teresa Tusie-Luna[211,212], Miriam S. Udler[18,19,156], Adan Valladares-Salgado[213], Rob M. van Dam[43,163], Jan B. van Klinken[214,215,216], Rohit Varma[217], Marijana Vujkovic[218], Niels Wacher-Rodarte[219], Eleanor Wheeler[39], Eric A. Whitsel[42,220], Ananda R. Wickremasinghe[141], Ko Willems van Dijk[214,215,221], Daniel R. Witte[222,223], Chittaranjan S. Yajnik[224], Ken Yamamoto[225], Toshimasa Yamauchi[202], Loïc Yengo[226], Kyungheon Yoon[16], Canqing Yu[158], Jian-Min Yuan[227,228], Salim Yusuf[36,37,86], Liang Zhang[95], Wei Zheng[63], FinnGen, eMERGE Consortium, Leslie J. Raffel[229], Michiya Igase[230], Eli Ipp[114], Susan Redline[20,53,231], Yoon Shin Cho[81], Lars Lind[232],

Michael A. Province[79], Craig L. Hanis[233], Patricia A. Peyser[45], Erik Ingelsson[234,235], Alan B. Zonderman[78], Bruce M. Psaty[77,236,237], Ya-Xing Wang[238], Charles N. Rotimi[76], Diane M. Becker[75], Fumihiko Matsuda[46], Yongmei Liu[87,239], Eleftheria Zeggini[23,24,240], Mitsuhiro Yokota[241], Stephen S. Rich[242], Charles Kooperberg[190], James S. Pankow[185], James C. Engert[243,244], Yii-Der Ida Chen[35], Philippe Froguel[72,73,245], James G. Wilson[246], Wayne H. H. Sheu[128,130,247], Sharon L. R. Kardia[45], Jer-Yuarn Wu[71], M. Geoffrey Hayes[69,248,249], Ronald C. W. Ma[67,68,93,94], Tien-Yin Wong[95,191,192], Leif Groop[13,167], Dennis O. Mook-Kanamori[159], Giriraj R. Chandak[66], Francis S. Collins[250], Dwaipayan Bharadwaj[187,251], Guillaume Paré[37,252], Michèle M. Sale[65,277], Habibul Ahsan[64], Ayesha A. Motala[186], Xiao-Ou Shu[63], Kyong-Soo Park[22,253,254], J. Wouter Jukema[61,255], Miguel Cruz[213], Roberta McKean-Cowdin[60], Harald Grallert[57,58,59], Ching-Yu Cheng[95,191,192], Erwin P. Bottinger[32,174,197], Abbas Dehghan[5,34,256], E-Shyong Tai[43,163,257], Josée Dupuis[56], Norihiro Kato[33], Markku Laakso[150], Anna Köttgen[55], Woon-Puay Koh[258,259], Colin N. A. Palmer[260], Simin Liu[51,125,261], Goncalo Abecasis[21], Jaspal S. Kooner[6,256,262,263], Ruth J. F. Loos[32,50,264], Kari E. North[42], Christopher A. Haiman[60], Jose C. Florez[18,19,156], Danish Saleheen[40,265,266], Torben Hansen[50], Oluf Pedersen[50], Reedik Mägi[49], Claudia Langenberg[39,267], Nicholas J. Wareham[39], Shiro Maeda[17,268,269], Takashi Kadowaki[202,291], Juyoung Lee[16], Iona Y. Millwood[30,31], Robin G. Walters[30,31], Kari Stefansson[47,204], Simon R. Myers[2,270], Jorge Ferrer[28,29,271], Kyle J. Gaulton[193,194], James B. Meigs[18,156,157], Karen L. Mohlke[3], Anna L. Gloyn[1,2,272,273], Donald W. Bowden[8,9,274], Jennifer E. Below[7], John C. Chambers[5,6,25,256,262], Xueling Sim[43], Michael Boehnke[21], Jerome I. Rotter[35], Mark I. McCarthy[1,2,272,278], and Andrew P. Morris[2,48,49,275,276]

[1]Oxford Centre for Diabetes, Endocrinology and Metabolism, Radcliffe Department of Medicine, University of Oxford, Oxford, UK. [2]Wellcome Centre for Human Genetics, Nuffield Department of Medicine, University of Oxford, Oxford, UK. [3]Department of Genetics, University of North Carolina at Chapel Hill, Chapel Hill, NC, USA. [4]Department of Epidemiology and Biostatistics, University of Massachusetts-Amherst, Amherst, MA, USA. [5]Department of Epidemiology and Biostatistics, Imperial College London, London, UK. [6]Department of Cardiology, Ealing Hospital, London North West Healthcare NHS Trust, London, UK. [7]Vanderbilt Genetics Institute, Division of Genetic Medicine, Vanderbilt University Medical Center, Nashville, TN, USA. [8]Center for Genomics and Personalized Medicine Research, Wake Forest School of Medicine, Winston-Salem, NC, USA. [9]Department of Biochemistry, Wake Forest School of Medicine, Winston-Salem, NC, USA. [10]The Advanced Research Center for Innovations in Next-Generation Medicine (INGEM), Tohoku University, Sendai, Japan. [11]Department of Integrative Genomics, Tohoku Medical Megabank Organization, Tohoku University, Sendai, Japan. [12]Cancer Center, Tohoku University Hospital, Tohoku University, Sendai, Japan. [13]Institute for Molecular Medicine Finland (FIMM), University of Helsinki, Helsinki, Finland. [14]Genetics Institute, University College London, London, UK. [15]Francis Crick Institute, London, UK. [16]Division of Genome Science, Department of Precision Medicine, National Institute of Health, Cheongju-si, Republic of Korea. [17]Laboratory for Genomics of Diabetes and Metabolism, RIKEN Center for Integrative Medical Sciences, Yokohama, Japan. [18]Programs in Metabolism and Medical & Population Genetics, Broad Institute of Harvard and MIT, Cambridge, MA, USA. [19]Diabetes Unit and Center for Genomic Medicine, Massachusetts General Hospital, Boston, MA, USA. [20]Harvard Medical School, Boston, MA, USA. [21]Department of Biostatistics and Center for Statistical Genetics, University of Michigan, Ann Arbor, MI, USA. [22]Department of Internal Medicine, Seoul National University Hospital, Seoul, South Korea. [23]Department of Human Genetics, Wellcome Sanger Institute, Hinxton, UK. [24]Institute of Translational Genomics, Helmholtz Zentrum München, German Research Center for Environmental Health, Neuherberg, Germany. [25]Lee Kong Chian School of Medicine, Nanyang Technological University, Singapore, Singapore. [26]Translational Laboratory in Genetic Medicine (TLGM), Agency for Science, Technology and Research (A*STAR) and National University of Singapore (NUS), Singapore, Singapore. [27]Biomedical Sciences Graduate Studies Program, University of California San Diego, La Jolla, CA, USA. [28]Regulatory Genomics and Diabetes, Centre for Genomic Regulation, The Barcelona Institute of Science and Technology, Barcelona, Spain. [29]Centro de Investigación Biomédica en Red Diabetes y Enfermedades Metabólicas asociadas (CIBERDEM), Madrid, Spain. [30]Nuffield Department of Population Health, University of Oxford, Oxford, UK. [31]Medical Research Council Population Health Research Unit, University of Oxford, Oxford, UK. [32]The Charles Bronfman Institute for Personalized Medicine, Icahn School of Medicine at Mount Sinai, New York, NY, USA. [33]Department of Gene Diagnostics and Therapeutics, Research Institute, National Center for Global Health and Medicine, Tokyo, Japan. [34]Department of Epidemiology, Erasmus University Medical Center, Rotterdam, The Netherlands. [35]The Institute for Translational Genomics and Population Sciences, Department of Pediatrics, The Lundquist Institute for Biomedical Innovation (formerly Los Angeles Biomedical Research Institute) at Harbor-UCLA Medical Center, Torrance, CA, USA. [36]Department of Medicine, McMaster University, Hamilton, ON, Canada. [37]Population Health Research Institute, Hamilton Health Sciences and McMaster University, Hamilton, ON, Canada. [38]Public Health Informatics Unit, Department of Integrated Health Sciences, Nagoya University Graduate School of Medicine, Nagoya, Japan. [39]MRC Epidemiology Unit, Institute of Metabolic Science, University of Cambridge, Cambridge, UK. [40]Division of Translational Medicine and Human Genetics, University of Pennsylvania, Philadelphia, PA, USA. [41]Consejo Nacional de Ciencia y Tecnología (CONACYT), Instituto Nacional de Ciencias Médicas y Nutrición Salvador Zubirán, Mexico City, Mexico. [42]Department of Epidemiology, Gillings School of Global Public Health, University of North Carolina at Chapel Hill, Chapel Hill, NC, USA. [43]Saw Swee Hock School of Public Health, National University of

Singapore and National University Health System, Singapore, Singapore. [44]Department of Anthropology, University of Toronto at Mississauga, Mississauga, ON, Canada. [45]Department of Epidemiology, School of Public Health, University of Michigan, Ann Arbor, MI, USA. [46]Center for Genomic Medicine, Kyoto University Graduate School of Medicine, Kyoto, Japan. [47]deCODE Genetics, Amgen inc., Reykjavik, Iceland. [48]Department of Health Data Science, University of Liverpool, Liverpool, UK. [49]Estonian Genome Centre, Institute of Genomics, University of Tartu, Tartu, Estonia. [50]Novo Nordisk Foundation Center for Basic Metabolic Research, Faculty of Health and Medical Sciences, University of Copenhagen, Copenhagen, Denmark. [51]Department of Epidemiology, Brown University School of Public Health, Providence, RI, USA. [52]Department of Biostatistics, Harvard University, Boston, MA, USA. [53]Division of Sleep and Circadian Disorders, Brigham and Women's Hospital, Boston, MA, USA. [54]Department of Medicine, Harvard University, Boston, MA, USA. [55]Institute of Genetic Epidemiology, Department of Data Driven Medicine, Faculty of Medicine and Medical Center, University of Freiburg, Freiburg, Germany. [56]Department of Biostatistics, Boston University School of Public Health, Boston, MA, USA. [57]German Center for Diabetes Research (DZD), Neuherberg, Germany. [58]Institute of Epidemiology, Helmholtz Zentrum München, German Research Center for Environmental Health, Neuherberg, Germany. [59]Research Unit of Molecular Epidemiology, Helmholtz Zentrum München, German Research Center for Environmental Health, Neuherberg, Germany. [60]Department of Population and Public Health Sciences, Keck School of Medicine of USC, Los Angeles, CA, USA. [61]Department of Cardiology, Leiden University Medical Center, Leiden, The Netherlands. [62]Section of Gerontology and Geriatrics, Department of Internal Medicine, Leiden University Medical Center, Leiden, The Netherlands. [63]Division of Epidemiology, Department of Medicine, Institute for Medicine and Public Health, Vanderbilt Genetics Institute, Vanderbilt University Medical Center, Nashville, TN, USA. [64]Institute for Population and Precision Health, The University of Chicago, Chicago, IL, USA. [65]Department of Public Health Sciences and Center for Public Health Genomics, University of Virginia School of Medicine, Charlottesville, VA, USA. [66]Genomic Research on Complex Diseases (GRC-Group), CSIR-Centre for Cellular and Molecular Biology (CSIR-CCMB), Hyderabad, India. [67]Department of Medicine and Therapeutics, The Chinese University of Hong Kong, Hong Kong, China. [68]Chinese University of Hong Kong-Shanghai Jiao Tong University Joint Research Centre in Diabetes Genomics and Precision Medicine, The Chinese University of Hong Kong, Hong Kong, China. [69]Division of Endocrinology, Metabolism, and Molecular Medicine, Department of Medicine, Northwestern University Feinberg School of Medicine, Chicago, IL, USA. [70]Department of Health and Biomedical Informatics, Northwestern University Feinberg School of Medicine, Chicago, IL, USA. [71]Institute of Biomedical Sciences, Academia Sinica, Taipei, Taiwan. [72]Inserm U1283, CNRS UMR 8199, European Genomic Institute for Diabetes, Institut Pasteur de Lille, Lille, France. [73]University of Lille, Lille University Hospital, Lille, France. [74]Laboratory of Neurogenetics, National Institute on Aging, National Institutes of Health, Bethesda, MD, USA. [75]Department of Medicine, Johns Hopkins University School of Medicine, Baltimore, MD, USA. [76]Center for Research on Genomics and Global Health, National Human Genome Research Institute, National Institutes of Health, Bethesda, MD, USA. [77]Cardiovascular Health Research Unit, Department of Medicine, University of Washington, Seattle, WA, USA. [78]Laboratory of Epidemiology and Population Sciences, National Institute on Aging, National Institutes of Health, Baltimore, MD, USA. [79]Division of Statistical Genomics, Washington University School of Medicine, St. Louis, MO, USA. [80]Department of Research and Evaluation, Division of Biostatistics Research, Kaiser Permanente of Southern California, Pasadena, CA, USA. [81]Department of Biomedical Science, Hallym University, Chuncheon, South Korea. [82]Department of Nutrition, Gillings School of Global Public Health, University of North Carolina at Chapel Hill, Chapel Hill, NC, USA. [83]Unidad de Investigación en Enfermedades Metabólicas and Departamento de Endocrinología y Metabolismo, Instituto Nacional de Ciencias Médicas y Nutrición Salvador Zubirán, Mexico City, Mexico. [84]Laboratory for Statistical and Translational Genetics, RIKEN Center for Integrative Medical Sciences, Yokohama, Japan. [85]Department of Ocular Pathology and Imaging Science, Graduate School of Medical Sciences, Kyushu University, Fukuoka, Japan. [86]Department of Health Research Methods, Evidence, and Impact, McMaster University, Hamilton, ON, Canada. [87]Department of Epidemiology and Prevention, Division of Public Health Sciences, Wake Forest School of Medicine, Winston-Salem, NC, USA. [88]Chinese Academy of Medical Sciences, Beijing, China. [89]Institute of Regional Health Research, University of Southern Denmark, Odense, Denmark. [90]Department of Clinical Biochemistry, Vejle Hospital, Vejle, Denmark. [91]Department of Anesthesiology, University of Michigan Medical School, Ann Arbor, MI, USA. [92]Department of Medicine, Division of Endocrinology and Diabetes, Keck School of Medicine of USC, Los Angeles, CA, USA. [93]Hong Kong Institute of Diabetes and Obesity, The Chinese University of Hong Kong, Hong Kong, China. [94]Li Ka Shing Institute of Health Sciences, The Chinese University of Hong Kong, Hong Kong, China. [95]Singapore Eye Research Institute, Singapore National Eye Centre, Singapore, Singapore. [96]Wellcome Sanger Institute, Hinxton, UK. [97]Department of Biostatistics and Data Science, Wake Forest School of Medicine, Winston-Salem, NC, USA. [98]Division of Endocrinology and Metabolism, Department of Internal Medicine, National Taiwan University Hospital, Taipei, Taiwan. [99]Institute of Epidemiology and Preventive Medicine, National Taiwan University, Taipei, Taiwan. [100]Department of Medicine, University of Vermont, Colchester, VT, USA. [101]Section on Endocrinology and Metabolism, Department of Internal Medicine, Wake Forest School of Medicine, Winston-Salem, NC, USA. [102]Department of Medicine, Faculty of Medicine, University of Kelaniya, Ragama, Sri Lanka. [103]Department of Nutrition and Dietetics, Harokopio University of Athens, Athens, Greece. [104]Carolina Population Center, University of North

Carolina at Chapel Hill, Chapel Hill, NC, USA. [105]Department of Nephrology and Medical Intensive Care Medicine, Charité Universitätsmedizin Berlin, Berlin, Germany. [106]Department of Nephrology and Hypertension, Friedrich-Alexander-Universität Erlangen-Nürnberg, Erlangen, Germany. [107]Department of Biostatistics, University of Washington, Seattle, WA, USA. [108]California Pacific Medical Center Research Institute, San Francisco, CA, USA. [109]Robertson Centre for Biostatistics, University of Glasgow, Glasgow, UK. [110]Institute of Molecular Medicine, University of Texas Health Science Center at Houston, Houston, TX, USA. [111]Genetics of Complex Traits, University of Exeter Medical School, University of Exeter, Exeter, UK. [112]Department of Internal Medicine, Wake Forest School of Medicine, Winston-Salem, NC, USA. [113]Institute for Biomedicine, Eurac Research, Affiliated Institute of the University of Lübeck, Bolzano, Italy. [114]Department of Medicine, Division of Endocrinology and Metabolism, Lundquist Research Institute at Harbor-UCLA Medical Center, Torrance, CA, USA. [115]Department of Public Health and Caring Sciences, Uppsala University, Uppsala, Sweden. [116]Centro de Estudios en Diabetes, Unidad de Investigacion en Diabetes y Riesgo Cardiovascular, Centro de Investigacion en Salud Poblacional, Instituto Nacional de Salud Publica, Mexico City, Mexico. [117]Department of Medicine, Division of Endocrinology, Diabetes and Metabolism, Cedars-Sinai Medical Center, Los Angeles, CA, USA. [118]Center for Epigenomics, University of California San Diego, La Jolla, CA, USA. [119]Department of Laboratory Medicine and Pathology, University of Minnesota, Minneapolis, MN, USA. [120]University of Exeter Medical School, University of Exeter, Exeter, UK. [121]Institute for Clinical Diabetology, German Diabetes Center, Leibniz Center for Diabetes Research at Heinrich Heine University Düsseldorf, Düsseldorf, Germany. [122]Department of Endocrinology and Diabetology, Medical Faculty and University Hospital Düsseldorf, Heinrich Heine University Düsseldorf, Düsseldorf, Germany. [123]Department of Biostatistics, Gillings School of Global Public Health, University of North Carolina at Chapel Hill, Chapel Hill, NC, USA. [124]Department of Internal Medicine, Diabetes and Metabolism Research Center, The Ohio State University Wexner Medical Center, Columbus, OH, USA. [125]Center for Global Cardiometabolic Health, Brown University, Providence, RI, USA. [126]Shanghai-MOST Key Laboratory of Health and Disease Genomics, Chinese National Human Genome Center at Shanghai (CHGC) and Shanghai Institute for Biomedical and Pharmaceutical Technologies (SIBPT), Shanghai, China. [127]Division of Endocrine and Metabolism, Tri-Service General Hospital Songshan Branch, Taipei, Taiwan. [128]School of Medicine, National Defense Medical Center, Taipei, Taiwan. [129]Section of Endocrinology and Metabolism, Department of Medicine, Taipei Veterans General Hospital, Taipei, Taiwan. [130]School of Medicine, National Yang Ming Chiao Tung University, Taipei, Taiwan. [131]Department of Environmental and Preventive Medicine, Jichi Medical University School of Medicine, Shimotsuke, Japan. [132]University of Chicago Research Bangladesh, Dhaka, Bangladesh. [133]Institute of Molecular and Clinical Ophthalmology Basel, Basel, Switzerland. [134]Steno Diabetes Center Copenhagen, Gentofte, Denmark. [135]National Institute of Public Health, Southern Denmark University, Copenhagen, Denmark. [136]Center for Clinical Research and Prevention, Bispebjerg and Frederiksberg Hospital, Frederiksberg, Denmark. [137]Faculty of Health and Medical Sciences, University of Copenhagen, Copenhagen, Denmark. [138]Faculty of Medicine, Aalborg University, Aalborg, Denmark. [139]Laboratory of Complex Trait Genomics, Department of Computational Biology and Medical Sciences, Graduate School of Frontier Sciences, The University of Tokyo, Tokyo, Japan. [140]Department of Clinical Diabetes, Endocrinology & Metabolism, Department of Translational Research and Cellular Therapeutics, City of Hope, Duarte, CA, USA. [141]Department of Public Health, Faculty of Medicine, University of Kelaniya, Ragama, Sri Lanka. [142]Department of Clinical Gene Therapy, Osaka University Graduate School of Medicine, Osaka, Japan. [143]Department of Geriatric and General Medicine, Graduate School of Medicine, Osaka University, Osaka, Japan. [144]Division of General Internal Medicine and Geriatrics, Department of Medicine, Northwestern University Feinberg School of Medicine, Chicago, IL, USA. [145]Center for Health Information Partnerships, Institute for Public Health and Medicine, Northwestern University Feinberg School of Medicine, Chicago, IL, USA. [146]Genome Institute of Singapore, Agency for Science, Technology and Research, Singapore, Singapore. [147]Department of Molecular Cell Biology, Sungkyunkwan University School of Medicine, Suwon, South Korea. [148]Department of Regional Resource Management, Ehime University Faculty of Collaborative Regional Innovation, Ehime, Japan. [149]Institute of Genetic Epidemiology, Department of Genetics and Pharmacology, Medical University of Innsbruck, Innsbruck, Austria. [150]Institute of Clinical Medicine, Internal Medicine, University of Eastern Finland and Kuopio University Hospital, Kuopio, Finland. [151]Institute of Mathematics and Statistics, University of Tartu, Tartu, Estonia. [152]Department of Medicine, University of Colorado Denver, Anschutz Medical Campus, Aurora, CO, USA. [153]Severance Biomedical Science Institute and Department of Internal Medicine, Yonsei University College of Medicine, Seoul, South Korea. [154]Department of Medicine, Samsung Medical Center, Sungkyunkwan University School of Medicine, Seoul, South Korea. [155]USC-Office of Population Studies Foundation, Inc., University of San Carlos, Cebu City, Philippines. [156]Department of Medicine, Harvard Medical School, Boston, MA, USA. [157]Division of General Internal Medicine, Massachusetts General Hospital, Boston, MA, USA. [158]Department of Epidemiology and Biostatistics, Peking University Health Science Centre, Peking University, Beijing, China. [159]Department of Clinical Epidemiology, Leiden University Medical Center, Leiden, The Netherlands. [160]Program in Medical & Population Genetics, Broad Institute, Cambridge, MA, USA. [161]Big Data Institute, Li Ka Shing Centre for Health Information and Discovery, University of Oxford, Oxford, UK. [162]Department of Clinical Medicine, Faculty of Health and Medical Sciences, University of Copenhagen, Copenhagen, Denmark. [163]Department of Medicine, Yong Loo Lin School of Medicine, National University of Singapore

and National University Health System, Singapore, Singapore. [164]McDonnell Genome Institute, Washington University School of Medicine, St. Louis, MO, USA. [165]Department of Medicine, Division of Genomics and Bioinformatics, Washington University School of Medicine, St. Louis, MO, USA. [166]Department of Biostatistics and Data Science, University of Texas Health Science Center at Houston, Houston, TX, USA. [167]Department of Clinical Sciences, Diabetes and Endocrinology, Lund University Diabetes Centre, Malmö, Sweden. [168]Department of Clinical Science, Center for Diabetes Research, University of Bergen, Bergen, Norway. [169]Dromokaiteio Psychiatric Hospital, National and Kapodistrian University of Athens, Athens, Greece. [170]Institute of Human Genetics, Helmholtz Zentrum München, German Research Center for Environmental Health, Neuherberg, Germany. [171]Institute of Human Genetics, Technical University of Munich, Munich, Germany. [172]German Centre for Cardiovascular Research (DZHK), Partner Site Munich Heart Alliance, Munich, Germany. [173]The Usher Institute to the Population Health Sciences and Informatics, University of Edinburgh, Edinburgh, UK. [174]Digital Health Center, Digital Engineering Faculty of Hasso Plattner Institue and University Potsdam, Potsdam, Germany. [175]The Division of Data Driven and Digital Medicine (D3M), Department of Medicine, Icahn School of Medicine at Mount Sinai, New York, NY, USA. [176]Department of Medicine and Pharmacology, New York Medical College, Valhalla, NY, USA. [177]Data Tecnica International LLC, Glen Echo, MD, USA. [178]Center for Alzheimer's and Related Dementias, National Institutes of Health, Baltimore, MD, USA. [179]William Harvey Research Institute, Barts and The London School of Medicine and Dentistry, Queen Mary University of London, London, UK. [180]Department of Statistical Genetics, Osaka University Graduate School of Medicine, Osaka, Japan. [181]Laboratory of Statistical Immunology, Immunology Frontier Research Center (WPI-IFReC), Osaka University, Osaka, Japan. [182]Laboratory for Systems Genetics, RIKEN Center for Integrative Medical Sciences, Yokohama, Japan. [183]Instituto Nacional de Medicina Genómica, Mexico City, Mexico. [184]Division of Pulmonary, Allergy, and Critical Care Medicine, Department of Medicine, University of Pittsburgh, Pittsburgh, PA, USA. [185]Division of Epidemiology and Community Health, School of Public Health, University of Minnesota, Minneapolis, MN, USA. [186]Department of Diabetes and Endocrinology, Nelson R Mandela School of Medicine, College of Health Sciences, University of KwaZulu-Natal, Durban, South Africa. [187]Academy of Scientific and Innovative Research, CSIR-Human Resource Development Centre Campus, Ghaziabad, Uttar Pradesh, India. [188]Genomics and Molecular Medicine Unit, CSIR-Institute of Genomics and Integrative Biology, New Delhi, India. [189]Department of Preventive Medicine, Northwestern University Feinberg School of Medicine, Chicago, IL, USA. [190]Fred Hutchinson Cancer Research Center, Seattle, WA, USA. [191]Ophthalmology and Visual Sciences Academic Clinical Program (Eye ACP), Duke-NUS Medical School, Singapore, Singapore. [192]Department of Ophthalmology, Yong Loo Lin School of Medicine, National University of Singapore and National University Health System, Singapore, Singapore. [193]Department of Pediatrics, Pediatric Diabetes Research Center, University of California San Diego, La Jolla, CA, USA. [194]Institute for Genomic Medicine, University of California San Diego, La Jolla, CA, USA. [195]Department of Cellular and Molecular Medicine, University of California San Diego, La Jolla, CA, USA. [196]Institute of Cardiovascular and Medical Sciences, University of Glasgow, Glasgow, UK. [197]Hasso Plattner Institute for Digital Health at Mount Sinai, Icahn School of Medicine at Mount Sinai, New York, NY, USA. [198]Survey Research Center, Institute for Social Research, University of Michigan, Ann Arbor, MI, USA. [199]Institute of Genetic Epidemiology, Helmholtz Zentrum München, German Research Center for Environmental Health, Neuherberg, Germany. [200]Chair of Genetic Epidemiology, IBE, Faculty of Medicine, LMU Munich, Munich, Germany. [201]Institute of Medical Biostatistics, Epidemiology and Informatics (IMBEI), University Medical Center, Johannes Gutenberg University, Mainz, Germany. [202]Department of Diabetes and Metabolic Diseases, Graduate School of Medicine, The University of Tokyo, Tokyo, Japan. [203]Department of Genomic Medicine, National Cerebral and Cardiovascular Center, Osaka, Japan. [204]Faculty of Medicine, University of Reykjavik, Reykjavik, Iceland. [205]Faculty of Medicine, Macau University of Science and Technology, Macau, China. [206]Department of Medical Genetics and Medical Research, China Medical University Hospital, Taichung, Taiwan. [207]Department of Health, Finnish Institute for Health and Welfare, Helsinki, Finland. [208]National School of Public Health, Madrid, Spain. [209]Department of Neuroscience and Preventive Medicine, Danube-University Krems, Krems, Austria. [210]Diabetes Research Group, King Abdulaziz University, Jeddah, Saudi Arabia. [211]Unidad de Biología Molecular y Medicina Genómica, Instituto Nacional de Ciencias Médicas y Nutrición Salvador Zubirán, Mexico City, Mexico. [212]Departamento de Medicina Genómica y Toxiología Ambiental, Instituto de Investigaciones Biomédicas, UNAM, Mexico City, Mexico. [213]Unidad de Investigacion Medica en Bioquimica, Hospital de Especialidades, Centro Medico Nacional Siglo XXI, IMSS, Mexico City, Mexico. [214]Einthoven Laboratory for Experimental Vascular Medicine, Leiden University Medical Center, Leiden, The Netherlands. [215]Department of Human Genetics, Leiden University Medical Center, Leiden, The Netherlands. [216]Department of Clinical Chemistry, Laboratory of Genetic Metabolic Disease, Amsterdam University Medical Center, Amsterdam, The Netherlands. [217]Southern California Eye Institute, CHA Hollywood Presbyterian Medical Center, Los Angeles, CA, USA. [218]Department of Medicine, University of Pennsylvania Perelman School of Medicine, Philadelphia, PA, USA. [219]Unidad de Investigación Médica en Epidemiologia Clinica, Hospital de Especialidades, Centro Medico Nacional Siglo XXI, IMSS, Mexico City, Mexico. [220]Department of Medicine, School of Medicine, University of North Carolina at Chapel Hill, Chapel Hill, NC, USA. [221]Department of Internal Medicine, Division of Endocrinology, Leiden University Medical Center, Leiden, The Netherlands. [222]Department of Public Health, Aarhus University, Aarhus, Denmark. [223]Danish Diabetes Academy, Odense, Denmark.

[224]Diabetology Research Centre, King Edward Memorial Hospital and Research Centre, Pune, India. [225]Department of Medical Biochemistry, Kurume University School of Medicine, Kurume, Japan. [226]Institute for Molecular Bioscience, University of Queensland, Brisbane, Australia. [227]Division of Cancer Control and Population Sciences, UPMC Hillman Cancer Center, University of Pittsburgh, Pittsburgh, PA, USA. [228]Department of Epidemiology, Graduate School of Public Health, University of Pittsburgh, Pittsburgh, PA, USA. [229]Department of Pediatrics, Division of Genetic and Genomic Medicine, UCI Irvine School of Medicine, Irvine, CA, USA. [230]Department of Anti-aging Medicine, Ehime University Graduate School of Medicine, Ehime, Japan. [231]Division of Pulmonary, Critical Care, and Sleep Medicine, Beth Israel Deaconess Medical Center, Boston, MA, USA. [232]Department of Medical Sciences, Uppsala University, Uppsala, Sweden. [233]Human Genetics Center, University of Texas Health Science Center at Houston, Houston, TX, USA. [234]Department of Medicine, Division of Cardiovascular Medicine, Stanford University School of Medicine, Stanford, CA, USA. [235]Department of Medical Sciences, Molecular Epidemiology and Science for Life Laboratory, Uppsala University, Uppsala, Sweden. [236]Department of Epidemiology, University of Washington, Seattle, WA, USA. [237]Department of Health Services, University of Washington, Seattle, WA, USA. [238]Beijing Institute of Ophthalmology, Ophthalmology and Visual Sciences Key Laboratory, Beijing Tongren Hospital, Capital Medical University, Beijing, China. [239]Department of Medicine, Division of Cardiology, Duke University School of Medicine, Durham, NC, USA. [240]Technical University of Munich (TUM) and Klinikum Rechts der Isar, TUM School of Medicine, Munich, Germany. [241]Kurume University School of Medicine, Kurume, Japan. [242]Center for Public Health Genomics, University of Virginia School of Medicine, Charlottesville, VA, USA. [243]Department of Medicine, McGill University, Montreal, QC, Canada. [244]Department of Human Genetics, McGill University, Montreal, QC, Canada. [245]Department of Genomics of Common Disease, School of Public Health, Imperial College London, London, UK. [246]Department of Physiology and Biophysics, University of Mississippi Medical Center, Jackson, MS, USA. [247]Division of Endocrinology and Metabolism, Department of Medicine, Taichung Veterans General Hospital, Taichung, Taiwan. [248]Center for Genetic Medicine, Northwestern University Feinberg School of Medicine, Chicago, IL, USA. [249]Department of Anthropology, Northwestern University, Evanston, IL, USA. [250]Center for Precision Health Research, National Human Genome Research Institute, National Institutes of Health, Bethesda, MD, USA. [251]Systems Genomics Laboratory, School of Biotechnology, Jawaharlal Nehru University, New Delhi, India. [252]Department of Pathology and Molecular Medicine, McMaster University, Hamilton, ON, Canada. [253]Department of Internal Medicine, Seoul National University College of Medicine, Seoul, South Korea. [254]Department of Molecular Medicine and Biopharmaceutical Sciences, Graduate School of Convergence Science and Technology, Seoul National University, Seoul, South Korea. [255]Netherlands Heart Institute, Utrecht, The Netherlands. [256]MRC-PHE Centre for Environment and Health, Imperial College London, London, UK. [257]Duke-NUS Medical School, Singapore, Singapore. [258]Singapore Institute for Clinical Sciences, Agency for Science Technology and Research (A*STAR), Singapore, Singapore. [259]Healthy Longevity Translational Research Programme, Yong Loo Lin School of Medicine, National University of Singapore, Singapore, Singapore. [260]Pat Macpherson Centre for Pharmacogenetics and Pharmacogenomics, University of Dundee, Dundee, UK. [261]Department of Medicine, Brown University Alpert School of Medicine, Providence, RI, USA. [262]Imperial College Healthcare NHS Trust, Imperial College London, London, UK. [263]National Heart and Lung Institute, Imperial College London, London, UK. [264]The Mindich Child Health and Development Institute, Ichan School of Medicine at Mount Sinai, New York, NY, USA. [265]Department of Biostatistics and Epidemiology, University of Pennsylvania, Philadelphia, PA, USA. [266]Center for Non-Communicable Diseases, Karachi, Pakistan. [267]Computational Medicine, Berlin Institute of Health at Charité Universitätsmedizin, Berlin, Germany. [268]Department of Advanced Genomic and Laboratory Medicine, Graduate School of Medicine, University of the Ryukyus, Okinawa, Japan. [269]Division of Clinical Laboratory and Blood Transfusion, University of the Ryukyus Hospital, Okinawa, Japan. [270]Department of Statistics, University of Oxford, Oxford, UK. [271]Section of Genetics and Genomics, Department of Metabolism, Digestion and Reproduction, Imperial College London, London, UK. [272]Oxford NIHR Biomedical Research Centre, Churchill Hospital, Oxford University Hospitals NHS Foundation Trust, Oxford, UK. [273]Division of Endocrinology, Department of Pediatrics, Stanford School of Medicine, Stanford University, Stanford, CA, USA. [274]Center for Diabetes Research, Wake Forest School of Medicine, Winston-Salem, NC, USA. [275]Centre for Genetics and Genomics Versus Arthritis, Centre for Musculoskeletal Research, Division of Musculoskeletal and Dermatological Sciences, University of Manchester, Manchester, UK. [276]NIHR Manchester Biomedical Research Centre, Manchester University NHS Foundation Trust, Manchester, UK. [277]Deceased. [278]Present address: Genentech, South San Francisco, CA, USA. [279]Present address: Internal Medicine Research Unit, Pfizer Worldwide Research, Cambridge, MA, USA. [280]Present address: Departamento de Medicina Genómica y Toxicología Ambiental, Instituto de Investigaciones Biomédicas, UNAM, Ciudad de Mexico, Mexico. [281]Present address: The University of Texas Health Science Center at Houston, School of Public Health, Department of Epidemiology, Human Genetics, and Environmental Sciences, Houston, TX, USA. [282]Present address: Institute of Data Science, Korea University, Seoul, South Korea. [283]Present address: Division of Academics, Ochsner Health, New Orleans, LA, USA. [284]Present address: Vertex Pharmaceuticals Ltd, Oxford, UK. [285]Present address: Exeter Centre of Excellence in Diabetes (ExCEeD), Exeter Medical School, University of Exeter, Exeter, UK. [286]Present address: Center for Precision Health Research, National Human Genome Research Institute, National Institutes of Health, Bethesda, MD, USA. [287]Present address: Ibusuki Kozenkai Hospital, Ibusuki, Japan.

[288]Present address: Regeneron Genetics Center, Tarrytown, NY, USA [289]Present address: Institute for Population and Precision Health (IPPH), Biological Sciences Division, The University of Chicago, Chicago, IL, USA. [290]Present address: Clinical Trial Service Unit and Epidemiological Studies Unit, Nuffield Department of Population Health, University of Oxford, Oxford, UK. [291]Present address: Toranomon Hospital, Tokyo, Japan.

Data used in this article were generated by the Diabetes Meta-Analysis of Trans-Ethnic association studies (DIAMANTE) Consortium. The investigators within the DIAMANTE provided data but did not participate in the analysis or writing of this report.

Supplementary Figures and Tables

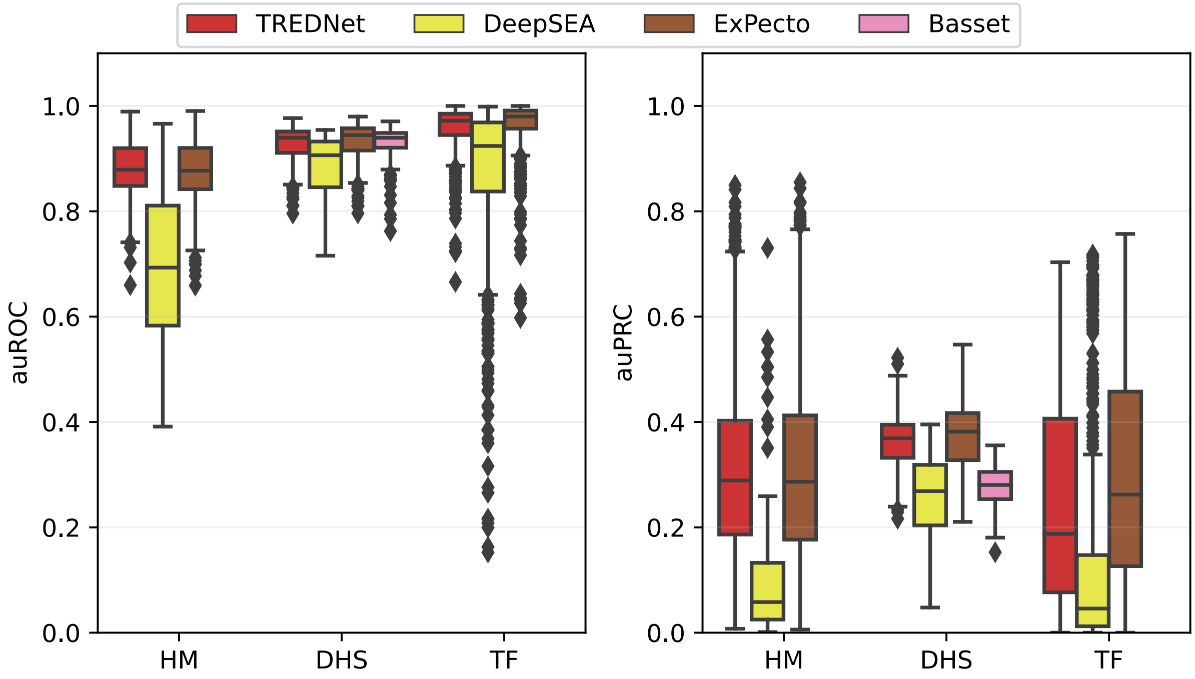

Fig. S1. Characterization of TREDNet phase one. Phase one TREDNet peak prediction accuracy for transcription factors (TFs), histone modifications (HMs), and DNase I hypersensitivity sites (DHSs; x-axis) compared to other models (colors) using area under the receiver operating characteristic (auROC; left) and area under the precision recall curve (auPRC; right) metrics (y-axis).

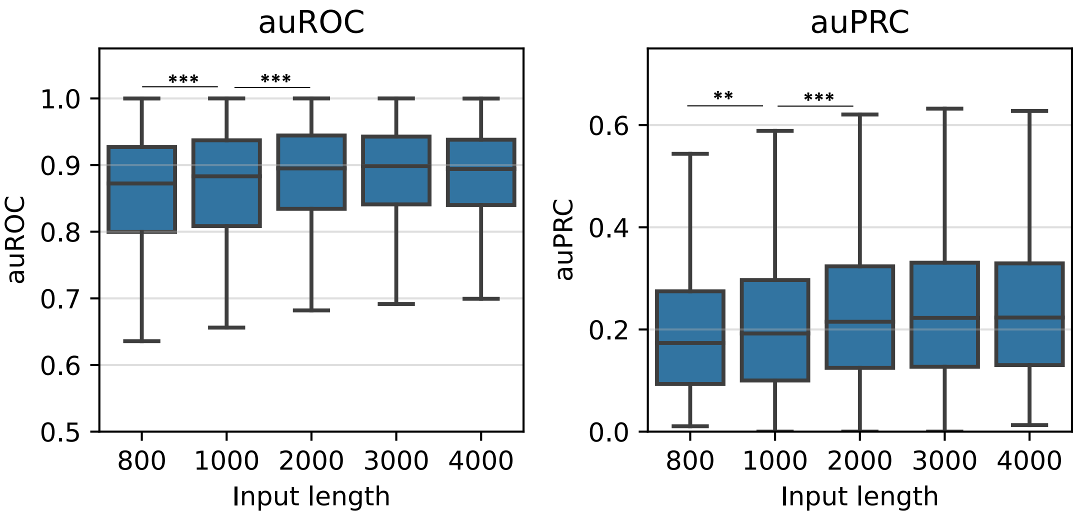

Fig. S2. Optimization of TREDNet phase one DNA sequence window size. TREDNet prediction accuracies for genomic and epigenomic features (e.g., transcription factor binding signals, histone modification signals, DNase I hypersensitivity signals) across different DNA sequence window sizes (x-axis) using area under the receiver operating characteristic (auROC; left) and area under the precision recall curve (auPRC; right) as metrics (y-axis). Stars indicate Wilcoxon rank sum test *P*-values less than various cutoffs: * indicates $P < 0.05$, ** indicates $P < 0.01$, and *** indicates $P < 0.001$.

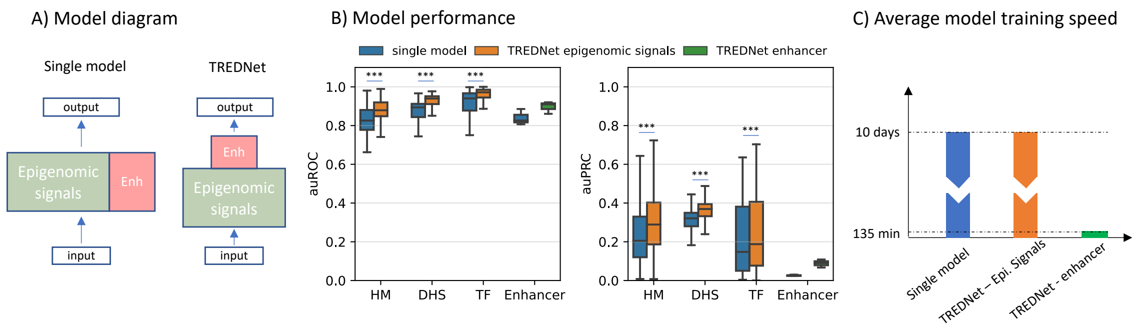

Fig. S3. Comparison of single phase model to TREDNet two phase model. (A) Schematic of a single phase model compared to the two phase TREDNet model. (B) Prediction accuracy (y-axis) of the single phase model (blue), TREDNet phase one model (gold), and TREDNet phase two model (green) across various epigenomic features (x-axis) using area under the receiver operating characteristic (auROC; left) and area under the precision recall curve (auPRC; right) metrics. *** indicates Wilcoxon rank sum test $P < 0.001$. (C) Training speed (y-axis) of each model (x-axis).

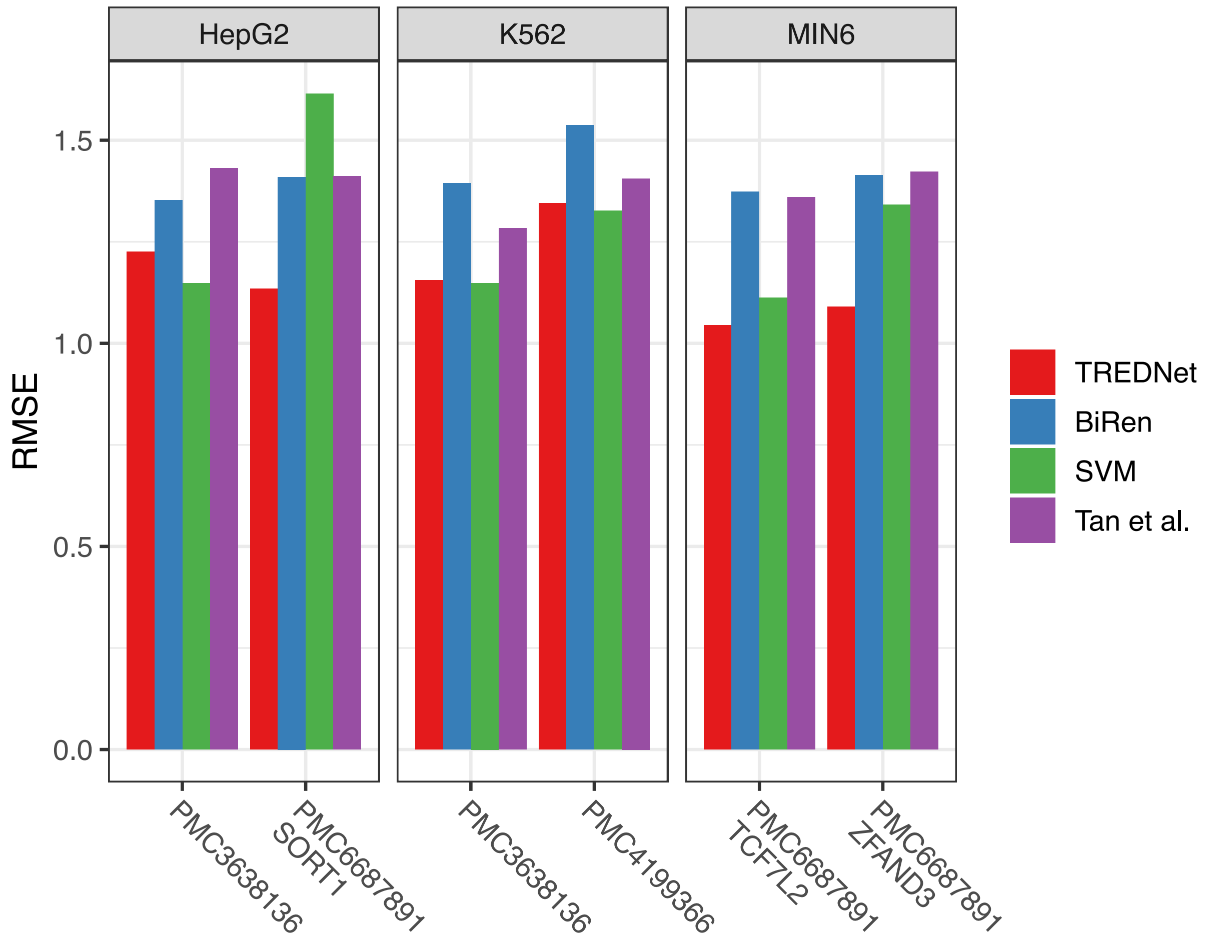

Fig. S4. Extended characterization of TREDNet in MPRA data. Root mean square error (RMSE; y-axis) between predictions of computational methods (colors) and MPRA signals from different experiments (x-axis; coded using PubMed Central identifiers) across biospecimens (facets).

(A) Average enhancer damage and phyloP scores across FOXA binding sites in islet enhancers

(B) Correlation between ED/phyloP scores and information content

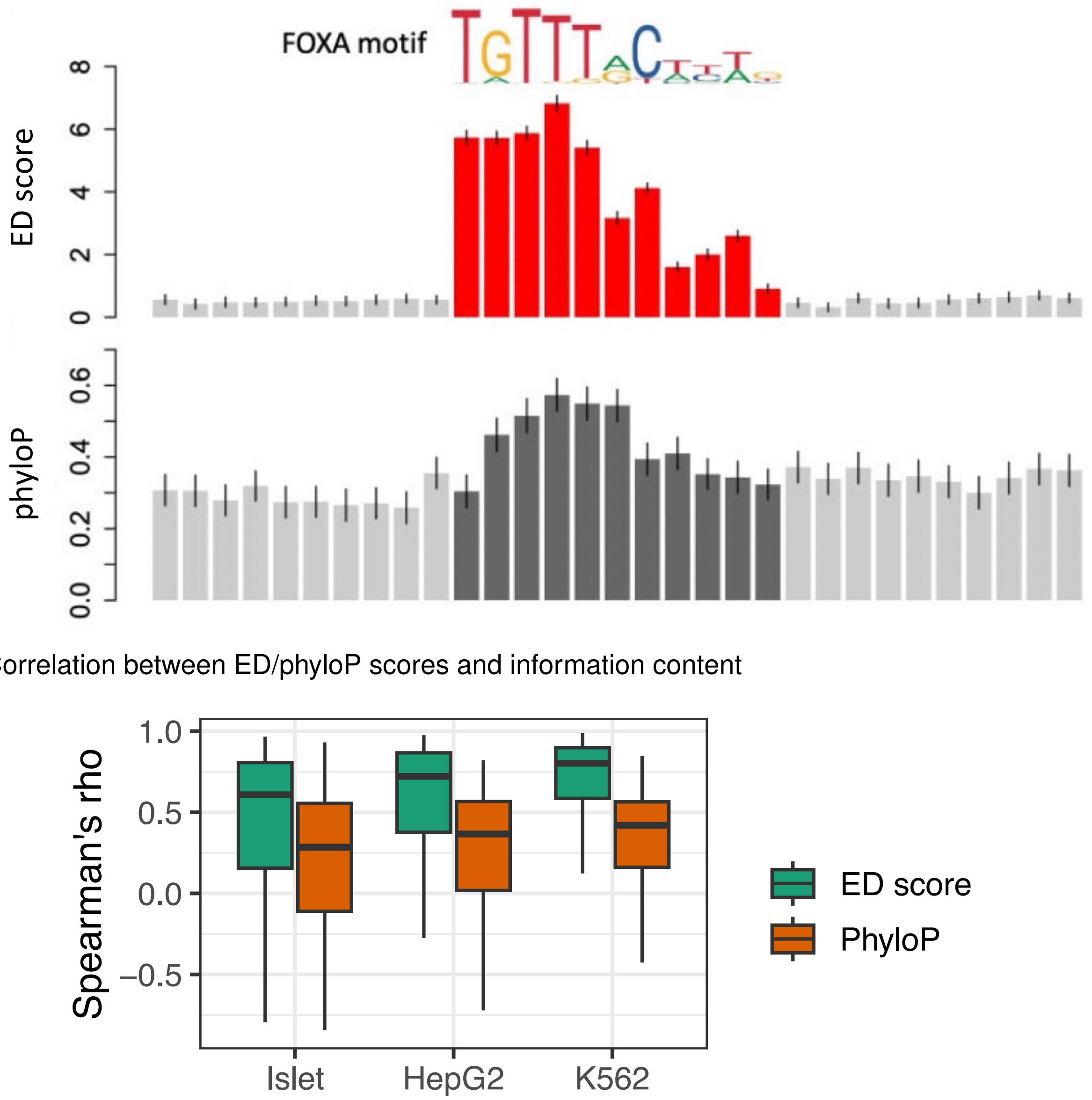

Fig. S5. Comparison of delta scores and motif information content. (A) FOXA position weight matrix (PWM; top panel). The average enhancer damage (ED) score of each position at FOXA transcription factor binding sites (TFBSs), including a 20bp flanking region around the central motif, using the islet TREDNet model (middle panel). The average evolutionary conservation phyloP scores of each position at FOXA TFBSs, including a 20bp flanking region around the central motif (bottom panel). (B) Distribution of correlation coefficients (Spearman's rho; y-axis) between ED/phyloP scores and information content (IC) of each position in PWMs for TFBSs in enhancer regions across biospecimens (x-axis).

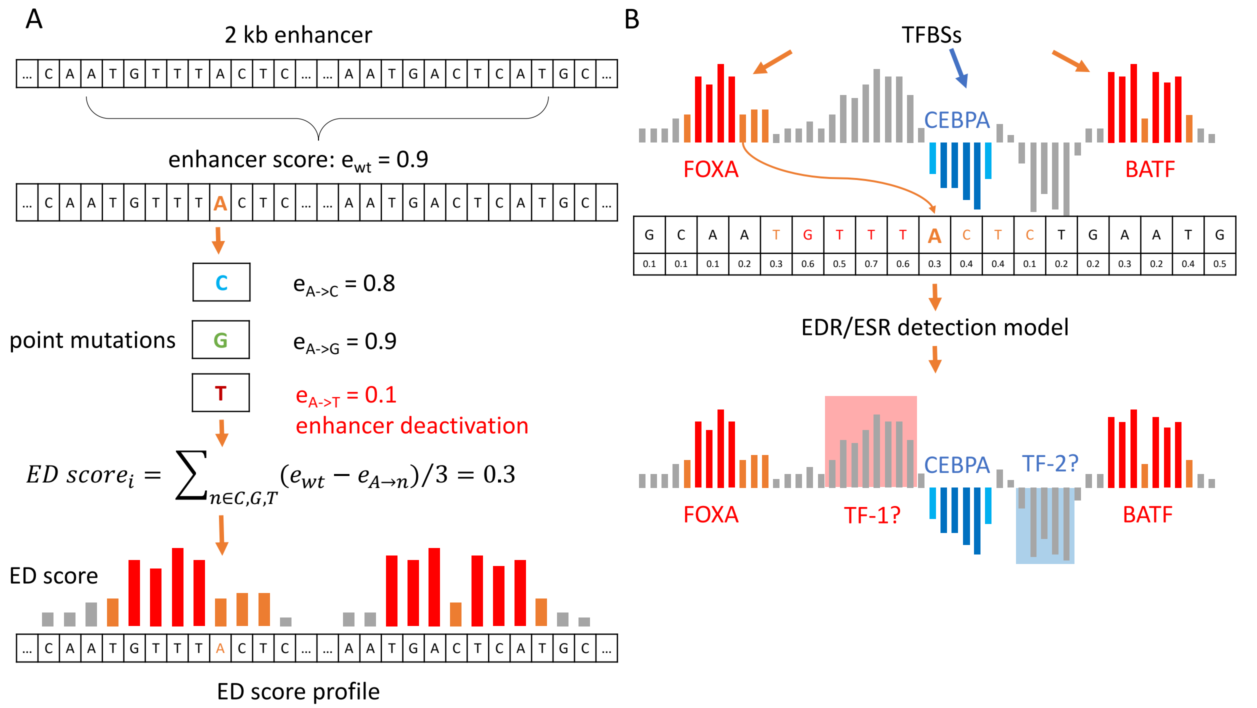

Fig. S6. Detection framework for enhancer damaging and strengthening regions. (A) For each 2kb enhancer region, we calculate the TREDNet enhancer probability score (e) using the "wildtype" GRCh37 reference sequence (e_{wt}). To predict the mutational effect of each nucleotide, we calculate enhancer probability scores for all three non-reference nucleotides at each enhancer position while the remaining enhancer DNA sequence remains unchanged. We generate enhancer damage (ED) score mutational profiles at each sequence position (ED score$_i$) by computing the average difference between the wildtype and allele-specific enhancer scores. (B) We find that known TFBSs correspond to enhancer damaging regions (EDRs; red bars) and enhancer strengthening regions (ESRs; blue bars). To identify known and unknown TFBSs (EDRs/ESRs in gray bars) directly from ED scores, we train a second model to annotate each nucleotide position as an enhancer damaging, enhancer strengthening, or neutral. TF-1 depicts a novel predicted EDR. TF-2 depicts a novel predicted ESR. The ED scores in this plot are for illustrative purposes only.

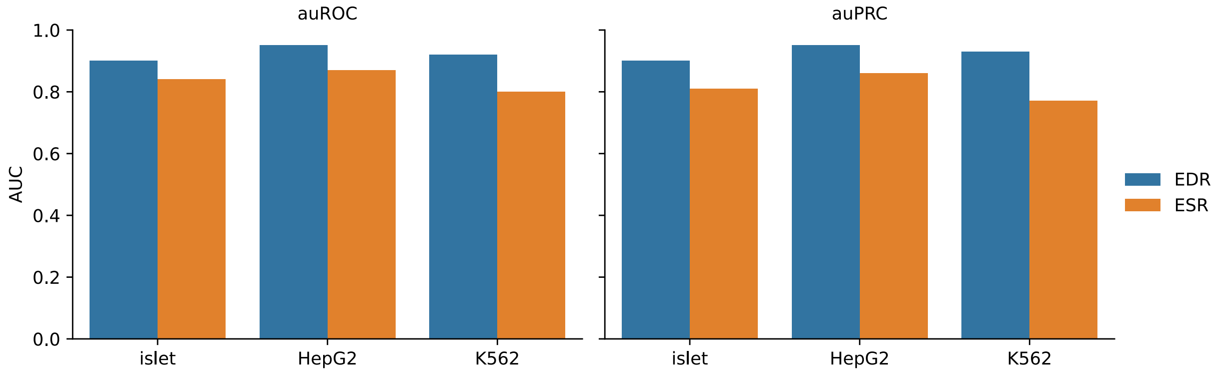

Fig. S7. Performance of EDR and ESR detection models. TFBSs detection performance (y-axis) for EDR and ESR models (colors) for each biospecimen (x-axis) using area under the receiver operating characteristic (auROC; left) and area under the precision recall curve (auPRC; right) metrics.

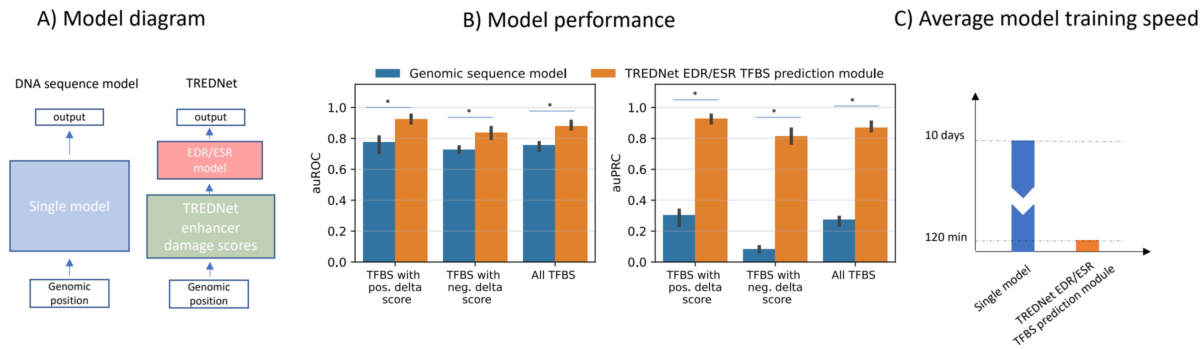

Fig. S8. Prediction of TFBSs based on DNA sequence compared to TREDNet enhancer damage scores. (A) Schematic of the two TFBS prediction models. (B) Accuracy (y-axis) of TFBS prediction models based on DNA sequence (blue) and TREDNet enhancer damage scores (orange) across enhancer damaging and enhancer strengthening TFBSs (x-axis) using area under the receiver operating characteristic (auROC; left) and area under the precision recall curve (auPRC; right) metrics. * indicates Wilcoxon rank sum test $P < 0.05$. (C) Training speed (y-axis) of each model (x-axis).

(A) Example of EDRs and ESRs at a locus

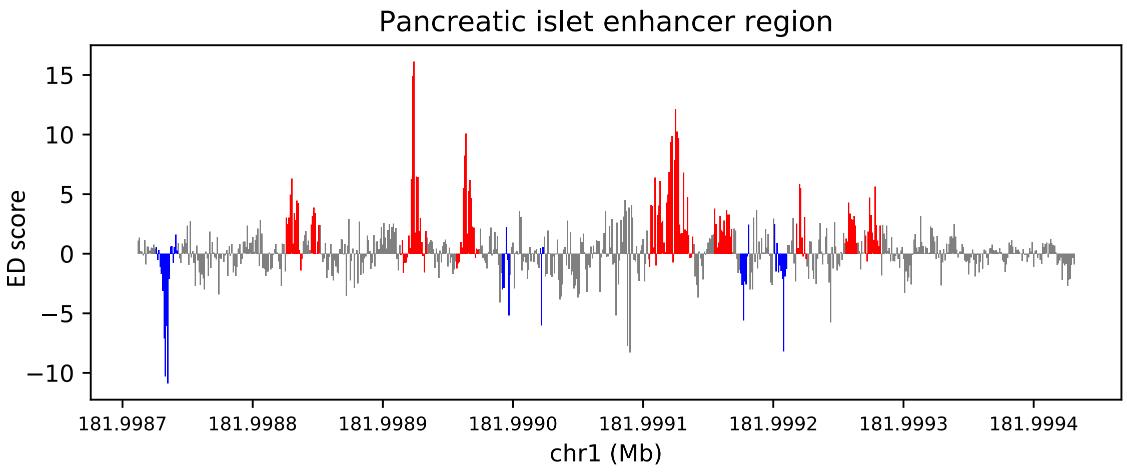

(B) Intersection of EDRs and ESRs

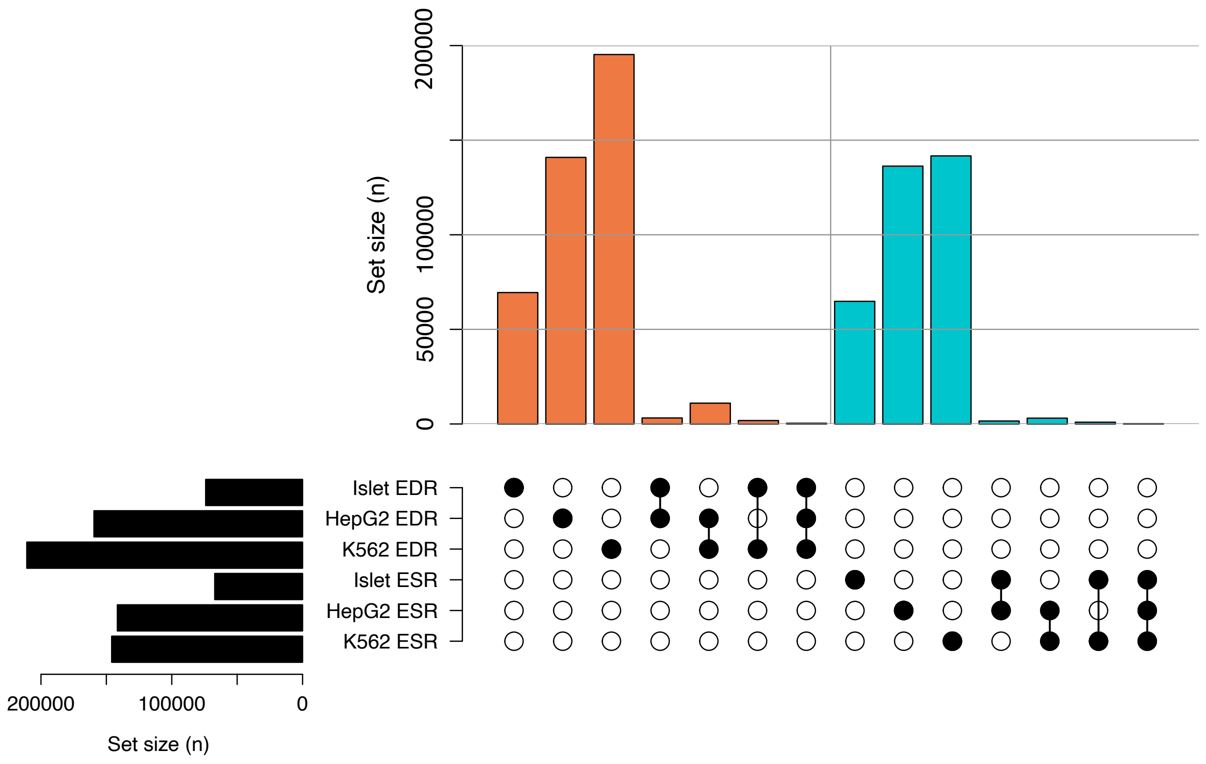

Fig. S9. Enhancer damaging region and enhancer strengthening region results. (A) Example islet enhancer damaging regions (EDRs; blue) and enhancer strengthening regions (ESRs; red) overlaid on the corresponding enhancer damage (ED) scores (y-axis) in an islet enhancer region (x-axis). (B) Comparison of the overlap ($>= 1$ shared bp) of EDRs (orange) and ESRs (blue) across biospecimens, where set groups are shown in bubble plots (bottom).

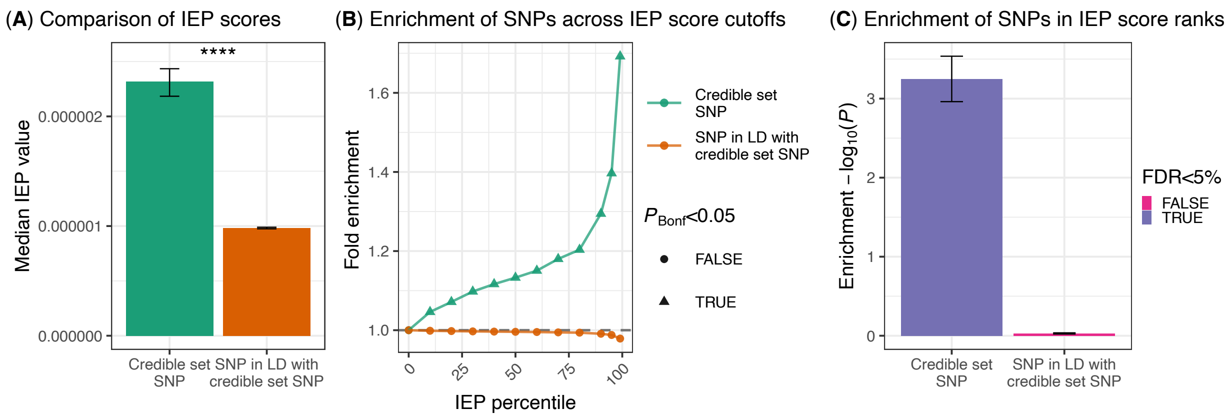

Fig. S10. TREDNet IEP scores at credible set SNPs and SNPs in LD. (A) Comparison of IEP scores (y-axis) of credible set SNPs (green) and SNPs in LD with credible set SNPs (orange; x-axis). Error bars depict 95% confidence intervals of the median. **** indicates Wilcoxon rank sum test $P < 0.0001$. (B) Fold enrichment (y-axis) of credible set SNPs (green) and SNPs in LD with credible set SNPs (orange) at progressive IEP score percentile cutoffs. (C) Enrichment *P*-value (y-axis) from fGSEA analysis of all SNPs ordered by IEP scores. Error bars depict 95% confidence interval of the *P*-value estimate from fGSEA.

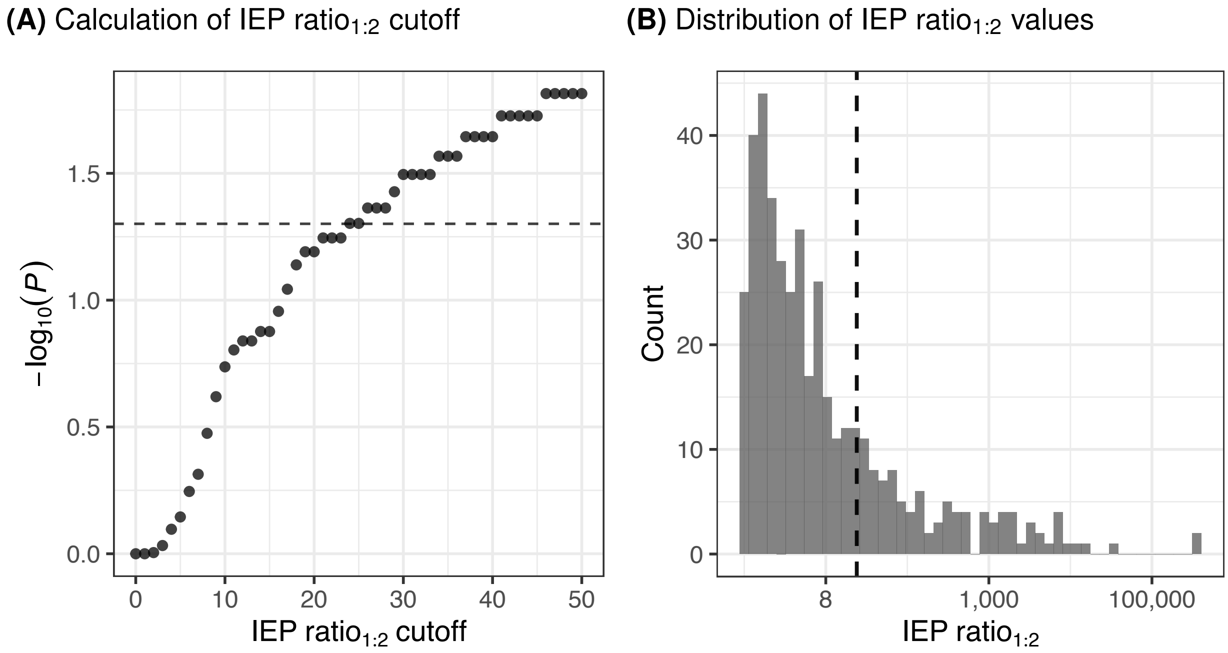

Fig. S11. IEP ratio$_{1:2}$ prioritization cutoff. (A) *P*-values from hypergeometric test (y-axis) evaluating the enrichment of SNPs prioritized by increasing IEP ratio$_{1:2}$ cutoffs (x-axis) from 99% European T2D credible sets in 99% trans-ancestry T2D credible sets at signals where > 1 SNPs are in the 99% European credible set and exactly 1 SNP occurs in the corresponding 99% trans-ancestry credible set. Dashed line at $P = 0.05$. (B) Distribution of IEP ratio$_{1:2}$ values (x-axis) for each signal in the 99% credible set for all disease/traits considered. Dashed line indicates cutoff derived from panel A.

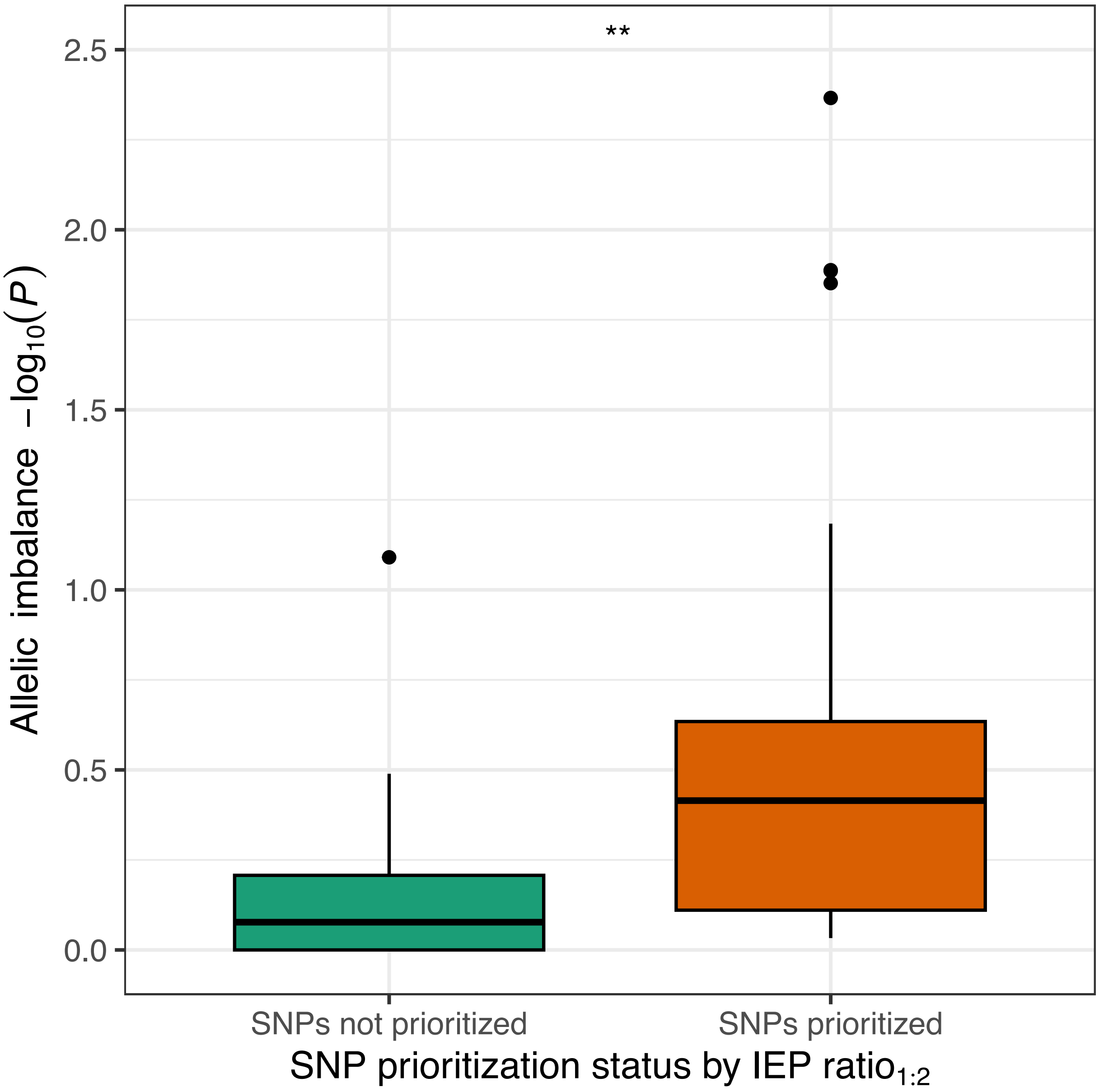

Fig. S12. Allelic imbalance of IEP ratio$_{1:2}$ prioritized SNPs. Comparison of the distribution of allelic imbalance *P*-values (y-axis) from islet ATAC-seq in heterozygous individuals for IEP ratio$_{1:2}$ prioritized SNPs (x-axis; orange) and all other SNPs in the credible set (x-axis; green) at association signals where IEP ratio$_{1:2}$ prioritization identified one candidate causal SNP. ** indicates Wilcoxon rank sum test $P < 0.01$.

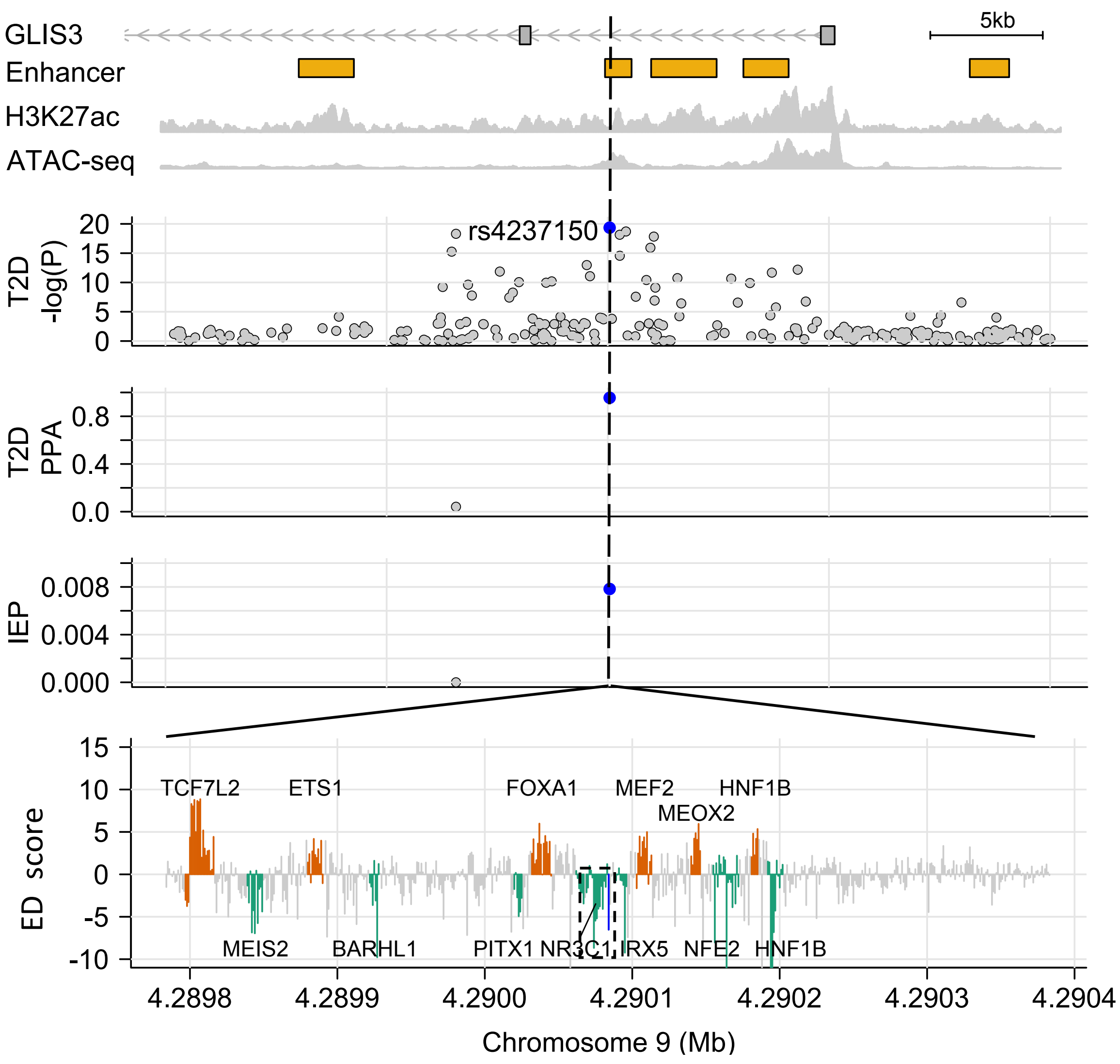

Fig. S13. ***GLIS3* locus.** Locus zoom around the 9:4290085 T2D association (T2D $-log_{10}(P)$ facet) in a *GLIS3* intron. Top facet shows islet enhancers, called from islet H3K27ac ChIP-seq and ATAC-seq data. rs4237150 (blue) is one of two SNPs in the 99% T2D credible set (PPA facet), has a large IEP score (IEP facet), and occurs in an enhancer strengthening region (green; ED score facet). Dashed box indicates the enhancer strengthening region containing the candidate SNP (blue line).

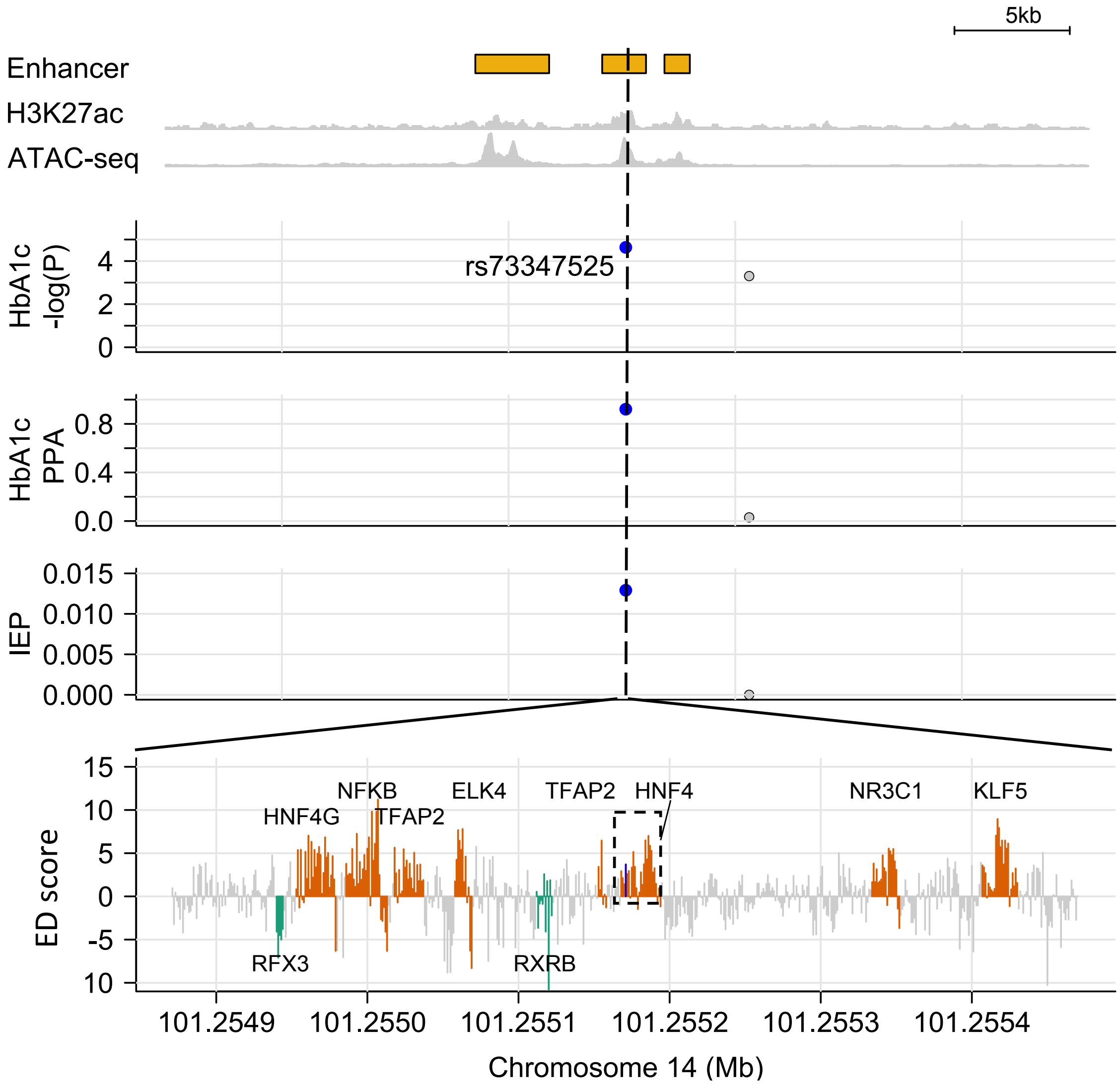

Fig. S14. ***DLK1* locus.** Locus zoom around the 14:101255172 HbA1C association (HbA1C $-log_{10}(P)$ facet), near *DLK1* which is outside of the genomic coordinates shown. Top facet shows islet enhancers, called from islet H3K27ac ChIP-seq and ATAC-seq data. rs73347525 (blue) is one of the 95% HbA1C credible set SNPs (PPA facet), has a large IEP score (IEP facet), and occurs in an enhancer damaging region (orange; ED score facet). Dashed box indicates the enhancer damaging region containing the candidate SNP (blue line).

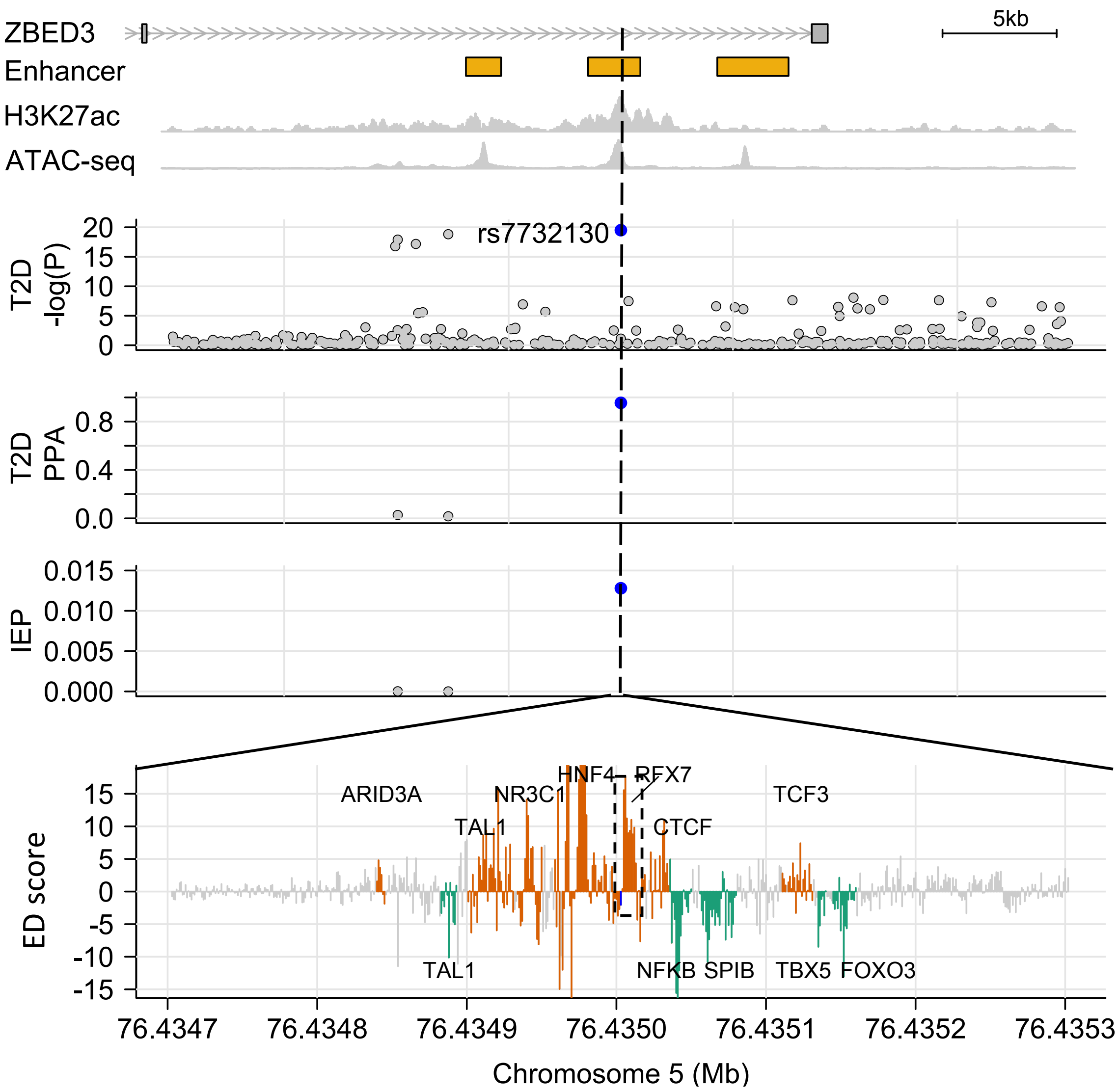

Fig. S15. ***ZBED3* locus.** Locus zoom around the 5:76435004 T2D association (T2D $-log_{10}(P)$ facet), at *ZBED3*. Top facet shows islet enhancers, called from islet H3K27ac ChIP-seq and ATAC-seq data. rs7732130 (blue) is one of the 99% T2D credible set SNPs (PPA facet), has a large IEP score (IEP facet), and occurs in an enhnacer damaging region (orange; ED score facet). Dashed box indicates the enhnacer damaging region containing the candidate SNP (blue line).

TFBS	Average ED score in TFBS motif	Average ED score in TFBS flank	$Ratio_{motif:flank}$
TCF7L2:BACH2:TRIM28:HMGN3	9.0469400	0.690110	13.109400
FOXA:FOX:FOXC2:FOXC1:FOXF2	2.7187200	0.274605	9.900480
CEBPE:CEBPG:CEBPA:CEBPB	5.9105600	0.644939	9.164530
HNF1:HNF1A:HNF1B	1.4904900	0.246813	6.038940
DBP	2.7862300	0.548098	5.083450
MEF2	1.8211600	0.365245	4.986130
NFE2L2	2.8760800	0.623274	4.614470
GATA	1.9332000	0.454534	4.253150
MAFF:MAFG	2.3234800	0.651011	3.569030
TCF21:MYF6:MSC:ASCL2:MYOG	2.0969800	0.594118	3.529570
NFE2	1.8015000	0.522697	3.446550
KLF16	1.3807200	0.407841	3.385440
ATF4	2.4149100	0.717540	3.365540
IRF	1.2652200	0.386515	3.273400
STAT	1.3456300	0.412231	3.264260
TEAD4:TEAD1:TEAD3	1.8860700	0.580423	3.249470
EP300	1.3889300	0.448923	3.093920
MYC	1.5561800	0.506209	3.074180
BCL	1.4166200	0.481807	2.940220
TCF12	1.5869300	0.542909	2.923010
AP1	1.3254800	0.455766	2.908250
MAF	1.4159600	0.490977	2.883960
HDAC2	0.9255450	0.384367	2.407970
TFAP2	1.2330000	0.533990	2.309030
ZNF740	0.8753080	0.384343	2.277410
RXRA	1.0180400	0.448128	2.271760
BHLHA15:OLIG2	0.4721690	0.211981	2.227410
MYF	1.2892200	0.586876	2.196750
TAL1	1.1057300	0.508764	2.173370
NR3C1	0.9831560	0.458071	2.146300
GTF2I	0.8764100	0.409337	2.141050
MXI1	1.1488400	0.548443	2.094730
RFX5	1.0467800	0.499957	2.093740
TFCP2	1.0914500	0.522462	2.089050
CACD	0.9915670	0.482966	2.053080
NRL	1.2814000	0.636737	2.012450
OVOL2	0.8429610	0.454799	1.853480
SREBP	0.9882180	0.533657	1.851780
SPI1	0.8629100	0.468536	1.841720
TATA	0.7920010	0.440804	1.796720
ELF1	0.9690190	0.551094	1.758360
ETS	0.8607650	0.493842	1.743000
EGR1	0.8005320	0.488085	1.640150
ATF3	0.9116230	0.564967	1.613590
CHD2	0.8544780	0.536357	1.593110
E2F	0.8143250	0.516242	1.577410
HMGA1	0.5886910	0.379431	1.551510
SMC3	0.7754170	0.508466	1.525010
ZFX	0.8442480	0.560772	1.505510
SIRT6	0.9468310	0.629131	1.504980
HNF4	0.6240370	0.417979	1.492990

SOX17	0.4695030	0.316569	1.483100
SP1	0.6308790	0.427328	1.476330
HEY1	0.8010530	0.546259	1.466430
SRF	0.7508040	0.515783	1.455660
MZF1	0.5786280	0.407201	1.420990
BHLHE40	0.7330760	0.519300	1.411660
POU2F2	0.6433150	0.456267	1.409950
NANOG	0.6906920	0.493835	1.398630
SIN3A	0.7477220	0.535425	1.396500
PKNOX2:TGIF2LX:TGIF2	0.6049670	0.439624	1.376100
SOX9	0.5003520	0.366266	1.366090
TFCP2L1	0.8484700	0.625706	1.356020
CTCF:RAD21	0.6552790	0.486918	1.345770
REST	0.6843840	0.512284	1.335950
PAX5	0.6703400	0.510149	1.314010
RUNX2	0.6509740	0.498049	1.307050
ESRRA	0.6222210	0.482093	1.290670
REL	0.6279390	0.504035	1.245820
YY1	0.6264430	0.512010	1.223500
ZNF143	0.6017220	0.504918	1.191720
EBF1	0.5783750	0.494279	1.170140
HIC1	0.6316610	0.544796	1.159450
NFKB	0.5295010	0.460065	1.150930
PAX2	0.5726050	0.519630	1.101950
HEY2	0.5197700	0.488618	1.063760
ZIC2:ZIC1:ZIC3	0.5044810	0.487959	1.033860
NKX2-5	0.3991450	0.391284	1.020090
RHOXF1	0.4223190	0.424415	0.995061
GLI	0.4230810	0.425236	0.994932
POU5F1	0.2750300	0.300068	0.916559
SETDB1	0.4876430	0.533604	0.913867
IKZF1	0.4340330	0.476024	0.911788
PBX3	0.4051790	0.473039	0.856545
GCM2:GCM1	0.3609310	0.446948	0.807546
HOXA7	0.2466590	0.308423	0.799743
SOX14	0.1939000	0.324805	0.596974
EN1	0.1833610	0.363579	0.504322
RBPJ	0.1486800	0.358928	0.414233
IKZF2	0.1660080	0.407663	0.407219
SIX5	0.0604405	0.288187	0.209727
ZBTB7C:ZBTB7A	0.0754125	0.439864	0.171445
ZNF410	-0.0111956	0.334417	-0.033478
CUX1	-0.0431143	0.372399	-0.115774
ARID5B	-0.0680515	0.303808	-0.223995
DOBOX4	-0.1748180	0.282215	-0.619450
PBX1	-0.2992370	0.340565	-0.878649
YY2	-0.8091360	0.488708	-1.655660
ONECUT	-0.3668550	0.200166	-1.832750
AP3	-0.5095790	0.141112	-3.611170

Table S1. Prioritization of islet relevant transcription factors using enhancer damage scores. Islet TF footprints ranked by the ratio of the average ED score within the TF footprint motif to the flanking region. TFBS refers to the predicted TF binding site from islet TF footprints. TFs with similar PWMs are merged (Methods) and separated by ":" in the TFBS column.

Disease or trait	Signal	Gene	N SNP	IEP top SNP	IEP 2nd top SNP	IEP score ratio	IEP score top SNP	IEP score 2nd top SNP	IEP percentile top SNP	IEP percentile 2nd top SNP
T2D	chr9:84308948	TLE1	2	rs2796441	rs9410573	Inf	1.13E-02	0.00E+00	0.984	0.000
T2D	chr15:38873115	RASGRP1	2	rs12912777	rs34715063	355601.64	1.94E-03	5.46E-09	0.943	0.058
HbA1c	chr6:33796794-53507100_4	GLP1R	2	rs10305514	rs10305518	37912.17	2.40E-03	6.32E-08	0.949	0.239
HbA1c	chr14:91785258-105440006_4	DLK1	2	rs73347525	rs8004581	16732.11	1.29E-02	7.72E-07	0.986	0.429
HbA1c	chr2:18233271-37277241_2	VIT	2	rs10206462	rs2691106	12031.96	5.54E-04	4.60E-08	0.896	0.213
HbA1c	chr20:22375756-40249273_1	PXMP4	3	rs13042148	rs149142833	9024.43	4.83E-03	5.35E-07	0.967	0.401
Glucose	chr7:40687437-51293550_2	GCK	3	rs2908292	rs2971672	7954.92	3.71E-03	4.66E-07	0.961	0.391
HbA1c	chr5:71716874-85560931_2	ZNF366	3	rs35585881	rs34216626	6931.32	1.07E-03	1.55E-07	0.923	0.309
Glucose	chr14:80252374-99785610_1	FOXN3	2	rs35889227	rs11626777	6383.12	1.36E-02	2.13E-06	0.987	0.506
HbA1c	chr2:36365383-55151959_1	SIX3	3	rs2121564	rs10205222	6262.30	2.78E-04	4.45E-08	0.863	0.210
HbA1c	chr12:38542595-57976118_8	SENP1	2	rs117797076	rs117523200	4999.69	2.10E-05	4.20E-09	0.687	0.048
T2D	chr9:4290085	GLIS3	2	rs4237150	rs1574285	4211.41	7.83E-03	1.86E-06	0.977	0.495
HbA1c	chr4:80561055-96171283_1	ABCG2	8	rs45499402	rs4148155	4041.16	2.38E-02	5.89E-06	0.993	0.585
T2D	chr3:54828827	CACNA2D3	3	rs75088635	rs111494834	3166.90	3.53E-03	1.11E-06	0.959	0.456
HbA1c	chr17:57914080-77759691_4	CCDC47	4	rs75646162	rs72845888	2943.81	1.53E-02	5.19E-06	0.988	0.575
HbA1c	chr3:175462743-186763651_2	ST6GAL1	2	rs6780016	rs3936289	2887.83	4.71E-05	1.63E-08	0.749	0.124
HbA1c	chr11:221870-17419500_10	LMO1	2	rs4758317	rs110420	2339.79	1.31E-02	5.59E-06	0.986	0.581
HbA1c	chr2:161007292-178850138_6	ABCB11	2	rs478333	rs2947987	2154.15	1.14E-05	5.30E-09	0.638	0.057
HbA1c	chr16:7769193-24862718_2	ABCC1	3	rs504348	rs71378214	2066.12	1.12E-03	5.41E-07	0.924	0.402
T2D	chr9:22301092	CDKN2A-CDKN2B	4	rs1575972	rs7045760	1964.45	4.10E-03	2.09E-06	0.963	0.504
HbA1c	chr12:38542595-57976118_7	R3HDM2	4	rs4760278	rs7484541	1811.16	2.60E-03	1.43E-06	0.951	0.475
Fasting glucose	rs17168486	DGKB	2	rs17168486	rs11980500	1553.35	1.21E-03	7.79E-07	0.927	0.429
HbA1c	chr10:3136070-21712524_1	CDC123	4	rs7394200	rs7077792	1433.94	1.96E-05	1.37E-08	0.682	0.111
HbA1c	chr19:360319-10567212_4	ARHGAP45	3	rs12974537	rs35532684	1358.22	1.81E-05	1.33E-08	0.675	0.109
T2D	chr4:6293237	WFS1	36	rs4234731	rs13103357	1324.54	9.24E-03	6.98E-06	0.981	0.599
HbA1c	chr3:4803184-21599247_3	PPARG	2	rs4518111	rs4135247	1268.03	4.45E-05	3.51E-08	0.745	0.189
HbA1c	chr7:36762307-52275656_3	GCK	4	rs2908292	rs2971671	1113.73	3.71E-03	3.33E-06	0.961	0.540
HbA1c	chr14:80124163-99783600_1	FOXN3	3	rs35889227	rs10873398	1047.29	1.36E-02	1.30E-05	0.987	0.649
T2D	chr13:54107583	OLFM4	5	rs12429545	rs4477562	863.72	7.41E-03	8.58E-06	0.976	0.615
Glucose	chr5:67102574-81961482_1	ZBED3	3	rs7732130	rs4457054	797.58	1.28E-02	1.60E-05	0.986	0.666
HbA1c	chr5:67098268-86045601_1	ZBED3	3	rs7732130	rs4457054	797.58	1.28E-02	1.60E-05	0.986	0.666
T2D	chr5:76435004	ZBED3	3	rs7732130	rs4457054	797.58	1.28E-02	1.60E-05	0.986	0.666
HbA1c	chr12:459593-14109457_6	LTBR	3	rs10466905	rs12296430	519.65	9.09E-04	1.75E-06	0.916	0.491
HbA1c	chr1:149784689-167872017_5	OR6Y1	2	rs189857927	rs555280966	501.78	3.09E-05	6.16E-08	0.717	0.237
T2D	chr3:124921457	SLC12A8	3	rs649961	rs569255	480.04	1.94E-02	4.03E-05	0.991	0.738
HbA1c	chr17:945530-13945508_2	PFAS	2	rs2313286	rs4791663	467.81	1.91E-04	4.08E-07	0.842	0.381

HbA1c	chr12:38542595-57976118_3	PFKM	2	rs140895856	rs139395863	445.34	2.12E-04	4.77E-07	0.848	0.393
HbA1c	chr11:2140530-12207149_3	PPFIBP2	16	rs10743026	rs7949659	411.52	3.21E-02	7.81E-05	0.996	0.785
HbA1c	chr3:56856309-74768389_3	SHQ1	3	rs13080180	rs13085136	396.56	1.43E-02	3.61E-05	0.988	0.729
HbA1c	chr2:233963437-241496445_2	ILKAP	4	rs10929270	rs4663862	378.01	3.41E-05	9.02E-08	0.725	0.267
T2D	chr12:4521511	CCND2	7	12:4288000:T:C	12:4301300:C:T	343.43	3.96E-04	1.15E-06	0.880	0.459
HbA1c	chr7:1011402-15893576_1	FAM220A	3	rs13226769	rs35076783	335.44	1.05E-05	3.14E-08	0.632	0.179
HbA1c	chr11:55267156-69977915_4	MYEOV	2	rs148893083	rs188584129	334.73	1.07E-05	3.18E-08	0.633	0.181
HbA1c	chr2:165514409-184356242_2	SCRN3	3	rs10203361	rs7570614	288.39	9.40E-05	3.26E-07	0.798	0.364
HbA1c	chr7:18200482-36754918_3	KIAA0087	15	rs2286177	rs62446872	282.91	1.54E-02	5.45E-05	0.989	0.760
T2D	chr10:114552267	TCF7L2	4	rs10787461	rs941826	232.86	2.34E-02	1.01E-04	0.993	0.802
HbA1c	chr6:125423968-144260129_1	HBS1L	5	rs7776054	rs9389268	214.26	4.71E-05	2.20E-07	0.749	0.335
T2D	chr3:23457080	UBE2E2	6	rs17012829	rs11926494	212.81	2.42E-03	1.14E-05	0.949	0.638
HbA1c	chr17:51634879-67464569_1	YPEL2	2	rs12600858	rs2060779	194.59	5.05E-03	2.60E-05	0.968	0.704
HbA1c	chr5:148867578-168166431_2	TIMD4	11	rs12657266	rs58198139	190.15	6.03E-04	3.17E-06	0.900	0.536
T2D	chr10:114715598	TCF7L2	3	rs2104598	rs11196169	161.36	1.53E-02	9.48E-05	0.989	0.799
HbA1c	chr19:41748220-58664418_2	APOC1	2	rs584007	rs3826688	150.44	2.71E-03	1.80E-05	0.952	0.675
T2D	chr6:40409243	LRFN2	2	rs34298980	rs34045288	146.10	1.29E-03	8.83E-06	0.929	0.618
T2D	chr10:114344288	TCF7L2	2	rs12243296	rs192497142	137.33	4.70E-03	3.42E-05	0.966	0.725
HbA1c	chr17:7672395-27554378_5	PFAS	4	rs10468467	rs9891267	134.12	1.78E-06	1.33E-08	0.492	0.109
Glucose	chr11:400109-17518525_2	PSMA1	4	rs78959242	rs76277448	131.26	4.84E-06	3.69E-08	0.570	0.194
HbA1c	chr17:71006408-81049792_1	FN3K	7	rs7208565	rs12947062	122.32	1.86E-04	1.52E-06	0.841	0.480
HbA1c	chr1:149784689-167872017_2	HORMAD1	2	rs56057831	rs77209899	121.40	1.24E-06	1.03E-08	0.465	0.091
T2D	chr17:36056076	HNF1B	4	rs12938438	rs10962	106.74	7.17E-05	6.71E-07	0.779	0.418
HbA1c	chr1:16516110-33342430_4	RPS6KA1	3	rs4970489	rs4443935	100.87	2.20E-04	2.18E-06	0.850	0.507
HbA1c	chr13:21251235-36045677_3	LINC00426	4	rs1337939	rs1337940	94.26	2.15E-03	2.28E-05	0.946	0.694
T2D	chr2:234191103	ATG16L1-DGKD	4	rs117809958	rs117447187	94.23	1.25E-04	1.33E-06	0.816	0.470
HbA1c	chr9:126208837-140199175_2	GFI1B	2	rs73554552	rs60757417	86.86	1.37E-05	1.58E-07	0.653	0.310
T2D	chr14:79944099	NRXN3	11	rs7156625	rs7144011	81.07	2.02E-02	2.49E-04	0.992	0.857
T2D	chr6:164133001	QKI	7	rs4709746	rs17630640	76.22	3.55E-02	4.66E-04	0.996	0.888
HbA1c	chr17:945530-13945508_3	SMG6	2	rs7213347	rs2873195	69.22	1.47E-05	2.13E-07	0.659	0.332
HbA1c	chr6:100884618-118992323_1	SLC22A16	2	rs72939920	rs12194000	65.60	1.08E-06	1.64E-08	0.454	0.125
T2D	chr10:71466578	VPS26A-NEUROG3	15	rs2616132	rs10762307	64.54	1.84E-02	2.85E-04	0.991	0.864
HbA1c	chr19:36748817-54972918_1	CD33	3	rs34813869	rs1354106	63.33	2.74E-04	4.32E-06	0.862	0.561
T2D	chr6:50788778	TFAP2B	3	rs3798519	rs2206277	59.67	3.57E-04	5.99E-06	0.875	0.587
HbA1c	chr13:107220834-115107493_4	ATP11A	3	rs142329603	rs113245682	57.64	4.90E-04	8.49E-06	0.890	0.614
Glucose	chr11:7117503-23425585_1	PSMA1	3	rs75336838	rs117720468	57.39	1.29E-03	2.25E-05	0.929	0.692
HbA1c	chr11:7598559-21202416_2	PSMA1	5	rs75336838	rs117720468	57.39	1.29E-03	2.25E-05	0.929	0.692
HbA1c	chr2:36863789-52883790_2	FBXO11	34	rs74646951	rs11687989	54.55	1.62E-03	2.97E-05	0.937	0.714
T2D	chr10:122834572	WDR11	3	rs11199755	rs11199753	49.28	7.87E-04	1.60E-05	0.911	0.665

T2D	chr7:127250831	GCC1-PAX4-LEP	3	rs72607746	rs12669223	45.97	4.91E-04	1.07E-05	0.890	0.633
T2D	chr11:128040810	ETS1	14	rs7933438	rs7931773	45.76	1.10E-01	2.41E-03	0.999	0.949
HbA1c	chr17:51634879-67464569_7	YPEL2	9	rs112412433	rs112197279	45.16	2.14E-03	4.74E-05	0.946	0.750
HbA1c	chr5:87068177-103510995_4	GLRX	6	rs2546197	rs12523597	44.93	5.14E-03	1.14E-04	0.969	0.811
Glucose	chr11:365897-11800779_1	KCNQ1	2	rs4930011	rs234864	44.51	5.00E-03	1.12E-04	0.968	0.810
HbA1c	chr16:1484338-20380004_4	ABCC1	2	rs184499898	rs45623833	42.36	3.47E-07	8.19E-09	0.369	0.078
HbA1c	chr3:117101604-133501529_1	SLC12A8	2	rs1416218	rs684030	41.23	6.74E-03	1.64E-04	0.974	0.833
HbA1c	chr3:39480518-57952834_3	CDHR4	3	rs114424909	rs142613277	39.62	2.94E-03	7.43E-05	0.955	0.782
HbA1c	chr19:35485269-54814602_3	SIGLEC5	2	rs73050880	rs112456998	36.33	8.59E-08	2.36E-09	0.264	0.031
Fasting glucose	rs17390909	ELK3	5	rs2268501	rs17331697	36.17	8.59E-03	2.37E-04	0.979	0.854
T2D	chr19:13038415	FARSA-ZNF799	10	rs2974752	rs2242517	35.44	5.59E-03	1.58E-04	0.970	0.831
Glucose	chr10:106575427-121378575_2	TIAL1	8	rs79612474	rs41287142	35.28	1.46E-02	4.13E-04	0.988	0.882
HbA1c	chr8:36842055-49957895_2	ANK1	4	rs72638983	rs72638977	34.54	3.76E-04	1.09E-05	0.878	0.634
HbA1c	chr2:36863789-52883790_4	ZFP36L2	7	rs112694524	rs77552263	33.74	2.09E-03	6.21E-05	0.945	0.769
T2D	chr4:106048291	TET2	4	rs11729069	rs17035289	31.70	1.63E-03	5.15E-05	0.937	0.756
HbA1c	chr3:176710191-196233136_1	ST6GAL1	5	rs1981767	rs9814673	30.62	5.43E-03	1.77E-04	0.970	0.838
HbA1c	chr7:17545963-33138472_2	IGF2BP3	6	rs12700421	rs12700423	29.62	1.64E-02	5.52E-04	0.989	0.896
HbA1c	chr19:41748220-58664418_5	ZC3H4	4	rs62136859	rs1532127	29.42	4.50E-06	1.53E-07	0.564	0.308
Glucose	chr19:36509463-54873286_2	ZC3H4	6	rs62136859	rs1532127	29.42	4.50E-06	1.53E-07	0.564	0.308
T2D	chr3:63897215	PSMD6-ADAMTS9	10	rs6785040	rs2292662	29.13	7.08E-03	2.43E-04	0.975	0.855
HbA1c	chr13:107606835-115109852_1	ATP11A	3	rs76533333	rs12876143	28.84	1.56E-03	5.39E-05	0.936	0.759
HbA1c	chr20:33191665-51625096_3	PLTP	4	rs12185776	rs58847685	28.12	1.30E-04	4.63E-06	0.819	0.566
T2D	chr2:227100490	IRS1	29	rs2943654	rs2673128	27.72	4.97E-03	1.79E-04	0.968	0.838
HbA1c	chr3:134658128-150395262_2	ATP1B3	9	rs11539489	rs55704642	27.52	2.65E-03	9.64E-05	0.952	0.800
T2D	chr3:170724883	SLC2A2	9	rs1905505	rs6804915	25.75	7.94E-04	3.08E-05	0.911	0.717
T2D	chr11:2856658	INS-IGF2-KCNQ1	2	rs4930011	rs2237895	24.97	5.00E-03	2.00E-04	0.968	0.845

Table S2. Signals where IEP ratio$_{1:2}$ refined 95/99% credible set SNPs to one SNP. Signals with a single SNP identified by IEP ratio$_{1:2}$ that previously had > 1 SNPs in the 95% or 99% credible set, depending on the study (see Methods). Gene column refers to the nearest gene except in the case of the T2D data, for which we used the gene reported by the original study. N SNP column refers to the number of SNPs in the original 95/99% credible set (uniform prior). Note: IEP percentiles are rounded.

Number	SNP	Ref	Alt	High score allele	Ref score	Alt score	EDR/ESR FPR 5%	EDR/ESR FPR 10%	TF motif overlap	Increased binding allele	Log difference	Motif database
1	rs4970489	T	C	T	0.028478	0.020762	ESR islet	ESR islet				
								ESR K562	Gli2(Zf)	C	3.096273503	HOMER
								ESR HepG2	GLI3(Zf)	C	2.835239687	HOMER
									ESRRA_disc3	C	2.513480022	ENCODE
									GLIS3(Zf)	C	2.26659149	HOMER
									ZIC3_1	C	2.240891524	ENCODE
									GLI2_1	C	2.148906	ENCODE
2	rs56057831	A	T	A	0.002942	0.002519	ESR islet	ESR islet				
							ESR K562	ESR K562	STAT_disc2	A	3.105875853	ENCODE
							ESR HepG2	ESR HepG2	PRDM1_disc2	A	3.084881004	ENCODE
									AP1_disc3	A	3.075774981	ENCODE
									FOSL2::JUND	A	2.481396335	JASPAR
									Fos(bZIP)	A	2.143113679	HOMER
									JUN(var.2)	A	2.058555651	JASPAR
									FOS::JUN	A	2.020873188	JASPAR
3	rs189857927	A	G	G	0.030573	0.031552	ESR K562	ESR K562				
							ESR HepG2	ESR HepG2	FOXB1_3	G	1.732971395	ENCODE
4	rs7394200	T	C	T	0.017668	0.016557		ESR islet				
								ESR K562	IRF_known1	T	2.972475411	ENCODE
								ESR HepG2	IRF_known3	T	2.642484247	ENCODE
									IRF_known2	T	2.610411732	ENCODE
									IRF_known21	T	2.574518808	ENCODE
5	rs2616132	G	A	G	0.284545	0.219826	ESR islet	ESR islet				
								ESR HepG2	RFX3	G	2.69704899	JASPAR
								ESR K562	TOPORS_1	G	2.405515474	ENCODE
6	rs12243296	A	G	G	0.186022	0.208544	ESR islet	ESR islet				
								ESR HepG2	ZNF317(Zf)	G	3.732861917	HOMER
								ESR K562	MEIS2_4	G	2.496597974	ENCODE
									MEIS3_5	G	2.47248413	ENCODE
									SNAI1	G	2.467928313	JASPAR
									MEIS3_3	G	2.458357833	ENCODE
									TBX21_6	G	2.417424353	ENCODE
									ZEB2(Zf)	G	2.381653616	HOMER
									ZSCAN16_1	G	2.283253636	ENCODE
									SNAI2_1	G	2.004775326	ENCODE
7	rs10787461	A	G	A	0.42461	0.369475	EDR islet	EDR islet				
							EDR HepG2	EDR K562	MYF_1	G	3.407552374	ENCODE
								EDR HepG2	Sox17(HMG)	A	3.2988296	HOMER
									Oct4:Sox17(POU,Homeobox,HMG)	A	3.252885479	HOMER
									FOXJ3_3	G	2.850901326	ENCODE
									FOXJ1_1	A	2.706519979	ENCODE
									SOX17_4	A	2.670871198	ENCODE
									FOXJ3_6	G	2.622625904	ENCODE

									SOX18_2	A	2.510067804	ENCODE
									SOX21_2	A	2.455707495	ENCODE
									SOX3_1	A	2.44392421	ENCODE
									SOX10_8	A	2.372716917	ENCODE
									SOX4_2	A	2.349837978	ENCODE
									FOXJ2_3	G	2.330394372	ENCODE
									SOX10_3	A	2.324207557	ENCODE
									SOX2_2	A	2.317605595	ENCODE
									SOX8_4	A	2.302585093	ENCODE
									SOX7_3	A	2.302585093	ENCODE
									SOX8_7	A	2.272345208	ENCODE
									SOX9_7	A	2.225081532	ENCODE
									Sox11	A	2.196891854	JASPAR
									SOX11_2	A	2.195191761	ENCODE
									ZNF136	A	2.140857844	JASPAR
									SRY_5	A	2.119535999	ENCODE
									SOX15_2	A	2.100241416	ENCODE
8	rs2104598	G	A	G	0.805454	0.78646		EDR islet				
								EDR HepG2	HOXA13_4	A	3.220457687	ENCODE
									NKX6-3	A	2.531218137	JASPAR
									NFATC1_1	G	2.072509105	ENCODE
9	rs79612474	C	T	C	0.63966	0.616856	EDR islet	EDR islet				
								ESR K562	PITX2_2	C	3.810769272	ENCODE
								ESR HepG2	PITX1_3	C	3.337827768	ENCODE
									Mecom	C	3.325248486	JASPAR
									Otx2(Homeobox)	C	3.268637334	HOMER
									RUNX1_8	C	3.251411615	ENCODE
									DOBOX5_1	C	3.248302826	ENCODE
									OTX2_3	C	3.181834553	ENCODE
									POU2F2_known9	T	3.091042453	ENCODE
									FOXP3_1	T	2.643212682	ENCODE
									GSC_1	C	2.48365743	ENCODE
									GSC(Homeobox)	C	2.292174357	HOMER
									FOXD3_1	T	2.170815815	ENCODE
									SIX5_disc3	T	2.062791529	ENCODE
									FOXD3_2	T	2.008878906	ENCODE
10	rs11199755	C	T	C	0.185268	0.181022		EDR islet				
									KLF3(Zf)	C	3.246373074	HOMER
									Sp2(Zf)	C	2.75565966	HOMER
									EGR1_known12	C	2.525059524	ENCODE
									RREB1_2	C	2.492938821	ENCODE
									RREB1	C	2.489732279	JASPAR
									SP2	C	2.455789251	JASPAR
									CCNT2_disc2	C	2.365042448	ENCODE
									RREB1_1	C	2.339323919	ENCODE
11	rs4930011	C	G	C	0.156832	0.124978	ESR islet	EDR HepG2				

								ESR islet	EGR1_disc7	C	4.028531344	ENCODE
									ZSCAN4	C	3.803162311	JASPAR
									PBX3_disc3	G	3.250943961	ENCODE
									EGR1	C	3.026358931	JASPAR
									KLF9	C	2.947315174	JASPAR
									EGR1_known12	C	2.873433242	ENCODE
									EGR1_known9	C	2.81979424	ENCODE
									ZSCAN4_3	C	2.817213003	ENCODE
									EGR3_3	C	2.780620894	ENCODE
									EGR3_1	C	2.7120445	ENCODE
									KLF9	C	2.590267165	JASPAR
									RREB1	C	2.547940028	JASPAR
									RREB1_2	C	2.541477001	ENCODE
									EGR3_2	C	2.479482059	ENCODE
									EGR4_2	C	2.47940911	ENCODE
									EGR4	C	2.459888887	JASPAR
									FOXA1:AR(Forkhead,NR)	G	2.364698918	HOMER
									RREB1	C	2.254323635	JASPAR
									RREB1_2	C	2.249265455	ENCODE
12	rs10743026	C	G	G	0.386128	0.456547	ESR islet	ESR islet				
							ESR K562	ESR K562	FOXG1_1	G	1.976288184	ENCODE
13	rs4758317	C	A	C	0.413518	0.381916	ESR HepG2	ESR HepG2				
									OSR2	C	5.370790015	JASPAR
									SPDEF_4	A	2.157954807	ENCODE
14	rs78959242	T	C	C	0.02426	0.024458		EDR islet				
								EDR HepG2	GATA1	T	5.60199978	JASPAR
15	rs75336838	C	T	T	0.13511	0.144061	EDR HepG2	EDR HepG2				
							ESR islet	ESR islet	NFAT(RHD)	T	4.860563982	HOMER
								ESR K562	ZNF75D	C	3.734136046	JASPAR
									SIX5_disc2	C	3.698448905	ENCODE
									ZNF143_disc1	C	3.569078254	ENCODE
									RBPJ_1	C	3.215123475	ENCODE
									Sox11	T	2.839302131	JASPAR
									SOX18_2	T	2.834275855	ENCODE
									NFAT5	T	2.741135926	JASPAR
									SOX10_8	T	2.699206176	ENCODE
									SOX7_3	T	2.676595249	ENCODE
									SOX2_2	T	2.586226756	ENCODE
									SOX21_2	T	2.577251797	ENCODE
									SOX11_2	T	2.571497063	ENCODE
									SOX21	T	2.530741186	JASPAR
									SRY_5	T	2.508437147	ENCODE
									SOX8_4	T	2.497424868	ENCODE
									GFY(?)	C	2.486277452	HOMER
									SOX9_7	T	2.407255716	ENCODE
									SOX17_4	T	2.401256621	ENCODE

									RBPJ_2	C	2.381458073	ENCODE
									SOX3_1	T	2.363209715	ENCODE
									SOX4_2	T	2.160614832	ENCODE
									NFAT:AP1(RHD,bZIP)	T	2.156733216	HOMER
									THAP11	C	2.137070655	JASPAR
									SOX10_3	T	2.11971991	ENCODE
									SOX8_7	T	2.10650472	ENCODE
									SRY_5	T	2.095210034	ENCODE
									SOX2_2	T	2.029291758	ENCODE
16	rs148893083	C	T	C	0.016996	0.016369	ESR HepG2	ESR HepG2				
									HNF4a(NR),DR1	C	2.277325426	HOMER
17	rs7933438	G	A	G	0.639608	0.467173	EDR islet	EDR islet				
							EDR K562	EDR K562	GATA_disc2	G	2.816530844	ENCODE
							EDR HepG2	EDR HepG2	Fos(bZIP)	G	2.092289684	HOMER
18	12:4288000:T:C	T	C	C	0.094517	0.098531		EDR K562				
									ZKSCAN3_1	C	2.254693805	ENCODE
19	rs10466905	G	A	G	0.036248	0.011166	EDR islet	EDR islet				
							EDR K562	EDR K562	DUX4	A	5.801974501	JASPAR
							EDR HepG2	EDR HepG2	AP1_known1	G	5.020181906	ENCODE
									AP1_known4	G	4.652335654	ENCODE
									DUXA	A	4.531350274	JASPAR
									DUXA_1	A	4.440470231	ENCODE
									DUX4(Homeobox)	A	4.251664817	HOMER
									AP1_known2	G	4.251187034	ENCODE
									AP1_known3	G	3.718860286	ENCODE
									Duxbl(Homeobox)	A	3.322416504	HOMER
									POU6F2_2	A	3.143652404	ENCODE
									GATA_disc6	G	3.111493064	ENCODE
									PHOX2B_2	A	2.822506282	ENCODE
									POU6F2_2	A	2.792133318	ENCODE
									ALX4_3	A	2.112372219	ENCODE
									ALX4_4	A	2.047692843	ENCODE
20	rs117797076	G	A	G	0.036711	0.036139		ESR HepG2				
									T_2	A	3.21237526	ENCODE
									Tbx6(T-box)	A	2.75605942	HOMER
									CTCF_disc6	A	2.620420204	ENCODE
									Tbr1(T-box)	A	2.320143405	HOMER
									TBR1_1	A	2.00847213	ENCODE
21	rs140895856	C	T	T	0.081348	0.083881	EDR islet	EDR islet				
								EDR K562	RHOXF1_2	T	3.824351967	ENCODE
								EDR HepG2	RHOXF1_1	T	3.501563238	ENCODE
									CHD2_disc2	T	3.088776591	ENCODE
									E2F_disc5	T	3.065831034	ENCODE
22	rs4760278	C	A	C	0.123359	0.102314	EDR islet	EDR islet				
							EDR K562	EDR K562	ATF3_known3	C	2.417075403	ENCODE
							EDR HepG2	EDR HepG2	SOX21_5	A	2.353522029	ENCODE

									SOX10_10	A	2.037250319	ENCODE
23	rs2268501	C	T	C	0.815791	0.805267	ESR HepG2	ESR islet				
								ESR HepG2	HIF2a(bHLH)	T	3.013505873	HOMER
								ESR K562	NFAT:AP1(RHD,bZIP)	C	2.134998196	HOMER
24	rs1337939	T	C	T	0.480531	0.476055						
									IRF_known18	C	2.011388078	ENCODE
25	rs12429545	G	A	A	0.352101	0.372017		EDR islet				
								ESR K562	MYC_disc6	G	3.139323193	ENCODE
26	rs76533333	A	G	G	0.094495	0.108793		ESR islet				
									HNF4_known24	G	2.744417845	ENCODE
									NKX2-1_1	G	2.717018871	ENCODE
									NR2F6_1	G	2.516159193	ENCODE
									SCRT1	G	2.481508176	JASPAR
									NR2F6_4	G	2.375655661	ENCODE
									VDR_1	G	2.259765096	ENCODE
27	rs142329603	T	C	C	0.444308	0.445407		EDR K562				
									ZNF341	T	5.191345247	JASPAR
									ZNF341(Zf)	T	4.234796398	HOMER
									ZNF189(Zf)	T	3.772879429	HOMER
									Znf263(Zf)	C	3.525672293	HOMER
									GRE(NR),IR3	T	3.154337304	HOMER
									GRE(NR),IR3	T	3.111436306	HOMER
									PAX4_5	T	2.995732274	ENCODE
									NR3C1_known9	T	2.975461309	ENCODE
									GRE(NR),IR3	T	2.967891648	HOMER
									NR3C1_known16	T	2.741461887	ENCODE
									ARE(NR)	T	2.732360791	HOMER
									NR3C1_disc1	T	2.697000365	ENCODE
									Ar	T	2.670309873	JASPAR
									PR(NR)	T	2.669516537	HOMER
									NR3C1_known16	T	2.662977513	ENCODE
									PR(NR)	T	2.654154433	HOMER
									ARE(NR)	T	2.653636779	HOMER
									Ar	T	2.604327151	JASPAR
									GRE(NR),IR3	T	2.42022056	HOMER
									NR3C2	T	2.402610969	JASPAR
28	rs7156625	G	A	A	0.441799	0.483507	EDR islet	EDR islet				
							EDR HepG2	EDR HepG2	SIX5_disc2	G	3.490603969	ENCODE
							ESR K562	ESR K562	ZNF143_disc1	G	3.409818125	ENCODE
									CTCFL_disc1	G	3.157423046	ENCODE
									RXRA_disc2	G	2.709382646	ENCODE
									CTCF(Zf)	G	2.317781177	HOMER
									HNF1_2	A	2.08133779	ENCODE
29	rs35889227	G	T	G	0.255772	0.202556	EDR islet	EDR islet				
							EDR HepG2	EDR HepG2	SOX17_5	G	3.075069016	ENCODE
								ESR K562	SOX7_4	G	3.056819966	ENCODE

									SOX10_10	G	2.96686429	ENCODE
									SOX21_5	G	2.946048853	ENCODE
									SOX10_6	G	2.907721396	ENCODE
									SOX9_9	G	2.890371758	ENCODE
									SOX1_3	G	2.851692903	ENCODE
									SOX3_3	G	2.774396807	ENCODE
									SOX2_3	G	2.72861418	ENCODE
									SOX8_3	G	2.721987709	ENCODE
									SOX18_4	G	2.705095989	ENCODE
									SOX8_8	G	2.697687414	ENCODE
									SOX2_7	G	2.679452794	ENCODE
									SOX14_3	G	2.604587219	ENCODE
									LHX6_5	T	2.583298496	ENCODE
									LHX6_5	T	2.409448239	ENCODE
									FOXA_known1	T	2.343686769	ENCODE
30	rs73347525	A	G	A	0.429225	0.399129	EDR islet	EDR islet				
									ZNF415(Zf)	G	4.65646348	HOMER
									HNF4_disc4	G	3.359350272	ENCODE
									ZBTB12	A	2.884779945	JASPAR
31	rs12912777	C	T	C	0.293417	0.286795		ESR islet				
									Bcl6(Zf)	T	2.324824705	HOMER
									ATF3_known5	C	2.141097623	ENCODE
32	rs504348	C	G	G	0.121522	0.130112		EDR HepG2				
									ESRRA_known8	C	2.20255224	ENCODE
33	rs184499898	A	G	G	0.005922	0.00598						
									RREB1	G	2.708050201	JASPAR
									RREB1_2	G	2.703552136	ENCODE
									RREB1_1	G	2.510224458	ENCODE
34	rs7213347	G	C	C	0.016265	0.017125						
									Sox7(HMG)	C	4.115967026	HOMER
									Sox4(HMG)	C	3.388512222	HOMER
									Sox17(HMG)	C	3.129652998	HOMER
									Sox6(HMG)	C	2.934890614	HOMER
									SOX9_3	C	2.805484651	ENCODE
									SOX8	C	2.770016925	JASPAR
									SOX9_1	C	2.60359262	ENCODE
									SOX9_2	C	2.145852101	ENCODE
									SOX9	C	2.145852101	JASPAR
35	rs10468467	A	G	A	0.003669	0.003184	ESR islet	ESR islet				
							ESR K562	ESR K562	PROX1_1	G	2.864134743	ENCODE
							ESR HepG2	ESR HepG2	PROX1	G	2.823392085	JASPAR
36	rs2313286	C	G	G	0.019141	0.026371	EDR K562	EDR K562				
							ESR islet	ESR islet	Usf2(bHLH)	C	2.211969757	HOMER
37	rs12938438	C	G	G	0.03663	0.038492	ESR HepG2	ESR HepG2				
									AMYB(HTH)	G	5.386983004	HOMER
									MYB_5	G	3.27512537	ENCODE

38	rs112412433	C	T	T	0.718152	0.721123		EDR islet				
									TCF7_2	C	2.30965226	ENCODE
39	rs12600858	G	A	A	0.218999	0.24005	EDR HepG2	EDR islet				
								EDR K562	GLI2_2	G	2.415913778	ENCODE
								EDR HepG2	RUNX2_2	A	2.380215254	ENCODE
40	rs75646162	G	A	G	0.265144	0.207517	EDR islet	EDR islet				
							EDR K562	EDR K562	BPTF_1	A	3.654898446	ENCODE
41	rs7208565	C	T	C	0.032429	0.02668	EDR HepG2	EDR HepG2				
									KLF17	C	3.299829401	JASPAR
42	rs12974537	C	T	C	0.011863	0.010339	ESR K562	ESR islet				
								ESR K562	Reverb(NR),DR2	T	3.379995174	HOMER
									REST_disc9	C	3.028414921	ENCODE
43	rs2974752	G	A	G	0.214312	0.188236	EDR islet	EDR islet				
								EDR K562	NRF1_disc1	G	3.300101265	ENCODE
								EDR HepG2	HES2	G	3.17073545	JASPAR
									HES2	G	2.988512026	JASPAR
									MYC_known20	G	2.957991946	ENCODE
									MYC_known20	G	2.75705778	ENCODE
									HEY2_2	G	2.642652301	ENCODE
									HEY2	G	2.574598837	JASPAR
									EGR1_disc3	G	2.523296539	ENCODE
									HES7	G	2.349976881	JASPAR
									SOHLH2	G	2.130115833	JASPAR
44	rs584007	A	G	A	0.205884	0.192706	ESR K562	EDR islet				
								EDR HepG2	CPEB1_1	A	2.624126199	ENCODE
								ESR K562	ZFP3(Zf)	G	2.361425593	HOMER
45	rs62136859	A	G	A	0.014113	0.013794	ESR K562	EDR islet				
							ESR HepG2	ESR K562	TBX20_5	A	2.469546606	ENCODE
46	rs34813869	A	G	G	0.081391	0.084626	EDR K562	ESR HepG2				
									TLX2_1	A	2.654890833	ENCODE
									ZNF165(Zf)	G	2.342237865	HOMER
									VENTX_2	A	2.335758867	ENCODE
									ZSCAN16_1	G	2.311228189	ENCODE
									VENTX_2	A	2.268968482	ENCODE
									EGR1_disc4	G	2.243808657	ENCODE
									TFAP4_1	A	2.113067898	ENCODE
47	rs73050880	A	G	G	0.001908	0.001952						
									RBPJ:Ebox(?,bHLH)	G	1.511212886	HOMER
48	rs10206462	C	T	T	0.07925	0.08571		EDR islet				
									BATF_disc2	C	3.587696667	ENCODE
									IRF_disc6	C	3.164196796	ENCODE
									PRDM1_known1	T	2.682074715	ENCODE
									NFKB_known6	T	2.582650982	ENCODE
									ZNF684	C	2.255225623	JASPAR
									NFkB-p65(RHD)	T	2.15910337	HOMER
49	rs112694524	G	A	G	0.822565	0.820019	EDR islet	EDR islet				

								EDR K562	SIN3A_disc4	A	3.065659288	ENCODE
									MYF_1	A	2.97134082	ENCODE
									TATA_disc7	G	2.374990937	ENCODE
									REST_known3	A	2.253794929	ENCODE
									TFAP2_known4	G	2.152772815	ENCODE
									HIC1_1	G	2.118628595	ENCODE
									RAD21_disc8	G	2.086361985	ENCODE
									TFAP4_1	A	2.032188657	ENCODE
50	rs2121564	C	G	G	0.028745	0.036395	ESR islet	ESR islet				
							ESR K562	ESR K562	CREB3L2_2	G	3.925575972	ENCODE
							ESR HepG2	ESR HepG2	CREB3L1_3	G	3.721624535	ENCODE
									Plagl1	G	3.559028949	JASPAR
									CREB3L2_2	G	3.435499976	ENCODE
									CREB3L1_3	G	3.389221191	ENCODE
									SP1_known3	G	3.013353875	ENCODE
									MYC_known14	G	2.856470206	ENCODE
									CREB3L1_4	G	2.732969523	ENCODE
									HIC1_3	C	2.470644203	ENCODE
									ATF3_disc3	C	2.065309659	ENCODE
51	rs74646951	C	A	C	0.07839	0.05773	EDR islet	EDR islet				
							EDR K562	EDR K562	GATA5	C	4.651690202	JASPAR
							EDR HepG2	EDR HepG2	GATA_disc1	C	4.05862648	ENCODE
									Gata4(Zf)	C	3.513224589	HOMER
									HDAC2_disc1	C	2.630758527	ENCODE
									Gata6(Zf)	C	2.605635196	HOMER
									Gata1(Zf)	C	2.321816455	HOMER
									Gata2(Zf)	C	2.210893735	HOMER
									GATA4	C	2.186374561	JASPAR
									GATA_known15	C	2.18311144	ENCODE
									GATA_known21	C	2.088099604	ENCODE
									GATA_known17	C	2.071473372	ENCODE
									GATA_known22	C	2.007615807	ENCODE
52	rs478333	G	A	G	0.013148	0.012279	ESR islet	ESR islet				
							ESR K562	ESR K562	E2A(bHLH),near_PU.1	G	3.966417077	HOMER
							ESR HepG2	ESR HepG2	TCF3	G	3.356745619	JASPAR
									TCF12(var.2)	G	3.298502646	JASPAR
									Slug(Zf)	G	3.242532021	HOMER
									SNAI1	G	2.455092387	JASPAR
									ZEB1	G	2.31261024	JASPAR
									SNAI3	G	2.301348234	JASPAR
53	rs10203361	G	A	A	0.03134	0.034098	ESR islet	ESR islet				
							ESR K562	ESR K562	SP1_known9	G	2.949716036	ENCODE
							ESR HepG2	ESR HepG2	KLF17	G	2.710630848	JASPAR
									SREBP_known2	G	2.644555132	ENCODE
									KLF16_1	G	2.530800506	ENCODE
									Srebp2(bHLH)	G	2.492441669	HOMER

									KLF16	G	2.445393496	JASPAR
									SP8_1	G	2.307099773	ENCODE
									SP8	G	2.270099638	JASPAR
									SP3	G	2.008077055	JASPAR
54	rs2943654	C	T	T	0.194378	0.217248						
									HMBOX1_1	C	1.941571747	ENCODE
55	rs117809958	T	A	T	0.05081	0.04835	EDR islet	EDR islet				
								EDR HepG2	MAF_known6	A	6.466508178	ENCODE
									MAF_known8	A	5.91162166	ENCODE
									Mafb	A	4.69245297	JASPAR
									MAF_known10	A	4.618115351	ENCODE
									MafF(bZIP)	A	4.598676657	HOMER
									NRL_1	A	4.173632838	ENCODE
									MAFG	A	3.976167127	JASPAR
									MAFF	A	3.888855946	JASPAR
									Tbx6(T-box)	A	3.786202832	HOMER
									TBX6	A	3.556720745	JASPAR
									TBX3	A	3.498615044	JASPAR
									MAF_known4	A	3.299637182	ENCODE
									MAF_known5	A	3.150419866	ENCODE
									MafA(bZIP)	A	3.031420003	HOMER
									SIX5_known5	A	2.730858897	ENCODE
									TBX18	A	2.723128092	JASPAR
									TBX4_1	A	2.637845942	ENCODE
									TBX4	A	2.637845942	JASPAR
									Tbx5(T-box)	A	2.368398312	HOMER
									MAFF_1	A	2.196886797	ENCODE
									TBX5_4	A	2.056402751	ENCODE
									TBX5	A	2.056402751	JASPAR
56	rs10929270	T	A	T	0.015716	0.013546	ESR K562	EDR islet				
							ESR HepG2	ESR K562	ESR1	T	3.390225762	JASPAR
								ESR HepG2	ETS:RUNX(ETS,Runt)	A	2.8963598	HOMER
									ESRRA_known6	T	2.754570217	ENCODE
									STAT_disc7	A	2.616045056	ENCODE
									ESRRA_known6	T	2.412210965	ENCODE
									ZNF460	T	2.240179527	JASPAR
									TATA_disc8	T	2.114706563	ENCODE
57	rs13042148	C	T	C	0.124361	0.08552	EDR islet	EDR islet				
							EDR K562	EDR K562	SP1_known8	C	6.758909651	ENCODE
							EDR HepG2	EDR HepG2	SP9	C	6.502290171	JASPAR
									KLF14	C	6.06828664	JASPAR
									SP4	C	5.984591668	JASPAR
									KLF14_1	C	5.529098707	ENCODE
									KLF11	C	5.476836637	JASPAR
									SP3	C	5.368698163	JASPAR
									KLF7_1	C	5.302069716	ENCODE

SP4_1	C	5.271565181	ENCODE
KLF13	C	5.168724538	JASPAR
SP8	C	4.980329945	JASPAR
SP8_1	C	4.938439003	ENCODE
KLF16	C	4.875659858	JASPAR
KLF13_1	C	4.851005149	ENCODE
KLF16_1	C	4.774358743	ENCODE
SP1_known9	C	4.714696585	ENCODE
KLF2	C	4.682589785	JASPAR
KLF6	C	4.510791249	JASPAR
KLF10	C	4.352404379	JASPAR
HNF4_known6	T	4.211291827	ENCODE
KLF9	C	4.077781873	JASPAR
SP1	C	4.040855877	JASPAR
SP4_2	C	4.016226783	ENCODE
KLF1(Zf)	C	3.950174771	HOMER
Klf9(Zf)	C	3.912023005	HOMER
KLF14(Zf)	C	3.781512256	HOMER
KLF4_1	C	3.735305551	ENCODE
Sp5(Zf)	C	3.708073901	HOMER
KLF10(Zf)	C	3.686307657	HOMER
SP1_known2	C	3.515342727	ENCODE
KLF12_2	C	3.477011753	ENCODE
Klf12	C	3.409744442	JASPAR
KLF3(Zf)	C	3.401566453	HOMER
SP1_known6	C	3.390919799	ENCODE
KLF6(Zf)	C	3.3437798	HOMER
Sp1(Zf)	C	3.293711467	HOMER
Klf4(Zf)	C	3.280360909	HOMER
KLF5(Zf)	C	3.267450495	HOMER
SP1_known5	C	3.175783682	ENCODE
KLF17	C	3.152966996	JASPAR
SP1_known4	C	3.018004909	ENCODE
IRF_disc4	C	2.916144836	ENCODE
KLF3	C	2.86277444	JASPAR
SP2_disc3	C	2.788280052	ENCODE
Sp2(Zf)	C	2.776417699	HOMER
HIC1_4	T	2.553899521	ENCODE
RXRB_2	T	2.490763182	ENCODE
PAX9_1	T	2.473928173	ENCODE
PAX5(Paired,Homeobox)	T	2.451823615	HOMER
RXRA_known14	T	2.346617363	ENCODE
RXRG_1	T	2.33408376	ENCODE
Rxra	T	2.3067692	JASPAR
RXRA_known12	T	2.281751006	ENCODE
PAX9	T	2.243161673	JASPAR

									RXRB_1	T	2.217085784	ENCODE
									NR2F6_3	T	2.215336327	ENCODE
									Nr2f6	T	2.205421345	JASPAR
									TR4(NR),DR1	T	2.188268265	HOMER
									RXRG	T	2.183039942	JASPAR
									RXRA_known10	T	2.170866937	ENCODE
									HNF4_known19	T	2.145447107	ENCODE
									PPARA_1	T	2.135763783	ENCODE
									NR2F6_2	T	2.129832987	ENCODE
									Egr2(Zf)	C	2.073355096	HOMER
									RXRB	T	2.045274118	JASPAR
									HNF4_known23	T	2.039642627	ENCODE
									PPARD	T	2.001380995	JASPAR
58	rs12185776	C	G	C	0.069386	0.067508	ESR K562	ESR K562				
									NKX3-1_4	G	2.330684004	ENCODE
59	rs4518111	A	C	C	0.050576	0.051441		EDR islet				
								ESR HepG2	ZNF384	A	5.981414211	JASPAR
									ZNF384	A	5.611667186	JASPAR
									HDAC2_disc6	A	5.515454894	ENCODE
									HDAC2_disc6	A	5.176804814	ENCODE
									HDAC2_disc6	A	5.118227954	ENCODE
									ZNF35_1	A	4.823651724	ENCODE
									HDAC2_disc6	A	4.297098158	ENCODE
									FOXJ3_8	A	3.432895757	ENCODE
									HDAC2_disc6	A	3.328330512	ENCODE
									STAT1::STAT2	C	3.174350746	JASPAR
									Foxd3	C	3.045535096	JASPAR
									FOXD3_2	C	3.041669369	ENCODE
									FOXD3_1	C	3.018873802	ENCODE
									FoxD3(forkhead)	C	2.736481868	HOMER
									FOXJ3_3	A	2.722578302	ENCODE
									ZNF384	A	2.647678073	JASPAR
									FOXJ3_8	A	2.515605637	ENCODE
									FOXJ3_4	C	2.5060676	ENCODE
									HDAC2_disc6	A	2.387955464	ENCODE
									FOXJ2_3	A	2.308691982	ENCODE
									PAX5_known1	C	2.263307317	ENCODE
									FOXJ3_6	A	2.248682875	ENCODE
									FOXP1_1	A	2.138135661	ENCODE
60	rs17012829	C	T	T	0.149219	0.163974		ESR islet				
								ESR K562	NR3C1_known6	C	2.785059846	ENCODE
								ESR HepG2	SIX5_disc3	T	2.009482953	ENCODE
61	rs114424909	C	G	G	0.123447	0.143895	ESR islet	ESR islet				
							ESR HepG2	ESR HepG2	T1ISRE(IRF)	G	3.317315898	HOMER
									IRF7	G	2.903864438	JASPAR
									IRF_known17	G	2.828158573	ENCODE

									IRF_known1	G	2.757840866	ENCODE
									Sox3	G	2.342858992	JASPAR
62	rs75088635	A	G	G	0.159955	0.179606		ESR islet				
								ESR K562	GCM1_2	A	2.356887249	ENCODE
63	rs6785040	T	C	C	0.409713	0.42633	ESR K562	ESR HepG2				
									ZNF143_disc4	C	4.426528486	ENCODE
									AP1_disc10	C	4.154163057	ENCODE
									REST_disc5	C	3.988624399	ENCODE
									YY2	C	3.502755611	JASPAR
									BCL_disc9	C	3.423358953	ENCODE
									YY1_disc5	C	2.893341122	ENCODE
									YY1(Zf)	T	2.758006299	HOMER
									NR3C1_disc4	T	2.467664843	ENCODE
									EGR1_disc6	C	2.178766801	ENCODE
									YY2_2	C	2.081278089	ENCODE
									E2F_disc8	C	2.054804237	ENCODE
									E2F_disc7	C	2.039595633	ENCODE
									ZFP42	T	2.031032402	JASPAR
64	rs13080180	C	T	C	0.317879	0.272845	EDR islet	EDR islet				
							EDR K562	EDR K562	FOXA_known2	T	3.039407337	ENCODE
							EDR HepG2	EDR HepG2	FOXC1_1	C	2.968562368	ENCODE
									FOXJ3_6	T	2.410381663	ENCODE
									FOXA_known3	T	2.31833345	ENCODE
									FOXJ3_8	T	2.273764654	ENCODE
									NFATC2_1	T	2.169233832	ENCODE
									NFATC2	T	2.169233832	JASPAR
65	rs1416218	C	G	G	0.187612	0.218471						
									GLIS3(Zf)	C	4.035155691	HOMER
									Zfp809(Zf)	G	3.647155611	HOMER
									p53(p53)	G	3.516772491	HOMER
									ZNF528	C	3.437565026	JASPAR
									ZNF460	C	2.550087088	JASPAR
									ZNF768(Zf)	C	2.442004628	HOMER
									TFCP2L1_1	G	2.327156354	ENCODE
									Zfp809(Zf)	G	2.176726056	HOMER
66	rs649961	T	C	C	0.212595	0.28138		EDR islet				
								EDR K562	HNF4_disc3	T	3.069989044	ENCODE
									TCF21_1	C	2.949310437	ENCODE
67	rs11539489	C	G	G	0.38198	0.388803		ESR K562				
									Sp2(Zf)	G	2.376693065	HOMER
68	rs1905505	G	A	A	0.058743	0.070076	ESR islet	ESR islet				
								ESR HepG2	TFCP2_1	G	2.94284281	ENCODE
69	rs1981767	G	A	G	0.306473	0.288754	EDR islet	EDR islet				
								EDR K562	YY1_known3	G	2.675152498	ENCODE
70	rs6780016	C	T	C	0.02834	0.026677	EDR K562	EDR HepG2				
									KLF17	T	3.047502294	JASPAR

									FOXO4_5	T	2.505784235	ENCODE
									HIC1_1	C	2.360572351	ENCODE
71	rs4234731	A	G	A	0.383202	0.359077	ESR islet	ESR islet				
							ESR HepG2	ESR K562	GLIS3(Zf)	A	2.211251815	HOMER
								ESR HepG2	CTCF-SatelliteElement(Zf?)	A	2.151624991	HOMER
									GLIS2_1	A	2.039363318	ENCODE
72	rs45499402	G	C	G	0.212494	0.100503	EDR islet	EDR islet				
							EDR K562	EDR K562	ZNF281_1	G	5.411986535	ENCODE
							EDR HepG2	EDR HepG2	SP1_disc3	G	5.045183102	ENCODE
									ZNF740_1	G	4.881527109	ENCODE
									EGR1_known3	G	4.837124367	ENCODE
									ZNF281_1	G	4.733003557	ENCODE
									CCNT2_disc2	G	4.670309488	ENCODE
									SP2	G	3.811257558	JASPAR
									ZNF219_1	G	3.700713912	ENCODE
									GTF2I_1	G	3.658941366	ENCODE
									KLF16	G	3.654908097	JASPAR
									KLF16_1	G	3.621908828	ENCODE
									SPZ1_1	G	3.571562728	ENCODE
									HF1H3B_1	G	3.344524995	ENCODE
									ZNF740_4	G	3.311585223	ENCODE
									CACBP_1	G	3.284180923	ENCODE
									Zfp281(Zf)	G	3.277038129	HOMER
									ZNF740_2	G	3.228064251	ENCODE
									KLF14(Zf)	G	3.22444687	HOMER
									GLIS1	G	3.138194955	JASPAR
									ZNF740	G	3.003279479	JASPAR
									GLIS3_1	G	2.977040141	ENCODE
									Zfp281(Zf)	G	2.961902409	HOMER
									SP4_1	G	2.936643357	ENCODE
									ZNF740	G	2.917828604	JASPAR
									WT1_1	G	2.737109128	ENCODE
									HF1H3B_1	G	2.688863717	ENCODE
									ZNF143_disc3	G	2.621160191	ENCODE
									SP1_known7	G	2.607406675	ENCODE
									Klf9(Zf)	G	2.538365228	HOMER
									GLIS1_1	G	2.512989619	ENCODE
									SP4_2	G	2.489171049	ENCODE
									SP1_known8	G	2.467977588	ENCODE
									MZF1_2	G	2.457906048	ENCODE
									Znf281	G	2.438508259	JASPAR
									RREB1_1	G	2.436864709	ENCODE
									KLF14_1	G	2.43299311	ENCODE
									SP9	G	2.267630078	JASPAR
									KLF14	G	2.241271908	JASPAR
									SP4	G	2.235574383	JASPAR

									SP3	G	2.13470422	JASPAR
									SP1_known9	G	2.068970242	ENCODE
									SP8_1	G	2.065130027	ENCODE
									ZNF281_1	G	2.049749752	ENCODE
									SP8	G	2.042349999	JASPAR
									KLF11	G	2.034857635	JASPAR
									ZNF740_3	G	2.029387953	ENCODE
									ZNF740_1	G	2.022795721	ENCODE
73	rs11729069	C	G	G	0.192851	0.200973		ESR K562				
									RARG_8	G	2.30982276	ENCODE
74	rs35585881	G	A	A	0.174187	0.180135						
									TGIF1_2	A	1.312706004	ENCODE
75	rs7732130	G	A	G	0.444827	0.416061	EDR islet	EDR islet				
							ESR HepG2	ESR K562	ZNF143	G	3.817234429	JASPAR
								ESR HepG2	ZNF143_known1	G	3.215915783	ENCODE
									RFX7_1	G	2.860913051	ENCODE
									ZNF143_known2	G	2.8479427	ENCODE
76	rs2546197	T	G	G	0.212669	0.234596		ESR islet				
								ESR HepG2	PAX1_1	G	1.487728259	ENCODE
77	rs12657266	C	T	C	0.083453	0.076229	EDR HepG2	EDR islet				
								EDR HepG2	MYOD1_1	T	3.049106734	ENCODE
									BHLHE40_disc1	T	2.786011743	ENCODE
									SIRT6_disc1	T	2.363769782	ENCODE
									TCF4	T	2.149455342	JASPAR
									TFAP2C(var.2)	C	2.13905145	JASPAR
									TFAP2A	C	2.122096717	JASPAR
78	rs10305514	G	T	G	0.155389	0.139972	ESR K562	ESR K562				
							ESR HepG2	ESR HepG2	ZNF684	G	2.505322706	JASPAR
79	rs34298980	T	C	C	0.230408	0.23588	ESR islet	ESR islet				
							ESR K562	ESR K562	SMAD4_1	T	2.567738052	ENCODE
							ESR HepG2	ESR HepG2	HINFP_3	C	2.051944292	ENCODE
80	rs3798519	A	C	C	0.044694	0.051619		ESR islet				
								ESR HepG2	RARA::RXRA	C	3.784446406	JASPAR
									RXRA_known8	C	3.525374169	ENCODE
81	rs72939920	A	T	A	0.003721	0.003431	EDR islet	EDR islet				
							ESR K562	ESR K562	Smad4	T	1.69813421	JASPAR
82	rs7776054	A	G	A	0.036979	0.035704	ESR HepG2	ESR HepG2				
									SOX7_2	A	2.799758628	ENCODE
									SOX21_3	A	2.677972746	ENCODE
									SOX21_3	A	2.594721516	ENCODE
									SRY_6	A	2.46010467	ENCODE
									SOX7_2	A	2.454474607	ENCODE
									SRY_6	A	2.443406919	ENCODE
									SOX8_2	A	2.035455393	ENCODE
									SOX9_4	A	2.010689954	ENCODE
83	rs4709746	C	T	C	0.49737	0.425999		EDR islet				

								EDR HepG2	Ptf1a(var.3)	T	5.861995448	JASPAR
									MYOD1_3	T	5.578304817	ENCODE
									Rbpjl	T	5.103892788	JASPAR
									ZEB2(Zf)	T	4.756937074	HOMER
									ZEB1_known3	T	4.382026635	ENCODE
									TCF4_1	T	3.899068166	ENCODE
									TCF3_2	T	3.620112597	ENCODE
									EGR4_1	C	3.547508501	ENCODE
									MYF6_1	T	3.497001511	ENCODE
									SNAI3	T	3.491632247	JASPAR
									EGR3_1	C	3.362597208	ENCODE
									ID4_1	T	3.23044881	ENCODE
									TCF3_1	T	3.140914283	ENCODE
									Slug(Zf)	T	3.062275664	HOMER
									EGR1_disc1	C	2.978844056	ENCODE
									Snail1(Zf)	T	2.967909768	HOMER
									SNAI2	T	2.863880142	JASPAR
									MYOD1	T	2.773189503	JASPAR
									E2A(bHLH),near_PU.1	T	2.691695313	HOMER
									EGR4_2	C	2.633679658	ENCODE
									EGR4	C	2.631939862	JASPAR
									TBX5_3	T	2.591454109	ENCODE
									TBX5_4	T	2.556389795	ENCODE
									TBX5	T	2.556389795	JASPAR
									EGR1_known10	C	2.548810452	ENCODE
									EGR3_3	C	2.530594834	ENCODE
									EGR1_known1	C	2.478924578	ENCODE
									EGR2	C	2.435481766	JASPAR
									EGR1_known8	C	2.419622138	ENCODE
									EGR1_known7	C	2.28460259	ENCODE
									EGR1_known11	C	2.276439813	ENCODE
									MYF6_2	T	2.273451943	ENCODE
									GRHL2	T	2.26371094	JASPAR
									EGR3_2	C	2.112639611	ENCODE
									EGR1_known6	C	2.085854757	ENCODE
									TBX15_2	T	2.070552594	ENCODE
									EGR1_known2	C	2.062612238	ENCODE
84	rs13226769	C	G	G		0.00487	0.006493	ESR islet	ESR islet			
							ESR K562	ESR K562	TRIM28_disc2	C	3.169803931	ENCODE
							ESR HepG2	ESR HepG2	KLF13_1	C	2.997555428	ENCODE
									ZFP42	C	2.38253633	JASPAR
									SMAD_1	C	2.020464532	ENCODE
85	rs17168486	C	T	T		0.161831	0.168991	ESR islet	ESR islet			
							ESR HepG2	ESR K562	RFX3_4	T	2.391780672	ENCODE
								ESR HepG2	RFX3	T	2.313821166	JASPAR
									MYC_disc4	T	2.268937059	ENCODE

									RFX2_1	T	2.248242352	ENCODE
									RFX2_1	T	2.243195293	ENCODE
									RFX2_3	T	2.170467128	ENCODE
									RFX2_3	T	2.132934596	ENCODE
									RFX2	T	2.069573041	JASPAR
									RFX2	T	2.062686815	JASPAR
									RFX3_2	T	2.001244272	ENCODE
86	rs12700421	C	G	C	0.325961	0.27579	EDR islet	EDR islet				
									FOXP3_1	C	3.111651376	ENCODE
									TP63	G	2.190265195	JASPAR
									TP73_1	G	2.127179628	ENCODE
									RAD21_disc6	C	2.041529496	ENCODE
									TP73	G	2.008911391	JASPAR
87	rs2286177	T	C	T	0.370513	0.328905	ESR K562	EDR islet				
								ESR K562	NR2F2_1	C	2.600419537	ENCODE
									RFX3_1	T	2.598172345	ENCODE
									RFX5_known5	T	2.493781254	ENCODE
									EGR1_disc4	C	2.230553965	ENCODE
									RFX1	T	2.009952515	JASPAR
88	rs2908292	C	T	C	0.153968	0.129866	ESR HepG2	EDR islet				
								EDR K562	RUNX1_2	T	2.257927942	ENCODE
89	rs72607746	C	T	T	0.123945	0.127785	ESR islet	ESR HepG2				
							ESR K562		GFI1B_1	T	4.089466504	ENCODE
90	rs72638983	G	T	G	0.039249	0.029665	ESR HepG2	EDR islet				
								EDR K562	IRF_disc1	G	3.685112971	ENCODE
								EDR HepG2	E2F_disc4	G	2.940565892	ENCODE
									ZNF423_1	G	2.441171256	ENCODE
									RFX5_disc2	G	2.283936534	ENCODE
									CEBPB_disc2	G	2.126908079	ENCODE
									NFY_known6	G	2.106036415	ENCODE
									NFY_known3	G	2.067937717	ENCODE
91	rs4237150	G	C	C	0.096983	0.149395	ESR islet	ESR islet				
							ESR HepG2	ESR HepG2	NR3C1_known7	G	3.450522448	ENCODE
									ZNF528(Zf)	G	2.312585176	HOMER
92	rs1575972	T	A	A	0.225602	0.242512	ESR islet	ESR islet				
							ESR HepG2	ESR HepG2	EN1_2	A	3.461529306	ENCODE
93	rs2796441	G	A	G	0.206639	0.151748	EDR islet	EDR islet				
							ESR HepG2	ESR HepG2	POU4F2	A	3.068115281	JASPAR
									POU4F2_2	A	2.886756051	ENCODE
									LHX9_3	A	2.670806028	ENCODE
									HESX1_2	A	2.504857436	ENCODE
									BCL6B	A	2.199400857	JASPAR
									BCL6B_2	A	2.03441786	ENCODE
94	rs73554552	C	A	A	0.027729	0.028216	ESR islet	EDR K562				
								ESR islet	FOXA_known2	C	2.379450673	ENCODE
									ZNF317	A	2.371408315	JASPAR

Table S3. Disruption of transcription factor motifs by prioritized SNPs. Difference in the binding predictions of transcription factors with motifs that overlap prioritized SNPs. Difference measured by the absolute value of the difference in the log of *P*-values for binding at each allele (log difference column). The "high score allele" column refers to the allele with the greatest enhancer probability score from TREDNet.

Layer no	Type	Activation	Parameters
1	Convolution	ReLU	filters=320, kernel size=8
2	Convolution	ReLU	filters=320, kernel size=8
3	Dropout		probability=0.2
4	Max Pooling		pool size=4, stride=4
5	Convolution	ReLU	filters=480, kernel size=8
6	Convolution	ReLU	filters=480, kernel size=8
7	Dropout		probability=0.2
8	Max Pooling		pool size=4, stride=4
9	Convolution	ReLU	filters=640, kernel size=8
1	Convolution	ReLU	filters=640, kernel size=8
11	Dropout		probability=0.2
12	Dense		units=100
13	Dense		units=50
14	Sigmoid		

Table S4. TREDNet phase one network architecture. All kernels were subject to *maximum normalization* value of 0.9. Total trained parameters: $\sim$143 million. Model was trained with *Adadelta* optimizer using *binary cross entropy* as a cost function.

Layer no	Type	Activation	Parameters
1	Convolution	ReLU	filters=64, kernel size=4
2	Batch Normalization		
3	Max Pooling		pool size=2, stride=2
4	Dropout		probability=0.4
5	Convolution	ReLU	filters=128, kernel size=2
6	Dropout		probability=0.4
7	Dense		units=100
8	Dense		Units=50
9	Dense		units=1
10	Sigmoid		

Table S5. TREDNet phase two network architecture. All kernels were subject to *maximum normalization* value of 0.9. Total trained parameters: $\sim$12 million. Model was trained with *RMSProp* optimizer using *binary cross entropy* as a cost function.

Layer no	Type	Activation	Parameters
1	Convolution	ReLU	filters=256, kernel size=1
2	Batch Normalization		
3	Dropout		probability=0.2
4	Convolution	ReLU	filters=256, kernel size=1
5	Batch Normalization		
6	Dropout		probability=0.5
7	Dense	ReLU	units=100
8	Dense		units=1
9	Sigmoid		

Table S6. EDR/ESR detection network architecture. All kernels were subject to *maximum normalization* value of 1.0. Model was trained with *ADAM* optimizer using *binary cross entropy* as a cost function.

Primer Name	Sequence	rsID	Allele	SNP info	Genomic region	Padding (5' and 3')	Strand	Repeat Masking
rs75638565_C_For	GAGGCTTTTCCGTTCTGGCCT	rs75638565	C	hg19_dbSnp153	chr11:14430386-14430406	10	+	none
rs75638565_C_Rev	AGGCCAGAACGGAAAAGCCTC							
rs75638565_T_For	GAGGCTTTTCTGTTCTGGCCT		T					
rs75638565_T_Rev	AGGCCAGAACAGAAAAGCCTC							
rs75336838_C_For	AATTACATTTCCCACTTTATG	rs75336838	C	hg19_dbSnp153	chr11:14576781-14576801	10	+	none
rs75336838_C_Rev	CATAAAGTGGGAAATGTAATT							
rs75336838_T_For	AATTACATTTTCCACTTTATG		T					
rs75336838_T_Rev	CATAAAGTGGAAAATGTAATT							
rs117720468_G_For	TCCAAGTTTTGGATTCTCTGA	rs117720468	G	hg19_dbSnp153	chr11:14731656-14731676	10	+	none
rs117720468_G_Rev	TCAGAGAATCCAAAACTTGGA							
rs117720468_C_For	TCCAAGTTTTCGATTCTCTGA		C					
rs117720468_C_Rev	TCAGAGAATCGAAAACTTGGA							

Table S7. Probes used for EMSA experiments. Each forward and reverse oligo for the biotinylated probes were 5' biotinylated.

Fragment name	Sequence
rs75638565 C/C	AAAGCAGGCAGAAAAACGTGAAAAGGCGAGACTGGCCTAGCCTCCCAGACTACATATTTCTTCCATGCTGGATTCTTCCTGCCCTTGAACATCAGACTCCAAGATCTTCAGTTTT<br>GAGACTCGGACTGGCTCTCCTTGCTCCTCAAGCTTGCAGACAGCCTATTGTGGGAACTTGTGATTGTGTAGATTAATACTTAATAAATTCATATATATATATATATATCCTACTA<br>GTTCTGTCCCTTTAGAGAACCCTAATACACAGATGAAGACAATGGCATTGAGGATGACAAAACAGTAAGATGGAAGGAACTTGGGATCCTTAATGACTGTGTGGGTCAGAGAGGC<br>TTTTC**<u>C</u>**GTTCTGGCCTGCTTACCTTGGGGTGCTTTTGTTAGAGAGAAATAAACTTGCATTTTCTTAAGTCATTGTATTCTGGAACCTTTTTAAAGGTGCTAAAAGCAACTTAGCC<br>TTTACCCTAACATCCATTCAATATTTTTTTGGAAATAGGCTATCTCTTAGAGCACTACCCTCAGTGTTTGAGCAAAATAATTTACATATGCATATATTTTCTGTCTATTTAAACA<br>GGAATTTAAGGGGGCTAAGCACTGCTCTGCCACAGAACAGGCAAGTCATTCCTTCCTTTCACAATATGTGTTGAAGCCAGGCGCAGTGGCTCACGCCTGTAATCCCAGCACTTTG<br>GGAGGCTGAGG
rs75638565 T/T	AAAGCAGGCAGAAAAACGTGAAAAGGCGAGACTGGCCTAGCCTCCCAGACTACATATTTCTTCCATGCTGGATTCTTCCTGCCCTTGAACATCAGACTCCAAGATCTTCAGTTTT<br>GAGACTCGGACTGGCTCTCCTTGCTCCTCAAGCTTGCAGACAGCCTATTGTGGGAACTTGTGATTGTGTAGATTAATACTTAATAAATTCATATATATATATATATATCCTACTA<br>GTTCTGTCCCTTTAGAGAACCCTAATACACAGATGAAGACAATGGCATTGAGGATGACAAAACAGTAAGATGGAAGGAACTTGGGATCCTTAATGACTGTGTGGGTCAGAGAGGC<br>TTTTC**<u>T</u>**GTTCTGGCCTGCTTACCTTGGGGTGCTTTTGTTAGAGAGAAATAAACTTGCATTTTCTTAAGTCATTGTATTCTGGAACCTTTTTAAAGGTGCTAAAAGCAACTTAGCC<br>TTTACCCTAACATCCATTCAATATTTTTTTGGAAATAGGCTATCTCTTAGAGCACTACCCTCAGTGTTTGAGCAAAATAATTTACATATGCATATATTTTCTGTCTATTTAAACA<br>GGAATTTAAGGGGGCTAAGCACTGCTCTGCCACAGAACAGGCAAGTCATTCCTTCCTTTCACAATATGTGTTGAAGCCAGGCGCAGTGGCTCACGCCTGTAATCCCAGCACTTTG<br>GGAGGCTGAGG
rs75336838 C/C	ACAGATAGATTTGGAACCCAGGCCAACACTCTTACTACTATGCTTTATTGCCTCTCTAAACAGATTTTTATTTCTTCATAGAGTTGCTTTTACTGAGCACTGCATTGGCATTTTG<br>CCATTCACCTATGTTGAGAGACATGACAAGAAACAGCTCTATGAAACTTATTGTTGCATTTACTGTTGGCAAGTTAAGGAGGATGTGGTTCACTACGTCACATGTGTCCTCTTTA<br>GGAGAACAGATGGGAGAACTGTCTCTCAGATCGCAGTACCAGGAATAGCTATATTTCTCCTGAGTAAGTGGTTTGTTTTTATCTCTTGGACATTGTGAAACCATGTAAACAATTA<br>CATTT**<u>C</u>**CCACTTTATGCTTGCTGCTAAAAAGCTCAGAACCAAATTTTCAGCCTATCTTTAGCAATGATGTGTACTGGATGATGTGCATTACTTCTGACTTCATCTTATTTTTTTC<br>TACCCTAATTTTCCCTTCTCTCTTATTTCCTCTTTAAAATTTTTTTTGCTTTAGTCTTTTAAAAAAATAGAAACATACAAGAATTCTTCAGGCATCATATCTAGAGGAATAAAAAT<br>AAAAGAAAAAAATATATAAGCACATAGCATAAAAAGTCAAATCCAAGGCTTTTAACAACAAAAAGAAACAGTATTTTGCTCACTCTTCCCCACCTCTGTGTTTTGCTCCACACAGC<br>TAACTGTAGTC
rs75336838 T/T	ACAGATAGATTTGGAACCCAGGCCAACACTCTTACTACTATGCTTTATTGCCTCTCTAAACAGATTTTTATTTCTTCATAGAGTTGCTTTTACTGAGCACTGCATTGGCATTTTG<br>CCATTCACCTATGTTGAGAGACATGACAAGAAACAGCTCTATGAAACTTATTGTTGCATTTACTGTTGGCAAGTTAAGGAGGATGTGGTTCACTACGTCACATGTGTCCTCTTTA<br>GGAGAACAGATGGGAGAACTGTCTCTCAGATCGCAGTACCAGGAATAGCTATATTTCTCCTGAGTAAGTGGTTTGTTTTTATCTCTTGGACATTGTGAAACCATGTAAACAATTA<br>CATTT**<u>T</u>**CCACTTTATGCTTGCTGCTAAAAAGCTCAGAACCAAATTTTCAGCCTATCTTTAGCAATGATGTGTACTGGATGATGTGCATTACTTCTGACTTCATCTTATTTTTTTC<br>TACCCTAATTTTCCCTTCTCTCTTATTTCCTCTTTAAAATTTTTTTTGCTTTAGTCTTTTAAAAAAATAGAAACATACAAGAATTCTTCAGGCATCATATCTAGAGGAATAAAAAT<br>AAAAGAAAAAAATATATAAGCACATAGCATAAAAAGTCAAATCCAAGGCTTTTAACAACAAAAAGAAACAGTATTTTGCTCACTCTTCCCCACCTCTGTGTTTTGCTCCACACAGC<br>TAACTGTAGTC
rs117720468 G/G	CTTATGAGGGTTCCTGTTAGACTATTGGATATTTTTTGTTGTTTTCAGTTATATATGAAAATAATGGTGATAAAAAATACTTTTTTTCCTGCTTAACTTTGGTGTCCTTGCTTTTT<br>GGATATATGTTCTTAATTGTATTATTCCTATTCATGGTTTCACAGATATCTTTAATCTCACCCTATCCAAAGGAATTCTAAAGGTAAAATAACACCCTACATTGATAGCTTGGTC<br>CATTAAGTCATTTGTCTTGGGGAAAAGTTGTAGCATAAAGTCATAGTCAGTTTTTGGTTAGGAGGACCTTATAGAAGAGATGAATGAGCATACATACACCCTGGAGTTAGTCCAA<br>GTTTT**<u>G</u>**GATTCTCTGAAGCTTACCTCTGTAACTTAATCTTTCTGAACTTTAGTTTCCTCATCTAGAGAATGGTTTGTGATATTACTGCTCTGTTAATGTTATTGTGCTCCTTCAA<br>TGAGATAGTGTATATAAAGGTCTAGTGTGGGCTCTGGCAGAGATGTGGTGCTGAATAGATGATTTATTTTTTTGATTGATTGTGATTGCCATTATTTTTGGTTTCCTAGGACATC<br>CTCTTCCACCCAGTCCCTCTTCTTATGGAAATATATCTTGTCCAACCATCCCCTCCCACCAACCACGAGAGAGGTGGGCACATTATTCATCCTGGCCAATTTAGATTAATCCTCT<br>AAATATAATGG

rs117720468 C/C	CTTATGAGGGTTCCTGTTAGACTATTGGATATTTTTTGTTGTTTTCAGTTATATATGAAAATAATGGTGATAAAAATACTTTTTTTCCTGCTTAACTTTGGTGTCCTTGCTTTTT<br>GGATATATGTTCTTAATTGTATTATTCCTATTCATGGTTTCACAGATATCTTTAATCTCACCCTATCCAAAGGAATTCTAAAGGTAAAATAACACCCTACATTGATAGCTTGGTC<br>CATTAAGTCATTTGTCTTGGGGAAAAGTTGTAGCATAAAGTCATAGTCAGTTTTTGGTTAGGAGGACCTTATAGAAGAGATGAATGAGCATACATACACCCTGGAGTTAGTCCAA<br>GTTTT**C**GATTCTCTGAAGCTTACCTCTGTAACTTAATCTTTCTGAACTTTAGTTTCCTCATCTAGAGAATGGTTTGTGATATTACTGCTCTGTTAATGTTATTGTGCTCCTTCAA<br>TGAGATAGTGTATATAAAGGTCTAGTGTGGGCTCTGGCAGAGATGTGGTGCTGAATAGATGATTTATTTTTTTGATTGATTGTGATTGCCATTATTTTTGGTTTCCTAGGACATC<br>CTCTTCCACCCAGTCCCTCTTCTTATGGAAATATATCTTGTCCAACCATCCCCTCCCACCAACCACGAGAGAGGTGGGCACATTATTCATCCTGGCCAATTTAGATTAATCCTCT<br>AAATATAATGG

Table S8. Luciferase reporter assay oligonucleotides. 701bp DNA fragments used for luciferase assay.
