# Supplementary material for: Modeling islet enhancers using deep learning identifies candidate causal variants at loci associated with T2D and glycemic traits
Source: Proc Natl Acad Sci U S A. 2023 Aug 21;120(35):e2206612120. doi: 10.1073/pnas.2206612120 (PMC10469333; doi:10.1073/pnas.2206612120)
Supplement: Supplementary file 2 — Dataset S01 (DOCX) [file pnas.2206612120.sd01.docx]

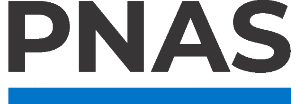


**DIAMANTE Consortium Authors**

The following authors were part of the Diabetes Meta-Analysis of Trans-Ethnic association studies (DIAMANTE) Consortium:

Anubha Mahajan^1,2,278^, Cassandra N. Spracklen^3,4^, Weihua Zhang^5,6^, Maggie C. Y. Ng^7,8,9^, Lauren E Petty^7^, Hidetoshi Kitajima^2,10,11,12^, Grace Z. Yu^1,2^, Sina Rüeger^13^, Leo Speidel^14,15^, Young Jin Kim^16^, Momoko Horikoshi^17^, Josep M. Mercader^18,19,20^, Daniel Taliun^21^, Sanghoon Moon^16^, Soo-Heon Kwak^22^, Neil R. Robertson^1,2^, Nigel W. Rayner^1,2,23,24^, Marie Loh^5,25,26^, Bong-Jo Kim^16^, Joshua Chiou^27,279^, Irene Miguel-Escalada^28,29^, Pietro della Briotta Parolo^13^, Kuang Lin^30^, Fiona Bragg^30,31^, Michael H. Preuss^32^, Fumihiko Takeuchi^33^, Jana Nano^34^, Xiuqing Guo^35^, Amel Lamri^36,37^, Masahiro Nakatochi^38^, Robert A. Scott^39^, Jung-Jin Lee^40^, Alicia Huerta-Chagoya^41,280^, Mariaelisa Graff^42^, Jin-Fang Chai^43^, Esteban J Parra^44^, Jie Yao^35^, Lawrence F. Bielak^45^, Yasuharu Tabara^46^, Yang Hai^35^, Valgerdur Steinthorsdottir^47^, James P. Cook^48^, Mart Kals^49^, Niels Grarup^50^, Ellen M. Schmidt^21^, Ian Pan^51^, Tamar Sofer^52,53,54^, Matthias Wuttke^55^, Chloe Sarnowski^56,281^, Christian Gieger^57,58,59^, Darryl Nousome^60^, Stella Trompet^61,62^, Jirong Long^63^, Meng Sun^2^, Lin Tong^64^, Wei-Min Chen^65^, Meraj Ahmad^66^, Raymond Noordam^62^, Victor J. Y. Lim^43^, Claudia H. T. Tam^67,68^, Yoonjung Yoonie Joo^69,70,282^, Chien-Hsiun Chen^71^, Laura M. Raffield^3^, Cécile Lecoeur^72,73^, Bram Peter Prins^23^, Aude Nicolas^74^, Lisa R. Yanek^75^, Guanjie Chen^76^, Richard A. Jensen^77^, Salman Tajuddin^78^, Edmond K. Kabagambe^63,283^, Ping An^79^, Anny H. Xiang^80^, Hyeok Sun Choi^81^, Brian E. Cade^20,53^, Jingyi Tan^35^, Jack Flanagan^17,48^, Fernando Abaitua^2,284^, Linda S. Adair^82^, Adebowale Adeyemo^76^, Carlos A. Aguilar-Salinas^83^, Masato Akiyama^84,85^, Sonia S. Anand^36,37,86^, Alain Bertoni^87^, Zheng Bian^88^, Jette Bork-Jensen^50^, Ivan Brandslund^89,90^, Jennifer A. Brody^77^, Chad M. Brummett^91^, Thomas A. Buchanan^92^, Mickaël Canouil^72,73^, Juliana C. N. Chan^67,68,93,94^, Li-Ching Chang^71^, Miao-Li Chee^95^, Ji Chen^96,285^, Shyh-Huei Chen^97^, Yuan-Tsong Chen^71^, Zhengming Chen^30,31^, Lee-Ming Chuang^98,99^, Mary Cushman^100^, Swapan K. Das^101^, H. Janaka de Silva^102^, George Dedoussis^103^, Latchezar Dimitrov^8^, Ayo P. Doumatey^76^, Shufa Du^82,104^, Qing Duan^3^, Kai-Uwe Eckardt^105,106^, Leslie S. Emery^107^, Daniel S. Evans^108^, Michele K. Evans^78^, Krista Fischer^49^, James S. Floyd^77^, Ian Ford^109^, Myriam Fornage^110^, Oscar H. Franco^34^, Timothy M. Frayling^111^, Barry I. Freedman^112^, Christian Fuchsberger^21,113^, Pauline Genter^114^, Hertzel C. Gerstein^36,37,86^, Vilmantas Giedraitis^115^, Clicerio González-Villalpando^116^, Maria Elena González-Villalpando^116^, Mark O. Goodarzi^117^, Penny Gordon-Larsen^82,104^, David Gorkin^118^, Myron Gross^119^, Yu Guo^88^, Sophie Hackinger^23^, Sohee Han^16^, Andrew T. Hattersley^120^, Christian Herder^57,121,122^, Annie-Green Howard^104,123^, Willa Hsueh^124^, Mengna Huang^51,125^, Wei Huang^126^, Yi-Jen Hung^127,128^, Mi Yeong Hwang^16^, Chii-Min Hwu^129,130^, Sahoko Ichihara^131^, Mohammad Arfan Ikram^34^, Martin Ingelsson^115^, Md Tariqul Islam^132^, Masato Isono^33^, Hye-Mi Jang^16^, Farzana Jasmine^64^, Guozhi Jiang^67,68^, Jost B. Jonas^133^, Marit E. Jørgensen^134,135^, Torben Jørgensen^136,137,138^, Yoichiro Kamatani^84,139^, Fouad R. Kandeel^140^, Anuradhani Kasturiratne^141^, Tomohiro Katsuya^142,143^, Varinderpal Kaur^19^, Takahisa Kawaguchi^46^, Jacob M. Keaton^8,63,286^, Abel N. Kho^144,145^, Chiea-Chuen Khor^146^, Muhammad G. Kibriya^64^, Duk-Hwan Kim^147^, Katsuhiko Kohara^148,287^, Jennifer Kriebel^57,58,59^, Florian Kronenberg^149^, Johanna Kuusisto^150^, Kristi Läll^49,151^, Leslie A. Lange^152^, Myung-Shik Lee^153,154^, Nanette R. Lee^155^, Aaron Leong^19,156,157^, Liming Li^158^, Yun Li^3^, Ruifang Li-Gao^159^, Symen Ligthart^34^, Cecilia M. Lindgren^2,160,161^, Allan Linneberg^136,162^, Ching-Ti Liu^56^, Jianjun Liu^146,163^, Adam E. Locke^164,165,288^, Tin Louie^107^, Jian'an Luan^39^, Andrea O. Luk^67,68^, Xi Luo^166^, Jun Lv^158^, Valeriya Lyssenko^167,168^, Vasiliki Mamakou^169^, K. Radha Mani^66,277^, Thomas Meitinger^170,171,172^, Andres Metspalu^49^, Andrew D. Morris^173^, Girish N. Nadkarni^32,174,175^, Jerry L. Nadler^176^, Michael A. Nalls^74,177,178^, Uma Nayak^65^, Suraj S. Nongmaithem^66^, Ioanna Ntalla^179^, Yukinori Okada^180,181,182^, Lorena Orozco^183^, Sanjay R. Patel^184^, Mark A. Pereira^185^, Annette Peters^57,58,172^, Fraser J. Pirie^186^, Bianca Porneala^157^, Gauri Prasad^187,188^, Sebastian Preissl^118^, Laura J. Rasmussen-Torvik^189^, Alexander P. Reiner^190^, Michael Roden^57,121,122^, Rebecca Rohde^42^, Kathryn Roll^35^, Charumathi Sabanayagam^95,191,192^, Maike Sander^193,194,195^, Kevin Sandow^35^, Naveed Sattar^196^, Sebastian Schönherr^149^, Claudia Schurmann^32,174,197^, Mohammad Shahriar^64,289^, Jinxiu Shi^126^, Dong Mun Shin^16^, Daniel Shriner^76^, Jennifer A. Smith^45,198^, Wing Yee So^67,93^, Alena Stančáková^150^, Adrienne M. Stilp^107^, Konstantin Strauch^199,200,201^, Ken Suzuki^17,84,180,202^, Atsushi Takahashi^84,203^, Kent D. Taylor^35^, Barbara Thorand^57,58^, Gudmar Thorleifsson^47^, Unnur Thorsteinsdottir^47,204^, Brian Tomlinson^67,205^, Jason M. Torres^2,290^, Fuu-Jen Tsai^206^, Jaakko Tuomilehto^207,208,209,210^, Teresa Tusie-Luna^211,212^, Miriam S. Udler^18,19,156^, Adan Valladares-Salgado^213^, Rob M. van Dam^43,163^, Jan B. van Klinken^214,215,216^, Rohit Varma^217^, Marijana Vujkovic^218^, Niels Wacher-Rodarte^219^, Eleanor Wheeler^39^, Eric A. Whitsel^42,220^, Ananda R. Wickremasinghe^141^, Ko Willems van Dijk^214,215,221^, Daniel R. Witte^222,223^, Chittaranjan S. Yajnik^224^, Ken Yamamoto^225^, Toshimasa Yamauchi^202^, Loïc Yengo^226^, Kyungheon Yoon^16^, Canqing Yu^158^, Jian-Min Yuan^227,228^, Salim Yusuf^36,37,86^, Liang Zhang^95^, Wei Zheng^63^, FinnGen, eMERGE Consortium, Leslie J. Raffel^229^, Michiya Igase^230^, Eli Ipp^114^, Susan Redline^20,53,231^, Yoon Shin Cho^81^, Lars Lind^232^, Michael A. Province^79^, Craig L. Hanis^233^, Patricia A. Peyser^45^, Erik Ingelsson^234,235^, Alan B. Zonderman^78^, Bruce M. Psaty^77,236,237^, Ya-Xing Wang^238^, Charles N. Rotimi^76^, Diane M. Becker^75^, Fumihiko Matsuda^46^, Yongmei Liu^87,239^, Eleftheria Zeggini^23,24,240^, Mitsuhiro Yokota^241^, Stephen S. Rich^242^, Charles Kooperberg^190^, James S. Pankow^185^, James C. Engert^243,244^, Yii-Der Ida Chen^35^, Philippe Froguel^72,73,245^, James G. Wilson^246^, Wayne H. H. Sheu^128,130,247^, Sharon L. R. Kardia^45^, Jer-Yuarn Wu^71^, M. Geoffrey Hayes^69,248,249^, Ronald C. W. Ma^67,68,93,94^, Tien-Yin Wong^95,191,192^, Leif Groop^13,167^, Dennis O. Mook-Kanamori^159^, Giriraj R. Chandak^66^, Francis S. Collins^250^, Dwaipayan Bharadwaj^187,251^, Guillaume Paré^37,252^, Michèle M. Sale^65,277^, Habibul Ahsan^64^, Ayesha A. Motala^186^, Xiao-Ou Shu^63^, Kyong-Soo Park^22,253,254^, J. Wouter Jukema^61,255^, Miguel Cruz^213^, Roberta McKean-Cowdin^60^, Harald Grallert^57,58,59^, Ching-Yu Cheng^95,191,192^, Erwin P. Bottinger^32,174,197^, Abbas Dehghan^5,34,256^, E-Shyong Tai^43,163,257^, Josée Dupuis^56^, Norihiro Kato^33^, Markku Laakso^150^, Anna Köttgen^55^, Woon-Puay Koh^258,259^, Colin N. A. Palmer^260^, Simin Liu^51,125,261^, Goncalo Abecasis^21^, Jaspal S. Kooner^6,256,262,263^, Ruth J. F. Loos^32,50,264^, Kari E. North^42^, Christopher A. Haiman^60^, Jose C. Florez^18,19,156^, Danish Saleheen^40,265,266^, Torben Hansen^50^, Oluf Pedersen^50^, Reedik Mägi^49^, Claudia Langenberg^39,267^, Nicholas J. Wareham^39^, Shiro Maeda^17,268,269^, Takashi Kadowaki^202,291^, Juyoung Lee^16^, Iona Y. Millwood^30,31^, Robin G. Walters^30,31^, Kari Stefansson^47,204^, Simon R. Myers^2,270^, Jorge Ferrer^28,29,271^, Kyle J. Gaulton^193,194^, James B. Meigs^18,156,157^, Karen L. Mohlke^3^, Anna L. Gloyn^1,2,272,273^, Donald W. Bowden^8,9,274^, Jennifer E. Below^7^, John C. Chambers^5,6,25,256,262^, Xueling Sim^43^, Michael Boehnke^21^, Jerome I. Rotter^35^, Mark I. McCarthy^1,2,272,278^, and Andrew P. Morris^2,48,49,275,276^

^1^Oxford Centre for Diabetes, Endocrinology and Metabolism, Radcliffe Department of Medicine, University of Oxford, Oxford, UK. ^2^Wellcome Centre for Human Genetics, Nuffield Department of Medicine, University of Oxford, Oxford, UK. ^3^Department of Genetics, University of North Carolina at Chapel Hill, Chapel Hill, NC, USA. ^4^Department of Epidemiology and Biostatistics, University of Massachusetts-Amherst, Amherst, MA, USA. ^5^Department of Epidemiology and Biostatistics, Imperial College London, London, UK. ^6^Department of Cardiology, Ealing Hospital, London North West Healthcare NHS Trust, London, UK. ^7^Vanderbilt Genetics Institute, Division of Genetic Medicine, Vanderbilt University Medical Center, Nashville, TN, USA. ^8^Center for Genomics and Personalized Medicine Research, Wake Forest School of Medicine, Winston-Salem, NC, USA. ^9^Department of Biochemistry, Wake Forest School of Medicine, Winston-Salem, NC, USA. ^10^The Advanced Research Center for Innovations in Next-Generation Medicine (INGEM), Tohoku University, Sendai, Japan. ^11^Department of Integrative Genomics, Tohoku Medical Megabank Organization, Tohoku University, Sendai, Japan. ^12^Cancer Center, Tohoku University Hospital, Tohoku University, Sendai, Japan. ^13^Institute for Molecular Medicine Finland (FIMM), University of Helsinki, Helsinki, Finland. ^14^Genetics Institute, University College London, London, UK. ^15^Francis Crick Institute, London, UK. ^16^Division of Genome Science, Department of Precision Medicine, National Institute of Health, Cheongju-si, Republic of Korea. ^17^Laboratory for Genomics of Diabetes and Metabolism, RIKEN Center for Integrative Medical Sciences, Yokohama, Japan. ^18^Programs in Metabolism and Medical & Population Genetics, Broad Institute of Harvard and MIT, Cambridge, MA, USA. ^19^Diabetes Unit and Center for Genomic Medicine, Massachusetts General Hospital, Boston, MA, USA. ^20^Harvard Medical School, Boston, MA, USA. ^21^Department of Biostatistics and Center for Statistical Genetics, University of Michigan, Ann Arbor, MI, USA. ^22^Department of Internal Medicine, Seoul National University Hospital, Seoul, South Korea. ^23^Department of Human Genetics, Wellcome Sanger Institute, Hinxton, UK. ^24^Institute of Translational Genomics, Helmholtz Zentrum München, German Research Center for Environmental Health, Neuherberg, Germany. ^25^Lee Kong Chian School of Medicine, Nanyang Technological University, Singapore, Singapore. ^26^Translational Laboratory in Genetic Medicine (TLGM), Agency for Science, Technology and Research (A*STAR) and National University of Singapore (NUS), Singapore, Singapore. ^27^Biomedical Sciences Graduate Studies Program, University of California San Diego, La Jolla, CA, USA. ^28^Regulatory Genomics and Diabetes, Centre for Genomic Regulation, The Barcelona Institute of Science and Technology, Barcelona, Spain. ^29^Centro de Investigación Biomédica en Red Diabetes y Enfermedades Metabólicas asociadas (CIBERDEM), Madrid, Spain. ^30^Nuffield Department of Population Health, University of Oxford, Oxford, UK. ^31^Medical Research Council Population Health Research Unit, University of Oxford, Oxford, UK. ^32^The Charles Bronfman Institute for Personalized Medicine, Icahn School of Medicine at Mount Sinai, New York, NY, USA. ^33^Department of Gene Diagnostics and Therapeutics, Research Institute, National Center for Global Health and Medicine, Tokyo, Japan. ^34^Department of Epidemiology, Erasmus University Medical Center, Rotterdam, The Netherlands. ^35^The Institute for Translational Genomics and Population Sciences, Department of Pediatrics, The Lundquist Institute for Biomedical Innovation (formerly Los Angeles Biomedical Research Institute) at Harbor-UCLA Medical Center, Torrance, CA, USA. ^36^Department of Medicine, McMaster University, Hamilton, ON, Canada. ^37^Population Health Research Institute, Hamilton Health Sciences and McMaster University, Hamilton, ON, Canada. ^38^Public Health Informatics Unit, Department of Integrated Health Sciences, Nagoya University Graduate School of Medicine, Nagoya, Japan. ^39^MRC Epidemiology Unit, Institute of Metabolic Science, University of Cambridge, Cambridge, UK. ^40^Division of Translational Medicine and Human Genetics, University of Pennsylvania, Philadelphia, PA, USA. ^41^Consejo Nacional de Ciencia y Tecnología (CONACYT), Instituto Nacional de Ciencias Médicas y Nutrición Salvador Zubirán, Mexico City, Mexico. ^42^Department of Epidemiology, Gillings School of Global Public Health, University of North Carolina at Chapel Hill, Chapel Hill, NC, USA. ^43^Saw Swee Hock School of Public Health, National University of Singapore and National University Health System, Singapore, Singapore. ^44^Department of Anthropology, University of Toronto at Mississauga, Mississauga, ON, Canada. ^45^Department of Epidemiology, School of Public Health, University of Michigan, Ann Arbor, MI, USA. ^46^Center for Genomic Medicine, Kyoto University Graduate School of Medicine, Kyoto, Japan. ^47^deCODE Genetics, Amgen inc., Reykjavik, Iceland. ^48^Department of Health Data Science, University of Liverpool, Liverpool, UK. ^49^Estonian Genome Centre, Institute of Genomics, University of Tartu, Tartu, Estonia. ^50^Novo Nordisk Foundation Center for Basic Metabolic Research, Faculty of Health and Medical Sciences, University of Copenhagen, Copenhagen, Denmark. ^51^Department of Epidemiology, Brown University School of Public Health, Providence, RI, USA. ^52^Department of Biostatistics, Harvard University, Boston, MA, USA. ^53^Division of Sleep and Circadian Disorders, Brigham and Women's Hospital, Boston, MA, USA. ^54^Department of Medicine, Harvard University, Boston, MA, USA. ^55^Institute of Genetic Epidemiology, Department of Data Driven Medicine, Faculty of Medicine and Medical Center, University of Freiburg, Freiburg, Germany. ^56^Department of Biostatistics, Boston University School of Public Health, Boston, MA, USA. ^57^German Center for Diabetes Research (DZD), Neuherberg, Germany. ^58^Institute of Epidemiology, Helmholtz Zentrum München, German Research Center for Environmental Health, Neuherberg, Germany. ^59^Research Unit of Molecular Epidemiology, Helmholtz Zentrum München, German Research Center for Environmental Health, Neuherberg, Germany. ^60^Department of Population and Public Health Sciences, Keck School of Medicine of USC, Los Angeles, CA, USA. ^61^Department of Cardiology, Leiden University Medical Center, Leiden, The Netherlands. ^62^Section of Gerontology and Geriatrics, Department of Internal Medicine, Leiden University Medical Center, Leiden, The Netherlands. ^63^Division of Epidemiology, Department of Medicine, Institute for Medicine and Public Health, Vanderbilt Genetics Institute, Vanderbilt University Medical Center, Nashville, TN, USA. ^64^Institute for Population and Precision Health, The University of Chicago, Chicago, IL, USA. ^65^Department of Public Health Sciences and Center for Public Health Genomics, University of Virginia School of Medicine, Charlottesville, VA, USA. ^66^Genomic Research on Complex Diseases (GRC-Group), CSIR-Centre for Cellular and Molecular Biology (CSIR-CCMB), Hyderabad, India. ^67^Department of Medicine and Therapeutics, The Chinese University of Hong Kong, Hong Kong, China. ^68^Chinese University of Hong Kong-Shanghai Jiao Tong University Joint Research Centre in Diabetes Genomics and Precision Medicine, The Chinese University of Hong Kong, Hong Kong, China. ^69^Division of Endocrinology, Metabolism, and Molecular Medicine, Department of Medicine, Northwestern University Feinberg School of Medicine, Chicago, IL, USA. ^70^Department of Health and Biomedical Informatics, Northwestern University Feinberg School of Medicine, Chicago, IL, USA. ^71^Institute of Biomedical Sciences, Academia Sinica, Taipei, Taiwan. ^72^Inserm U1283, CNRS UMR 8199, European Genomic Institute for Diabetes, Institut Pasteur de Lille, Lille, France. ^73^University of Lille, Lille University Hospital, Lille, France. ^74^Laboratory of Neurogenetics, National Institute on Aging, National Institutes of Health, Bethesda, MD, USA. ^75^Department of Medicine, Johns Hopkins University School of Medicine, Baltimore, MD, USA. ^76^Center for Research on Genomics and Global Health, National Human Genome Research Institute, National Institutes of Health, Bethesda, MD, USA. ^77^Cardiovascular Health Research Unit, Department of Medicine, University of Washington, Seattle, WA, USA. ^78^Laboratory of Epidemiology and Population Sciences, National Institute on Aging, National Institutes of Health, Baltimore, MD, USA. ^79^Division of Statistical Genomics, Washington University School of Medicine, St. Louis, MO, USA. ^80^Department of Research and Evaluation, Division of Biostatistics Research, Kaiser Permanente of Southern California, Pasadena, CA, USA. ^81^Department of Biomedical Science, Hallym University, Chuncheon, South Korea. ^82^Department of Nutrition, Gillings School of Global Public Health, University of North Carolina at Chapel Hill, Chapel Hill, NC, USA. ^83^Unidad de Investigación en Enfermedades Metabólicas and Departamento de Endocrinología y Metabolismo, Instituto Nacional de Ciencias Médicas y Nutrición Salvador Zubirán, Mexico City, Mexico. ^84^Laboratory for Statistical and Translational Genetics, RIKEN Center for Integrative Medical Sciences, Yokohama, Japan. ^85^Department of Ocular Pathology and Imaging Science, Graduate School of Medical Sciences, Kyushu University, Fukuoka, Japan. ^86^Department of Health Research Methods, Evidence, and Impact, McMaster University, Hamilton, ON, Canada. ^87^Department of Epidemiology and Prevention, Division of Public Health Sciences, Wake Forest School of Medicine, Winston-Salem, NC, USA. ^88^Chinese Academy of Medical Sciences, Beijing, China. ^89^Institute of Regional Health Research, University of Southern Denmark, Odense, Denmark. ^90^Department of Clinical Biochemistry, Vejle Hospital, Vejle, Denmark. ^91^Department of Anesthesiology, University of Michigan Medical School, Ann Arbor, MI, USA. ^92^Department of Medicine, Division of Endocrinology and Diabetes, Keck School of Medicine of USC, Los Angeles, CA, USA. ^93^Hong Kong Institute of Diabetes and Obesity, The Chinese University of Hong Kong, Hong Kong, China. ^94^Li Ka Shing Institute of Health Sciences, The Chinese University of Hong Kong, Hong Kong, China. ^95^Singapore Eye Research Institute, Singapore National Eye Centre, Singapore, Singapore. ^96^Wellcome Sanger Institute, Hinxton, UK. ^97^Department of Biostatistics and Data Science, Wake Forest School of Medicine, Winston-Salem, NC, USA. ^98^Division of Endocrinology and Metabolism, Department of Internal Medicine, National Taiwan University Hospital, Taipei, Taiwan. ^99^Institute of Epidemiology and Preventive Medicine, National Taiwan University, Taipei, Taiwan. ^100^Department of Medicine, University of Vermont, Colchester, VT, USA. ^101^Section on Endocrinology and Metabolism, Department of Internal Medicine, Wake Forest School of Medicine, Winston-Salem, NC, USA. ^102^Department of Medicine, Faculty of Medicine, University of Kelaniya, Ragama, Sri Lanka. ^103^Department of Nutrition and Dietetics, Harokopio University of Athens, Athens, Greece. ^104^Carolina Population Center, University of North Carolina at Chapel Hill, Chapel Hill, NC, USA. ^105^Department of Nephrology and Medical Intensive Care Medicine, Charité Universitätsmedizin Berlin, Berlin, Germany. ^106^Department of Nephrology and Hypertension, Friedrich-Alexander-Universität Erlangen-Nürnberg, Erlangen, Germany. ^107^Department of Biostatistics, University of Washington, Seattle, WA, USA. ^108^California Pacific Medical Center Research Institute, San Francisco, CA, USA. ^109^Robertson Centre for Biostatistics, University of Glasgow, Glasgow, UK. ^110^Institute of Molecular Medicine, University of Texas Health Science Center at Houston, Houston, TX, USA. ^111^Genetics of Complex Traits, University of Exeter Medical School, University of Exeter, Exeter, UK. ^112^Department of Internal Medicine, Wake Forest School of Medicine, Winston-Salem, NC, USA. ^113^Institute for Biomedicine, Eurac Research, Affiliated Institute of the University of Lübeck, Bolzano, Italy. ^114^Department of Medicine, Division of Endocrinology and Metabolism, Lundquist Research Institute at Harbor-UCLA Medical Center, Torrance, CA, USA. ^115^Department of Public Health and Caring Sciences, Uppsala University, Uppsala, Sweden. ^116^Centro de Estudios en Diabetes, Unidad de Investigacion en Diabetes y Riesgo Cardiovascular, Centro de Investigacion en Salud Poblacional, Instituto Nacional de Salud Publica, Mexico City, Mexico. ^117^Department of Medicine, Division of Endocrinology, Diabetes and Metabolism, Cedars-Sinai Medical Center, Los Angeles, CA, USA. ^118^Center for Epigenomics, University of California San Diego, La Jolla, CA, USA. ^119^Department of Laboratory Medicine and Pathology, University of Minnesota, Minneapolis, MN, USA. ^120^University of Exeter Medical School, University of Exeter, Exeter, UK. ^121^Institute for Clinical Diabetology, German Diabetes Center, Leibniz Center for Diabetes Research at Heinrich Heine University Düsseldorf, Düsseldorf, Germany. ^122^Department of Endocrinology and Diabetology, Medical Faculty and University Hospital Düsseldorf, Heinrich Heine University Düsseldorf, Düsseldorf, Germany. ^123^Department of Biostatistics, Gillings School of Global Public Health, University of North Carolina at Chapel Hill, Chapel Hill, NC, USA. ^124^Department of Internal Medicine, Diabetes and Metabolism Research Center, The Ohio State University Wexner Medical Center, Columbus, OH, USA. ^125^Center for Global Cardiometabolic Health, Brown University, Providence, RI, USA. ^126^Shanghai-MOST Key Laboratory of Health and Disease Genomics, Chinese National Human Genome Center at Shanghai (CHGC) and Shanghai Institute for Biomedical and Pharmaceutical Technologies (SIBPT), Shanghai, China. ^127^Division of Endocrine and Metabolism, Tri-Service General Hospital Songshan Branch, Taipei, Taiwan. ^128^School of Medicine, National Defense Medical Center, Taipei, Taiwan. ^129^Section of Endocrinology and Metabolism, Department of Medicine, Taipei Veterans General Hospital, Taipei, Taiwan. ^130^School of Medicine, National Yang Ming Chiao Tung University, Taipei, Taiwan. ^131^Department of Environmental and Preventive Medicine, Jichi Medical University School of Medicine, Shimotsuke, Japan. ^132^University of Chicago Research Bangladesh, Dhaka, Bangladesh. ^133^Institute of Molecular and Clinical Ophthalmology Basel, Basel, Switzerland. ^134^Steno Diabetes Center Copenhagen, Gentofte, Denmark. ^135^National Institute of Public Health, Southern Denmark University, Copenhagen, Denmark. ^136^Center for Clinical Research and Prevention, Bispebjerg and Frederiksberg Hospital, Frederiksberg, Denmark. ^137^Faculty of Health and Medical Sciences, University of Copenhagen, Copenhagen, Denmark. ^138^Faculty of Medicine, Aalborg University, Aalborg, Denmark. ^139^Laboratory of Complex Trait Genomics, Department of Computational Biology and Medical Sciences, Graduate School of Frontier Sciences, The University of Tokyo, Tokyo, Japan. ^140^Department of Clinical Diabetes, Endocrinology & Metabolism, Department of Translational Research and Cellular Therapeutics, City of Hope, Duarte, CA, USA. ^141^Department of Public Health, Faculty of Medicine, University of Kelaniya, Ragama, Sri Lanka. ^142^Department of Clinical Gene Therapy, Osaka University Graduate School of Medicine, Osaka, Japan. ^143^Department of Geriatric and General Medicine, Graduate School of Medicine, Osaka University, Osaka, Japan. ^144^Division of General Internal Medicine and Geriatrics, Department of Medicine, Northwestern University Feinberg School of Medicine, Chicago, IL, USA. ^145^Center for Health Information Partnerships, Institute for Public Health and Medicine, Northwestern University Feinberg School of Medicine, Chicago, IL, USA. ^146^Genome Institute of Singapore, Agency for Science, Technology and Research, Singapore, Singapore. ^147^Department of Molecular Cell Biology, Sungkyunkwan University School of Medicine, Suwon, South Korea. ^148^Department of Regional Resource Management, Ehime University Faculty of Collaborative Regional Innovation, Ehime, Japan. ^149^Institute of Genetic Epidemiology, Department of Genetics and Pharmacology, Medical University of Innsbruck, Innsbruck, Austria. ^150^Institute of Clinical Medicine, Internal Medicine, University of Eastern Finland and Kuopio University Hospital, Kuopio, Finland. ^151^Institute of Mathematics and Statistics, University of Tartu, Tartu, Estonia. ^152^Department of Medicine, University of Colorado Denver, Anschutz Medical Campus, Aurora, CO, USA. ^153^Severance Biomedical Science Institute and Department of Internal Medicine, Yonsei University College of Medicine, Seoul, South Korea. ^154^Department of Medicine, Samsung Medical Center, Sungkyunkwan University School of Medicine, Seoul, South Korea. ^155^USC-Office of Population Studies Foundation, Inc., University of San Carlos, Cebu City, Philippines. ^156^Department of Medicine, Harvard Medical School, Boston, MA, USA. ^157^Division of General Internal Medicine, Massachusetts General Hospital, Boston, MA, USA. ^158^Department of Epidemiology and Biostatistics, Peking University Health Science Centre, Peking University, Beijing, China. ^159^Department of Clinical Epidemiology, Leiden University Medical Center, Leiden, The Netherlands. ^160^Program in Medical & Population Genetics, Broad Institute, Cambridge, MA, USA. ^161^Big Data Institute, Li Ka Shing Centre for Health Information and Discovery, University of Oxford, Oxford, UK. ^162^Department of Clinical Medicine, Faculty of Health and Medical Sciences, University of Copenhagen, Copenhagen, Denmark. ^163^Department of Medicine, Yong Loo Lin School of Medicine, National University of Singapore and National University Health System, Singapore, Singapore. ^164^McDonnell Genome Institute, Washington University School of Medicine, St. Louis, MO, USA. ^165^Department of Medicine, Division of Genomics and Bioinformatics, Washington University School of Medicine, St. Louis, MO, USA. ^166^Department of Biostatistics and Data Science, University of Texas Health Science Center at Houston, Houston, TX, USA. ^167^Department of Clinical Sciences, Diabetes and Endocrinology, Lund University Diabetes Centre, Malmö, Sweden. ^168^Department of Clinical Science, Center for Diabetes Research, University of Bergen, Bergen, Norway. ^169^Dromokaiteio Psychiatric Hospital, National and Kapodistrian University of Athens, Athens, Greece. ^170^Institute of Human Genetics, Helmholtz Zentrum München, German Research Center for Environmental Health, Neuherberg, Germany. ^171^Institute of Human Genetics, Technical University of Munich, Munich, Germany. ^172^German Centre for Cardiovascular Research (DZHK), Partner Site Munich Heart Alliance, Munich, Germany. ^173^The Usher Institute to the Population Health Sciences and Informatics, University of Edinburgh, Edinburgh, UK. ^174^Digital Health Center, Digital Engineering Faculty of Hasso Plattner Institue and University Potsdam, Potsdam, Germany. ^175^The Division of Data Driven and Digital Medicine (D3M), Department of Medicine, Icahn School of Medicine at Mount Sinai, New York, NY, USA. ^176^Department of Medicine and Pharmacology, New York Medical College, Valhalla, NY, USA. ^177^Data Tecnica International LLC, Glen Echo, MD, USA. ^178^Center for Alzheimer’s and Related Dementias, National Institutes of Health, Baltimore, MD, USA. ^179^William Harvey Research Institute, Barts and The London School of Medicine and Dentistry, Queen Mary University of London, London, UK. ^180^Department of Statistical Genetics, Osaka University Graduate School of Medicine, Osaka, Japan. ^181^Laboratory of Statistical Immunology, Immunology Frontier Research Center (WPI-IFReC), Osaka University, Osaka, Japan. ^182^Laboratory for Systems Genetics, RIKEN Center for Integrative Medical Sciences, Yokohama, Japan. ^183^Instituto Nacional de Medicina Genómica, Mexico City, Mexico. ^184^Division of Pulmonary, Allergy, and Critical Care Medicine, Department of Medicine, University of Pittsburgh, Pittsburgh, PA, USA. ^185^Division of Epidemiology and Community Health, School of Public Health, University of Minnesota, Minneapolis, MN, USA. ^186^Department of Diabetes and Endocrinology, Nelson R Mandela School of Medicine, College of Health Sciences, University of KwaZulu-Natal, Durban, South Africa. ^187^Academy of Scientific and Innovative Research, CSIR-Human Resource Development Centre Campus, Ghaziabad, Uttar Pradesh, India. ^188^Genomics and Molecular Medicine Unit, CSIR-Institute of Genomics and Integrative Biology, New Delhi, India. ^189^Department of Preventive Medicine, Northwestern University Feinberg School of Medicine, Chicago, IL, USA. ^190^Fred Hutchinson Cancer Research Center, Seattle, WA, USA. ^191^Ophthalmology and Visual Sciences Academic Clinical Program (Eye ACP), Duke-NUS Medical School, Singapore, Singapore. ^192^Department of Ophthalmology, Yong Loo Lin School of Medicine, National University of Singapore and National University Health System, Singapore, Singapore. ^193^Department of Pediatrics, Pediatric Diabetes Research Center, University of California San Diego, La Jolla, CA, USA. ^194^Institute for Genomic Medicine, University of California San Diego, La Jolla, CA, USA. ^195^Department of Cellular and Molecular Medicine, University of California San Diego, La Jolla, CA, USA. ^196^Institute of Cardiovascular and Medical Sciences, University of Glasgow, Glasgow, UK. ^197^Hasso Plattner Institute for Digital Health at Mount Sinai, Icahn School of Medicine at Mount Sinai, New York, NY, USA. ^198^Survey Research Center, Institute for Social Research, University of Michigan, Ann Arbor, MI, USA. ^199^Institute of Genetic Epidemiology, Helmholtz Zentrum München, German Research Center for Environmental Health, Neuherberg, Germany. ^200^Chair of Genetic Epidemiology, IBE, Faculty of Medicine, LMU Munich, Munich, Germany. ^201^Institute of Medical Biostatistics, Epidemiology and Informatics (IMBEI), University Medical Center, Johannes Gutenberg University, Mainz, Germany. ^202^Department of Diabetes and Metabolic Diseases, Graduate School of Medicine, The University of Tokyo, Tokyo, Japan. ^203^Department of Genomic Medicine, National Cerebral and Cardiovascular Center, Osaka, Japan. ^204^Faculty of Medicine, University of Reykjavik, Reykjavik, Iceland. ^205^Faculty of Medicine, Macau University of Science and Technology, Macau, China. ^206^Department of Medical Genetics and Medical Research, China Medical University Hospital, Taichung, Taiwan. ^207^Department of Health, Finnish Institute for Health and Welfare, Helsinki, Finland. ^208^National School of Public Health, Madrid, Spain. ^209^Department of Neuroscience and Preventive Medicine, Danube-University Krems, Krems, Austria. ^210^Diabetes Research Group, King Abdulaziz University, Jeddah, Saudi Arabia. ^211^Unidad de Biología Molecular y Medicina Genómica, Instituto Nacional de Ciencias Médicas y Nutrición Salvador Zubirán, Mexico City, Mexico. ^212^Departamento de Medicina Genómica y Toxiología Ambiental, Instituto de Investigaciones Biomédicas, UNAM, Mexico City, Mexico. ^213^Unidad de Investigacion Medica en Bioquimica, Hospital de Especialidades, Centro Medico Nacional Siglo XXI, IMSS, Mexico City, Mexico. ^214^Einthoven Laboratory for Experimental Vascular Medicine, Leiden University Medical Center, Leiden, The Netherlands. ^215^Department of Human Genetics, Leiden University Medical Center, Leiden, The Netherlands. ^216^Department of Clinical Chemistry, Laboratory of Genetic Metabolic Disease, Amsterdam University Medical Center, Amsterdam, The Netherlands. ^217^Southern California Eye Institute, CHA Hollywood Presbyterian Medical Center, Los Angeles, CA, USA. ^218^Department of Medicine, University of Pennsylvania Perelman School of Medicine, Philadelphia, PA, USA. ^219^Unidad de Investigación Médica en Epidemiologia Clinica, Hospital de Especialidades, Centro Medico Nacional Siglo XXI, IMSS, Mexico City, Mexico. ^220^Department of Medicine, School of Medicine, University of North Carolina at Chapel Hill, Chapel Hill, NC, USA. ^221^Department of Internal Medicine, Division of Endocrinology, Leiden University Medical Center, Leiden, The Netherlands. ^222^Department of Public Health, Aarhus University, Aarhus, Denmark. ^223^Danish Diabetes Academy, Odense, Denmark. ^224^Diabetology Research Centre, King Edward Memorial Hospital and Research Centre, Pune, India. ^225^Department of Medical Biochemistry, Kurume University School of Medicine, Kurume, Japan. ^226^Institute for Molecular Bioscience, University of Queensland, Brisbane, Australia. ^227^Division of Cancer Control and Population Sciences, UPMC Hillman Cancer Center, University of Pittsburgh, Pittsburgh, PA, USA. ^228^Department of Epidemiology, Graduate School of Public Health, University of Pittsburgh, Pittsburgh, PA, USA. ^229^Department of Pediatrics, Division of Genetic and Genomic Medicine, UCI Irvine School of Medicine, Irvine, CA, USA. ^230^Department of Anti-aging Medicine, Ehime University Graduate School of Medicine, Ehime, Japan. ^231^Division of Pulmonary, Critical Care, and Sleep Medicine, Beth Israel Deaconess Medical Center, Boston, MA, USA. ^232^Department of Medical Sciences, Uppsala University, Uppsala, Sweden. ^233^Human Genetics Center, University of Texas Health Science Center at Houston, Houston, TX, USA. ^234^Department of Medicine, Division of Cardiovascular Medicine, Stanford University School of Medicine, Stanford, CA, USA. ^235^Department of Medical Sciences, Molecular Epidemiology and Science for Life Laboratory, Uppsala University, Uppsala, Sweden. ^236^Department of Epidemiology, University of Washington, Seattle, WA, USA. ^237^Department of Health Services, University of Washington, Seattle, WA, USA. ^238^Beijing Institute of Ophthalmology, Ophthalmology and Visual Sciences Key Laboratory, Beijing Tongren Hospital, Capital Medical University, Beijing, China. ^239^Department of Medicine, Division of Cardiology, Duke University School of Medicine, Durham, NC, USA. ^240^Technical University of Munich (TUM) and Klinikum Rechts der Isar, TUM School of Medicine, Munich, Germany. ^241^Kurume University School of Medicine, Kurume, Japan. ^242^Center for Public Health Genomics, University of Virginia School of Medicine, Charlottesville, VA, USA. ^243^Department of Medicine, McGill University, Montreal, QC, Canada. ^244^Department of Human Genetics, McGill University, Montreal, QC, Canada. ^245^Department of Genomics of Common Disease, School of Public Health, Imperial College London, London, UK. ^246^Department of Physiology and Biophysics, University of Mississippi Medical Center, Jackson, MS, USA. ^247^Division of Endocrinology and Metabolism, Department of Medicine, Taichung Veterans General Hospital, Taichung, Taiwan. ^248^Center for Genetic Medicine, Northwestern University Feinberg School of Medicine, Chicago, IL, USA. ^249^Department of Anthropology, Northwestern University, Evanston, IL, USA. ^250^Center for Precision Health Research, National Human Genome Research Institute, National Institutes of Health, Bethesda, MD, USA. ^251^Systems Genomics Laboratory, School of Biotechnology, Jawaharlal Nehru University, New Delhi, India. ^252^Department of Pathology and Molecular Medicine, McMaster University, Hamilton, ON, Canada. ^253^Department of Internal Medicine, Seoul National University College of Medicine, Seoul, South Korea. ^254^Department of Molecular Medicine and Biopharmaceutical Sciences, Graduate School of Convergence Science and Technology, Seoul National University, Seoul, South Korea. ^255^Netherlands Heart Institute, Utrecht, The Netherlands. ^256^MRC-PHE Centre for Environment and Health, Imperial College London, London, UK. ^257^Duke-NUS Medical School, Singapore, Singapore. ^258^Singapore Institute for Clinical Sciences, Agency for Science Technology and Research (A*STAR), Singapore, Singapore. ^259^Healthy Longevity Translational Research Programme, Yong Loo Lin School of Medicine, National University of Singapore, Singapore, Singapore. ^260^Pat Macpherson Centre for Pharmacogenetics and Pharmacogenomics, University of Dundee, Dundee, UK. ^261^Department of Medicine, Brown University Alpert School of Medicine, Providence, RI, USA. ^262^Imperial College Healthcare NHS Trust, Imperial College London, London, UK. ^263^National Heart and Lung Institute, Imperial College London, London, UK. ^264^The Mindich Child Health and Development Institute, Ichan School of Medicine at Mount Sinai, New York, NY, USA. ^265^Department of Biostatistics and Epidemiology, University of Pennsylvania, Philadelphia, PA, USA. ^266^Center for Non-Communicable Diseases, Karachi, Pakistan. ^267^Computational Medicine, Berlin Institute of Health at Charité Universitätsmedizin, Berlin, Germany. ^268^Department of Advanced Genomic and Laboratory Medicine, Graduate School of Medicine, University of the Ryukyus, Okinawa, Japan. ^269^Division of Clinical Laboratory and Blood Transfusion, University of the Ryukyus Hospital, Okinawa, Japan. ^270^Department of Statistics, University of Oxford, Oxford, UK. ^271^Section of Genetics and Genomics, Department of Metabolism, Digestion and Reproduction, Imperial College London, London, UK. ^272^Oxford NIHR Biomedical Research Centre, Churchill Hospital, Oxford University Hospitals NHS Foundation Trust, Oxford, UK. ^273^Division of Endocrinology, Department of Pediatrics, Stanford School of Medicine, Stanford University, Stanford, CA, USA. ^274^Center for Diabetes Research, Wake Forest School of Medicine, Winston-Salem, NC, USA. ^275^Centre for Genetics and Genomics Versus Arthritis, Centre for Musculoskeletal Research, Division of Musculoskeletal and Dermatological Sciences, University of Manchester, Manchester, UK. ^276^NIHR Manchester Biomedical Research Centre, Manchester University NHS Foundation Trust, Manchester, UK. ^277^Deceased. ^278^Present address: Genentech, South San Francisco, CA, USA. ^279^Present address: Internal Medicine Research Unit, Pfizer Worldwide Research, Cambridge, MA, USA. ^280^Present address: Departamento de Medicina Genómica y Toxicología Ambiental, Instituto de Investigaciones Biomédicas, UNAM, Ciudad de Mexico, Mexico. ^281^Present address: The University of Texas Health Science Center at Houston, School of Public Health, Department of Epidemiology, Human Genetics, and Environmental Sciences, Houston, TX, USA. ^282^Present address: Institute of Data Science, Korea University, Seoul, South Korea. ^283^Present address: Division of Academics, Ochsner Health, New Orleans, LA, USA. ^284^Present address: Vertex Pharmaceuticals Ltd, Oxford, UK. ^285^Present address: Exeter Centre of Excellence in Diabetes (ExCEeD), Exeter Medical School, University of Exeter, Exeter, UK. ^286^Present address: Center for Precision Health Research, National Human Genome Research Institute, National Institutes of Health, Bethesda, MD, USA. ^287^Present address: Ibusuki Kozenkai Hospital, Ibusuki, Japan. ^288^Present address: Regeneron Genetics Center, Tarrytown, NY, USA ^289^Present address: Institute for Population and Precision Health (IPPH), Biological Sciences Division, The University of Chicago, Chicago, IL, USA. ^290^Present address: Clinical Trial Service Unit and Epidemiological Studies Unit, Nuffield Department of Population Health, University of Oxford, Oxford, UK. ^291^Present address: Toranomon Hospital, Tokyo, Japan.

Data used in this article were generated by the Diabetes Meta-Analysis of Trans-Ethnic association studies (DIAMANTE) Consortium. The investigators within the DIAMANTE provided data but did not participate in the analysis or writing of this report.
